# Inequalities in hormone replacement therapy prescribing in UK primary care: population based cohort study

**DOI:** 10.1136/bmjmed-2025-001349

**Published:** 2025-09-25

**Authors:** Jennifer A Hirst, Wema Meranda Mtika, Carol Coupland, Sharon Dixon, Julia Hippisley-Cox, Sarah Hillman

**Affiliations:** 1 Nuffield Department of Primary Care Health Sciences, University of Oxford, Oxford, UK; 2 Wolfson Institute of Population Health, Queen Mary University of London, London, UK; 3 University of Nottingham, Nottingham, UK; 4 Nuffield Department of Primary Health Care Sciences, University of Oxford, Oxford, UK; 5 School of Health Sciences, University of Birmingham, Birmingham, UK

**Keywords:** Hormone replacement therapy, Primary health care, Epidemiology

## Abstract

**Objective:**

To quantify prescribing of hormone replacement therapy (HRT) in women aged 40-60 years by type of HRT and length of use, and to determine sociodemographic factors associated with receiving a HRT prescription.

**Design:**

Population based cohort study.

**Setting:**

QResearch database of primary care practices in England, 1 January 2013 to 13 July 2023, and patient electronic health records for prescribing information .

**Participants:**

1 978 348 women aged 40-60 years at any time over a 10 year period.

**Main outcome measures:**

Overall uptake of two or more prescriptions of the same type of HRT in women of menopausal age, length of use, and association between ethnic group, deprivation, and geographical region and receiving a HRT prescription before and during the eight years since implementation of National Institute for Health and Care Excellence (NICE) guidance on the menopause in 2015 in the UK.

**Results:**

The cohort comprised 1 978 348 women with a mean age of 49.4 years, and 76.2% were white women. Overall, 379 911 (19.2%) women received two or more HRT prescriptions. Combination HRT formulations in one prescription were the most frequently prescribed (62.4% of those prescribed HRT), with 43.3% receiving oral and 26.3% transdermal formulations. Mean age at first prescription was 49.8 years. Rates for two or more prescriptions of HRT were higher in white women (22.6%) than in other ethnic groups, ranging from 8.9% in Caribbean women to 3.9% in black African women. Prescription rates decreased with increasing social deprivation, from 24.2% in the most affluent to 10.9% in the most deprived groups. London had lower prescription rates (11.7%) than other regions (all >19%). Multivariable Cox regression showed that non-white ethnic groups had significantly lower HRT prescription rates (hazard ratios 0.85-0.92, P<0.001), and each increase in social deprivation group was associated with lower HRT prescription rates (hazard ratio for the most deprived group 0.92, 95% confidence interval 0.92 to 0.93, P<0.001).

**Conclusions:**

This study identified differences in HRT prescribing in England based on ethnic group, socioeconomic status, and geographical location. White women and those in more affluent neighbourhoods were more likely to receive HRT than non-white women and those in more deprived areas. These findings suggest potential inequities that require further exploration.

**WHAT IS ALREADY KNOWN ON THIS TOPIC**

- Known inequalities exist in the prescribing of hormone replacement therapy (HRT) in the UK, with areas of highest deprivation having the lowest HRT prescribing rates
- No previous study has reported the extent of the differences in individual HRT prescribing rates by both deprivation and ethnic group

**WHAT THIS STUDY ADDS**

- At an individual level in England, significant differences were found in those receiving at least two HRT prescriptions based on ethnic group, socioeconomic status, and region
- White women and those from affluent neighbourhoods were more likely to receive two HRT prescriptions
- Non-white women and those in deprived areas had significantly lower prescription rates

**HOW THIS STUDY MIGHT AFFECT RESEARCH, PRACTICE, OR POLICY**

- These inequalities in HRT prescribing, that persisted even after adjusting for other sociodemographic factors, suggest potential inequities in access and different levels of HRT uptake
- Further exploration of HRT provision is needed

## Introduction

 Symptoms of the menopause usually last several years and can affect sleep and quality of life, with symptoms ranging from tolerable to markedly disruptive.^1^ Hormone replacement therapy (HRT) is known to be effective at treating problematic vasomotor symptoms of menopause.^2^ Over the years, HRT prescribing rates have varied, with rates dropping^3 4^ after the Women’s Health Initiative publication in 2002 reporting adverse health outcomes,^5^ but more recently, HRT prescribing has increased hugely.^6 7^ The UK’s National Institute for Health and Care Excellence (NICE) published its first ever guidance on managing the menopause in 2015,^8^ recommending an individualised approach to treatment and with tables to help explain risks.^9^ The NICE menopause guidance was updated in 2024^10^ and included a discussion aid on HRT and the likelihood of some medical conditions to support doctors.^11^

The Study of Women’s Health Across the Nation (SWAN) in the US followed up >3000 women from 1995, and reported that in black women, menopause occurred eight months earlier than in white women.^12^ The study also found a higher burden and length of symptoms, including vasomotor symptoms (hot flashes and night sweats), clinically significant depressive symptoms, and lower quality of life in black than in white women.^13^ Recently, data from the US have reported that women from black ethnic groups reported greater symptom severity than white women, even after adjusting for socioeconomic status,^14^ and African American women had a longer length of symptoms (>10 years) than other ethnic groups.^1^ The evidence therefore suggests that HRT might be clinically indicated in a higher proportion of black women in the US.

Although these findings cannot be generalised to other countries, differences by ethnic group likely persist. Other factors reported to be associated with increasing severity of symptoms of menopause include obesity, being a current smoker,^15^ having diabetes,^16^ and living in a more deprived neighbourhood.^17^ We know that smoking rates,^18^ obesity,^19 20^ and the prevalence of type 2 diabetes^21^ are highest in more socioeconomically deprived areas, suggesting that we would expect the severity of symptoms of menopause and clinical need for HRT to be highest in areas with high poverty rates. Furthermore, social and economic inequalities are fundamental causes of ethnic inequalities in health.^22^

Known inequalities exist in HRT prescribing in the UK, with areas of highest deprivation having the lowest HRT prescribing rates.^23^ The reasons for these inequalities are not understood, but contributing factors in the community and clinical settings could be lack of awareness or communication about menopause, for example. Also, uncertainty may arise about the risks and benefits of available treatments among clinicians and women, which could affect which treatments are offered or requested. In 1998, a UK survey reported higher HRT use in white women than in those of South Asian or African descent.^24^ Critical questions remain, including whether HRT uptake varies in Asian and black ethnic groups, how ethnic group and social deprivation are related to uptake of HRT, and what is the burden of comorbidities among those prescribed HRT compared with those not prescribed HRT.

UK data have also shown that general practices in affluent areas are more likely to prescribe transdermal HRT whereas in deprived areas, a higher proportion of oral HRT is prescribed.^23^ Because the burden of cardiovascular disease is higher in deprived than in affluent areas,^25^ and transdermal preparations are recommended for women with a higher risk of thromboembolism or stroke,^10^ higher rates of prescribing of transdermal preparations would be expected in practices serving the most deprived populations. Whether women with the most severe symptoms are those who are prescribed HRT is not known. Also, whether there may be unmet need in some communities, specifically women from more deprived areas or women from minority ethnic backgrounds who might have limited access to treatment, is unclear. The aim of our study was to determine the overall uptake of HRT in women of menopausal age, and describe the uptake of different types of HRT prescribed and length of use. We also wanted to establish whether ethnic group, deprivation, and geographical region were associated with receiving a HRT prescription before and during the eight years since implementation of the first NICE guidance on the menopause in 2015^8^ in the UK.

## Methods

The study followed an open cohort from the QResearch database, which has health and prescribing records for >35 million patients, derived from about 1500 practices in England from Optum (formerly EMIS Health).^26^ Women aged 40-60 years at any time over a 10 year period (1 January 2013 to 13 July 2023) were eligible for inclusion in the study; menopause and its symptoms are most likely in this age range.^10 27^ Data for sex were taken from information in the QResearch database rather than from patient reported gender. Women were followed up from the date of study entry (defined as the latest of 1 January 2013, date of joining the practice, or date of their 40th birthday) until the earliest of 13 July 2023, the date they left the practice, or the date they died.

In this study, HRT uptake was defined as women who had at least two prescriptions^28^ (as an indicator of likely use) of the same type of HRT from age 40 years (or date of registration with the practice if later) at any time before study entry or during follow-up. Box 1 shows the individual types and combinations of HRT formulations assessed. For those included in the cohort, numbers and proportions of women with comorbidities, risk factors, and conditions which might affect HRT uptake recorded in healthcare records before the study entry date were reported. We described HRT prescribing grouped by self-assigned ethnic group, as recorded on general practice records, with hospital records used to supplement missing data. The nine ethnic groups were white, Indian, Pakistani, Bangladeshi, other Asian, Caribbean, black African, Chinese, and other. Socioeconomic status was measured with the Townsend deprivation score based on individual postcodes, where higher levels indicated higher levels of deprivation.^29^ We have separately reported the proportion of the cohort who received one or more HRT prescriptions and the proportion who received three or more prescriptions. We have also reported the characteristics of those with a minimum of five years of follow-up and in those aged ≥45 years at cohort entry for comparison with the full cohort in total and with those who received two or more HRT prescriptions.

Box 1 **Individual hormone replacement therapy (HRT) groups**

Any HRT

Any oestrogen

- Oral oestrogen
- Transdermal oestrogen
- Conjugated oestrogen
- Topical vaginal oestrogen
- Implant oestrogen

Any oestrogen-progestogen

- Oral oestrogen-progestogen
- Transdermal oestrogen-progestogen

Any progestogen

- Oral progestogen
- Vaginal progestogen
- Progestogen by intrauterine device

Tibolone

We calculated the uptake of different HRT formulations categorised into different groups, as described by previous researchers^30 31^ and reviewed by clinicians on the study team, that included older and newer formulations, and oestrogen only, oestrogen-progesterone, and tibolone formulations.^30^ We also calculated HRT uptake in women who were taking their oestrogen and progesterone separately (ie, oral or transdermal oestrogen and progesterone in the form of an intrauterine progesterone only device (52 mg levonorgestrel) or oral forms of progesterone) and for different modes routes of delivery (ie, oral or transdermal preparations of HRT).

Comorbidities were defined as any specified condition that was coded in the general practice records any time before the first prescription of HRT or, in those without a HRT prescription, at the date of study entry. To compare the route of administration in oestrogen or progestogen combined formulations (transdermal *v* oral), each woman with a prescription for the transdermal or oral form was identified. For those who had received prescriptions for both, the form of administration with the longest length between the first and last prescription was used.

### Statistical methods

The variables studied were Townsend deprivation score, ethnic group, and geographical region of England. Our main outcome was two or more prescriptions of the same type of HRT, from age 40 years to the end of follow-up, in women who were aged 40-60 years during the 10 year follow-up period from 2013 to 2023.

Numbers and characteristics of women taking different types, doses, and routes of administration of HRT are reported, as well as length of use of HRT. Mean age and standard deviation (SD) at the first HRT prescription are also reported. Length of use by type of HRT is presented as median and interquartile range (IQR). We generated tables and graphs to report personal characteristics (age at study entry, ethnic group, Townsend deprivation score (divided into five equal groups), and geographical region of England, subdivided into 10 geographical regions) with appropriate summary statistics for the full cohort of women and women taking any formulation or dose of HRT. Prescriptions of any type of HRT, and different types of HRT and routes of administration were tabulated by Townsend deprivation group and ethnic group. Proportions of women with comorbid conditions were also described based on whether they received a HRT prescription. A missing indicator variable (not reported) was generated for those with missing data for ethnic group and deprivation, and used for descriptive and statistical analyses.

To identify factors associated with receiving two or more HRT prescriptions from age 40 years, we used a Cox proportional hazards regression as our primary analysis, reporting hazard ratios and 95% confidence intervals (CIs) to account for different lengths of follow-up for individuals after feedback from reviewers. As a secondary prespecified analysis, we used logistic regression (see protocol in online supplemental materials) to report odds ratios of receiving two or more HRT prescriptions from age 40 years. In post hoc sensitivity analyses, we used multivariable Cox regression models to see if HRT prescription patterns were different in those who received two or more or three or more HRT prescriptions and for prescriptions issued after the publication of the first NICE menopause guidance in 2015,^8^ by restricting the analysis to those who received a first HRT prescription after 1 January 2016. In other sensitivity analyses, we used a multinomial logistic regression model to identify factors associated with uptake of one prescription, and those associated with uptake of two or more prescriptions compared with no prescription for different types of HRT and routes of administration. In the multilevel logistic regression model, we used general practice as a random effect to account for similar prescribing behaviours within individual practices. To compare routes of administration for oestrogen, we created a binary variable (transdermal or oral) based on the route of administration used for the longest time by the individual, where data were available. We used multivariable logistic regression to compare the characteristics of those prescribed transdermal versus oral prescriptions of oestrogen only formulations and combined oestrogen-progestogen prescriptions.

Cox regression was used to compare HRT prescriptions in those who had received a diagnosis of a health condition before their first HRT prescription and those who did not have a diagnosis of any of the conditions in unadjusted models. We also compared HRT prescriptions by body mass index at cohort entry. Stata version 18MP (StataCorp, TX) was used for the data analyses. Significance was assumed at the 1% level.

### Patient and public involvement

We were not able to share our results with research participants because we used anonymised health records. A patient co-applicant supported us in getting funding for this study and has continued to be involved in study management meetings. We recruited an ethnically and socially diverse group of five women who helped us make sense of the results and are supporting us with finding ways to communicate these results to the communities who need this information the most. We have created short text summaries and infographics of our results which will be shared with patients through support groups and charities.

## Results

The full cohort comprised 1 978 348 women with a mean age in 2013 of 49.4 years (SD 6.0, median 49, IQR 44-54 years). Age ranged from 48.7 years in London to 49.8 years in the South West region of England. Table 1 shows the characteristics of the cohort. Ethnic group was recorded in 1 862 794 (94.2%) participants: 76.2% were white ethnic group, 3.0% Indian, 1.7% Pakistani, 0.8% Bangladeshi, 1.9% other Asian, 1.7% Caribbean, 3.4% black African, 1.0% Chinese, 4.4% other ethnic group, and 5.8% ethnic group not recorded. Compared with the 2021 UK census data,^32^ these values were broadly similar to the general UK population in the same age group (online supplemental table 1). A higher proportion of the cohort was in the most affluent Townsend deprivation group (group 1, 27.3%) compared with 13.3% in the most deprived group (group 5). The largest proportion of the cohort (25.1%) was from London with the smallest proportion (1.8%) from the East Midlands (table 1).

**Table 1:** Cohort characteristics of women aged 40-60 years at study entry who received no hormone replacement therapy (HRT) or any HRT formulation (two or more prescriptions of HRT)

	Mean (SD) follow-up (years)	Total No of women (n=1 978 348)	No HRT (n=1 598 437)	Any HRT formulation (n=379 911)
Median (IQR) age at cohort entry (years)	—	49 (44-54)	49 (44-54)	50 (46-55)
Mean (SD) age at cohort entry (years)	—	49.4 (6.0)	49.2 (6.0)	50.2 (5.6)
Mean (SD) age at first HRT prescription (years)	—	—	—	49.8 (5.4)
Age (years)				
40-44	—	—	—	45 602 (12.6)
45-49	—	—	—	120 211 (33.1)
50-54	—	—	—	146 935 (40.5)
55-60	—	—	—	50 321 (13.9)
Ethnic group				
White	6.7 (3.5)	1 506 788 (76.2)	1 166 354 (73.0)	340 434 (89.6)
Indian	6.2 (3.5)	60 142 (3.0)	54 929 (3.4)	5213 (1.4)
Pakistani	6.4 (3.5)	33 909 (1.7)	31 604 (2.0)	2305 (0.6)
Bangladeshi	6.5 (3.6)	16 579 (0.8)	15 558 (1.0)	1021 (0.3)
Other Asian	5.6 (3.5)	38 279 (1.9)	36 218 (2.3)	2061 (0.5)
Caribbean	7.0 (3.4)	32 951 (1.7)	30 011 (1.9)	2940 (0.8)
Black African	5.6 (3.5)	67 320 (3.4)	64 670 (4.0)	2650 (0.7)
Chinese	5.3 (3.6)	19 651 (1.0)	18 832 (1.2)	819 (0.2)
Other	5.5 (3.5)	87 175 (4.4)	79 137 (5.0)	8038 (2.1)
Not recorded	6.0 (3.6)	115 554 (5.8)	101 124 (6.3)	14 430 (3.8)
Townsend deprivation group				
1 (most affluent)	6.9 (3.4)	540 506 (27.3)	409 829 (25.6)	130 677 (34.4)
2	6.6 (3.5)	464 548 (23.5)	362 955 (22.7)	101 593 (26.7)
3	6.4 (3.5)	380 182 (19.2)	310 804 (19.4)	69 378 (18.3)
4	6.2 (3.5)	313 471 (15.8)	267 253 (16.7)	46 218 (12.2)
5 (most deprived)	6.1 (3.6)	263 650 (13.3)	234 869 (14.7)	28 781 (7.6)
Not recorded	4.6 (3.5)	15 991 (0.8)	12 727 (0.8)	3264 (0.9)
Region				
East Midlands	7.4 (3.5)	35 896 (1.8)	28 305 (1.8)	7591 (2.0)
East of England	6.7 (3.4)	83 327 (4.2)	65 633 (4.1)	17 694 (4.7)
London	5.8 (3.6)	495 675 (25.1)	437 872 (27.4)	57 803 (15.2)
North East	8.1 (3.3)	43 885 (2.2)	34 810 (2.2)	9075 (2.4)
North West	7.0 (3.4)	377 130 (19.1)	293 690 (18.4)	83 440 (22.0)
South Central	6.7 (3.4)	262 736 (13.3)	205 865 (12.9)	56 871 (15.0)
South East	5.7 (3.4)	257 113 (13.0)	201 076 (12.6)	56 037 (14.8)
South West	6.8 (3.5)	172 532 (8.7)	132 011 (8.3)	40 521 (10.7)
West Midlands	6.7 (3.4)	199 041 (10.1)	159 473 (10.0)	39 568 (10.4)
Yorkshire and Humber	7.6 (3.4)	51 013 (2.6)	39 702 (2.5)	11 311 (3.0)
HRT prescriptions*				
Any HRT prescription	—	—	—	379 911 (19.2)
Any oestrogen prescription	—	—	—	192 484 (50.7)
Oral oestrogen	—	—	—	57 384 (15.1)
Transdermal oestrogen	—	—	—	151 552 (39.9)
Conjugated oestrogen	—	—	—	21 475 (5.7)
Topical vaginal oestrogen	—	—	—	81 (0.0)
Implant oestrogen	—	—	—	41 (0.0)
Any oestrogen-progestogen in one prescription	—	—	—	237 205 (62.4)
Oral oestrogen-progestogen (one prescription)	—	—	—	164 553 (43.3)
Transdermal oestrogen-progestogen (one prescription)	—	—	—	99 852 (26.3)
Any progestogen prescription	—	—	—	184 076 (48.5)
Oral progestogen	—	—	—	111 661 (29.4)
Vaginal progestogen	—	—	—	9613 (2.5)
Progestogen by intrauterine device	—	—	—	181 358 (47.7)
Tibolone	—	—	—	3978 (1.0)

Data are number (%) unless indicated otherwise.

* HRT prescription rows are not mutually exclusive.

IQR, interquartile range; SD, standard deviation.

A total of 379 911 (19.2%) women in the full cohort received two or more HRT prescriptions at any time (oestrogen only, oestrogen-progestogen combined formulations, or oestrogen and progestogen (oral or by intrauterine device) prescribed separately); 458 803 (23.2%) women received at least one HRT prescription and 340 042 (17.2%) received three or more HRT prescriptions. Table 1 shows the numbers and proportion of the cohort who received at least two prescriptions of different HRT formulations. Of those who were prescribed HRT, 237 205 (62.4%) received oestrogen and progestogens in one prescription, with 164 553 (43.3%) prescribed oral formulations and 99 852 (26.3%) transdermal formulations. Some women (8.0%) received prescriptions for both oral and transdermal formulations. Of 192 484 (50.7%) women who received a prescription for an oestrogen only formulation, a higher proportion received transdermal oestrogens (39.9% of all women on HRT) compared with oral oestrogen (15.1% of all women on HRT). Some women (4.8%) received prescriptions for both oral and transdermal formulations.

Mean age at first prescription of HRT was 49.8 (SD 5.4) years. When we restricted our analysis to prescriptions from 2016 (n=205 909), 125 014 (60.7%) women received combined oestrogen and progestogen in one prescription, with 31% of those with a HRT prescription receiving an oral combined formulation and 40% a transdermal combined formulation. Some women (6.9%) received prescriptions for both oral and transdermal formulations. In the cohort, 121 285 (58.9%) women received a prescription for an oestrogen only formulation after 2016 and a higher proportion of women received transdermal oestrogens (56.4% of all women on HRT) than oral oestrogen (6.7% of all women on HRT). Some women (2.0%) received prescriptions for both oral and transdermal formulations.

Table 1 shows the characteristics of the cohort grouped by whether they received any type of HRT and mean follow-up time. The proportion of women with HRT prescriptions was higher in white populations (340 434, 22.6% of all white women) than in other ethnic groups: 2940 (8.9%) Caribbean women, 5213 (8.7%) Indian women, 2305 (6.8%) Pakistani women, 1,021 (6.2%) Bangladeshi women, 2,061 (5.4%) other Asian women, 819 (4.2%) Chinese women, 2,650 (3.9%) black African women, 8,038 (9.2%) other group, and 14 430 (12.5%) did not have an ethnic group recorded. We saw a trend towards lower prescription rates with increasing social deprivation, ranging from 24.4% of total numbers in the most affluent group to 11.0% in the most deprived group. The proportion of HRT prescriptions was lower in London (11.7%) than all other regions of England (all >20%). For those with a minimum of five years of follow-up and those aged ≥45 years at cohort entry, the proportions with two or more HRT prescriptions were 22.5% and 23.1%, respectively. Online supplemental table 2 shows the characteristics of these cohorts by HRT prescriptions.

Online supplemental table 3 shows the characteristics of the study population who were prescribed two or more doses of different HRT formulations by ethnic group. The proportion of white women prescribed HRT was higher than in any other ethnic group for all HRT formulations and oestrogen only prescriptions. For example, 11.6% (n=174 628) of white women received two or more prescriptions of oestrogen only formulations compared with 4.5% (n=1488) in the Caribbean group, 4.2% (n=2545) in the Indian group, 2.8% (n=934) in the Pakistani group, 2.4% (n=915) in the other Asians group, 1.8% (n=356) in the Chinese group, 1.8% (n=301) in the Bangladeshi group, and 1.5% (n=1021) in the black African group. Prescriptions of oestrogens in those defined as other ethnic group and with an unrecorded ethnic group were 4.4% (n=3818) and 5.6% (n=6478), respectively. These patterns were similar for all types of HRT and routes of administration. As a proportion of all women who received two or more prescriptions, 22.6% of white women received only one prescription of oestrogen (39 459/174 628) compared with other ethnic groups: 50.2% in Bangladeshi (151/301) and 51.4% in black African populations (525/1021). This pattern was similar for all formulations and routes of administration (online supplemental table 4).

Online supplemental table 5 presents the characteristics of the study population who were prescribed two or more doses of different HRT formulations by social deprivation group. We saw a trend for higher prescribing in the most affluent group (group 1) compared with the most deprived group (group 5) for all HRT formulations. For example, 3.4% (n=18 437) of those in the most affluent group were prescribed oral oestrogen only formulations versus 1.7% (n=4362) of those in the most deprived group. For transdermal oestrogens, 10.5% (n=56 622) of those in the most affluent group received a prescription compared with 3.4% (n=8938) in the most deprived group. For each type of HRT, a higher proportion of women from the most deprived group received one prescription but did not then receive a second prescription compared with the most affluent group (29.4% *v* 26.5% for oral oestrogen). This pattern was similar for all formulations and routes of administration (online supplemental table 6).

Online supplemental table 7 shows length of use of HRT in those who received at least two prescriptions of the same type of HRT. Data are presented as median (IQR) days from the date of the first to the final prescription for each formulation, grouped by ethnic group. For any oestrogen prescriptions, median length of use was 657 days (IQR 257-1834) in white women, 816 days (235-2054) in Caribbean women, 633 (198-1668) days in other Asian populations, and <600 days in all other ethnic groups. Chinese women had the shortest length of oestrogen use (median 465, IQR 182-1304). Length of use by ethnic group varied by individual formulation.

**Figure 1:**
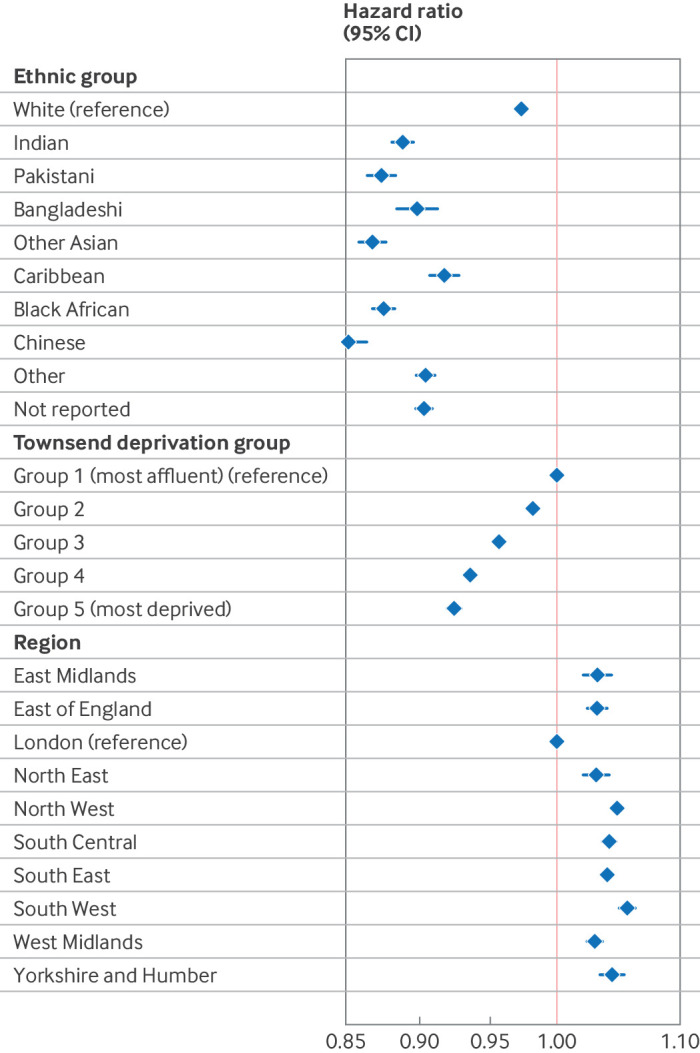
Forest plot showing adjusted hazard ratios and 95% confidence intervals (CIs) from multivariable Cox regression analysis of two or more hormone replacement therapy prescriptions, adjusted for age, ethnic group, deprivation (measured by the Townsend deprivation score, divided into five equal groups, from group 1 (most affluent) to group 5 (most deprived)), and region of England

For the univariable and multivariable Cox regression analyses (table 2 and figure 1), we found that the hazard ratio for receiving two or more prescriptions of any type of HRT was significantly lower (P<0.001) in all other ethnic groups than in white women. Adjusted hazard ratios ranged from 0.92 (95% CI 0.91 to 0.93) in Caribbean populations to 0.85 (0.84 to 0.86) in Chinese populations. For each increase in social deprivation group, the hazard ratio of having two or more HRT prescriptions decreased significantly. Compared with the most affluent group, those in the most deprived group had an adjusted hazard ratio of 0.92 (95% CI 0.92 to 0.93) of being prescribed HRT. Compared with London, prescription of HRT was significantly higher (P<0.001) in all other regions of England, with the highest adjusted hazard ratio of 1.06 (95% CI 1.05 to 1.06) in the South West. Multivariable logistic regression showed similar patterns, although odds ratios were more extreme, ranging from 0.48 (95% CI 0.46 to 0.50) in Caribbean women to 0.18 (0.17 to 0.19) in Chinese women compared with white women, and 0.59 (0.58 to 0.59) for the most deprived compared with the most affluent group (online supplemental table 8).

**Table 2:** Multivariable Cox regression for two or more prescriptions of any type of hormone replacement therapy (HRT). Multivariable hazard ratios are adjusted for ethnic group, Townsend deprivation group, and region of England

	Unadjusted hazard ratio (95% CI)	P value	Multivariable hazard ratio (95% CI)	P value
Ethnic group				
White	1.00	<0.001	1.00	<0.001
Indian	0.87 (0.86 to 0.87)		0.89 (0.88 to 0.90)	
Pakistani	0.85 (0.84 to 0.86)		0.87 (0.86 to 0.88)	
Bangladeshi	0.85 (0.83 to 0.86)		0.90 (0.88 to 0.91)	
Other Asian	0.84 (0.83 to 0.85)		0.87 (0.86 to 0.88)	
Caribbean	0.87 (0.86 to 0.88)		0.92 (0.91 to 0.93)	
Black African	0.83 (0.82 to 0.84)		0.88 (0.87 to 0.88)	
Chinese	0.83 (0.82 to 0.84)		0.85 (0.84 to 0.86)	
Other	0.87 (0.87 to 0.88)		0.90 (0.90 to 0.91)	
Missing	0.90 (0.89 to 0.91)		0.90 (0.90 to 0.91)	
Townsend deprivation group				
1 (most affluent)	1.00	<0.001	1.00	<0.001
2	0.97 (0.97 to 0.98)		0.98 (0.98 to 0.99)	
3	0.94 (0.93 to 0.94)		0.96 (0.95 to 0.96)	
4	0.90 (0.90 to 0.91)		0.94 (0.93 to 0.94)	
5 (most deprived)	0.87 (0.86 to 0.87)		0.92 (0.92 to 0.93)	
Region				
London (reference)	1.00	<0.001	1.00	<0.001
East Midlands	1.10 (1.09 to 1.11)		1.03 (1.02 to 1.04)	
East of England	1.10 (1.10 to 1.11)		1.03 (1.02 to 1.04)	
North East	1.10 (1.08 to 1.11)		1.03 (1.02 to 1.04)	
North West	1.11 (1.11 to 1.12)		1.05 (1.04 to 1.05)	
South Central	1.11 (1.10 to 1.11)		1.04 (1.04 to 1.05)	
South East	1.11 (1.10 to 1.11)		1.04 (1.03 to 1.05)	
South West	1.13 (1.13 to 1.14)		1.06 (1.05 to 1.06)	
West Midlands	1.09 (1.08 to 1.09)		1.03 (1.02 to 1.04)	
Yorkshire and Humber	1.11 (1.10 to 1.12)		1.04 (1.03 to 1.05)	

CI, confidence interval.

Online supplemental table 9 shows sensitivity analyses for one or more HRT prescriptions, or three or more HRT prescriptions. The patterns were similar to the primary analysis of two or more prescriptions of HRT, with small differences in the hazard ratios.

In the sensitivity analysis restricted to analysis of prescriptions of any HRT after 1 January 2016 (online supplemental table 10), we found similar results to the main analysis, but all hazard ratios for HRT prescriptions were slightly higher for each ethnic group than for the white ethnic group and for each deprivation group than the most affluent group. For example, compared with white women, the adjusted hazard ratios for prescriptions for any HRT were 0.89 for Indian women in the main analysis and 0.94 for prescriptions after 2016, for black African women, these values were 0.88 in the main analysis and 0.93 after 2016, and for Chinese women, 0.85 in the main analysis and 0.91 after 2016. In the multivariable analysis, the hazard ratios for HRT prescriptions were 0.96, 0.94, and 0.92 for deprivation groups 3, 4, and 5 compared with group 1, respectively, in the main analysis, and 0.97, 0.95, and 0.94 for prescriptions after 2016. Higher prescribing rates in different parts of the UK were no longer significant in the East Midlands, East of England, West Midlands, or Yorkshire and Humber after 2016 (online supplemental table 10).

**Figure 2:**
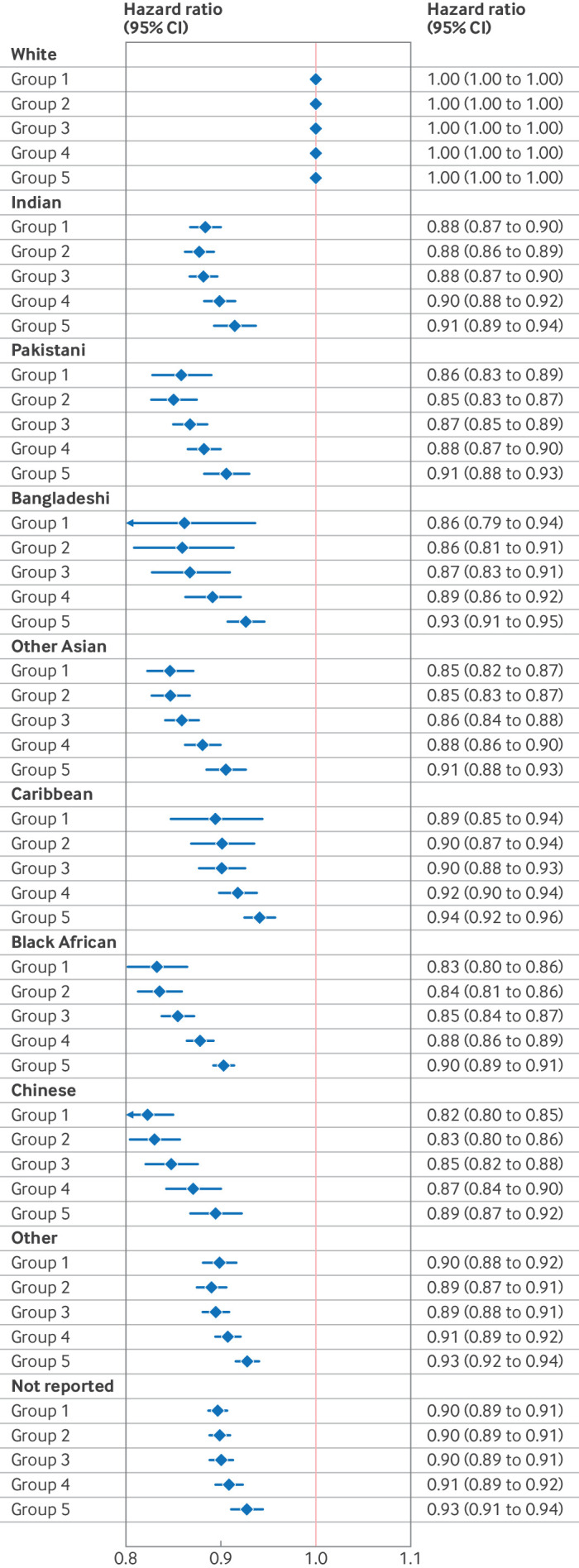
Forest plot showing adjusted hazard ratios and 95% confidence intervals (CIs) from multivariable Cox regression analysis of two or more hormone replacement therapy prescriptions, with an interaction between ethnic group and deprivation. Deprivation was measured by the Townsend deprivation score, divided into five equal groups, from group 1 (most affluent) to group 5 (most deprived). All hazard ratios were compared with white populations in the corresponding group

When an interaction term between ethnic group and Townsend deprivation score was included in the Cox regression analysis (online supplemental table 11 and figure 2), the trend towards lower HRT prescribing with increasing deprivation remained significant for every ethnic group individually, but the trend was no longer significant in Bangladeshi and black African women. A likelihood ratio test showed that inclusion of the interaction term significantly improved the model.

In the multinomial logistic regression models, women in all non-white ethnic groups were significantly less likely (P<0.001) to have one prescription or two or more prescriptions of oral oestrogen than white women. Oral oestrogen prescriptions (one or two or more) were lower in each social deprivation group than in the most affluent group for one or two or more prescriptions (online supplemental table 12). For transdermal oestrogens, prescriptions were lower for all ethnic groups for one or two or more prescriptions than for white populations and were lower in every social deprivation group compared with the most affluent group (online supplemental table 12). In the multilevel logistic regression model, inclusion of general practice as a random effect in the model suggested significant variability in HRT prescribing between general practices (P<0.001), and explained 2.9% of the variability in HRT prescribing, but did not substantially change the results.

In multivariable logistic regression, evaluating the likelihood of being prescribed transdermal compared with oral oestrogen, we found no differences by ethnic group (online supplemental table 13). We saw, however, a lower odds of receiving transdermal oestrogen than oral oestrogen in the three most deprived groups compared with the most affluent group (odds ratio 0.77, 95% CI 0.68 to 0.87 in group 5). These patterns were similar for transdermal oestrogen-progestogen combination formulations compared with oral preparations (online supplemental table 13). We found a trend towards lower odds of being prescribed transdermal HRT in all regions of England compared with London, but for many regions this trend was not significant. Restricting these analyses to prescriptions after 1 January 2016, we found that the trends for lower prescribing in the most deprived groups remained but were no longer significant for oestrogen or combination formulations (online supplemental table 13).

Table 3 shows the proportions of women with comorbidities recorded before starting HRT, or at study entry in those not prescribed HRT, for the full population and grouped by whether they received two or more HRT prescriptions. The proportions with each condition individually were similar in the population prescribed HRT and in those not prescribed HRT for most conditions, with a few exceptions. More than 25% of those with these conditions were prescribed HRT: malabsorption (29.3%), arrhythmia (27.6%), osteoporosis (25.5%), attention deficit hyperactivity disorder (50.9%), autism (31.2%), and rheumatoid arthritis or systemic lupus erythematosus (26.0%). Fewer than 10% of women were prescribed HRT if they had sickle cell disease (6.0%), sickle cell trait (7.5%), breast cancer (2.5%), a learning disability (8.5%), or Down's syndrome (7.0%).

**Table 3:** Cohort characteristics and comorbidities at baseline by use of hormone replacement therapy (HRT, two or more prescriptions) and unadjusted hazard ratios for two or more HRT prescriptions by comorbidity

Comorbidities	Descriptive characteristics			Cox regression	
	No HRT	Any HRT formulation	Total No of women	Unadjusted hazard ratio (95% CI)	P value
Total No of comorbidities	1 598 437	379 911	1 978 348	—	—
Cardiovascular					
Cardiovascular disease	19 904 (83.8)	3837 (16.2)	23 741 (1.2)	0.97 (0.96 to 0.98)	<0.001
Coronary heart disease	9176 (83.9)	1764 (16.1)	10 940 (0.6)	0.97 (0.95 to 0.99)	0.001
Stroke	11 408 (84.2)	2142 (15.8)	13 550 (0.7)	0.97 (0.95 to 0.98)	<0.001
Deep vein thrombosis	13 564 (84.7)	2457 (15.3)	16 021 (0.8)	0.96 (0.94 to 0.97)	<0.001
Cerebral venous thrombosis	176 (87.1)	26 (12.9)	202 (0.0)	0.93 (0.81 to 1.07)	0.341
Disseminated intravascular coagulation	72 (85.7)	12 (14.3)	84 (0.0)	0.94 (0.76 to 1.16)	0.561
Arterial thrombosis	3946 (86.9)	596 (13.1)	4542 (0.2)	0.94 (0.91 to 0.97)	<0.001
Myocardial infarction	3530 (87.5)	506 (12.5)	4036 (0.2)	0.94 (0.91 to 0.96)	<0.001
Stroke or transient ischaemic attack	8593 (86.7)	1322 (13.3)	9915 (0.5)	0.94 (0.92 to 0.96)	<0.001
Venous thromboembolism	23 116 (82.7)	4840 (17.3)	27 956 (1.4)	0.98 (0.97 to 0.99)	<0.001
Coronary artery bypass graft	582 (89.3)	70 (10.7)	652 (0.0)	0.92 (0.85 to 0.99)	0.029
Ischaemic stroke	10 883 (84.1)	2054 (15.9)	12 937 (0.7)	0.97 (0.95 to 0.98)	<0.001
Haemorrhagic stroke	773 (87.7)	108 (12.3)	881 (0.0)	0.93 (0.87 to 1.00)	0.037
Arrhythmia	17 672 (72.4%)	6753 (27.6)	24 425 (1.2)	1.11 (1.09 to 1.12)	<0.001
Myocarditis	397 (76.3%)	123 (23.7)	520 (0.0)	1.05 (0.96 to 1.14)	0.272
Valvular myocarditis	3259 (75.8)	1042 (24.2)	4301 (0.2)	1.06 (1.02 to 1.09)	<0.001
Hypertension	145 041 (80.8)	34 554 (19.2)	179 595 (9.1)	1.00 (1.00 to 1.01)	0.291
Type 1 diabetes	6711 (79.8)	1704 (20.2)	8415 (0.4)	1.01 (0.99 to 1.03)	0.368
Type 2 diabetes	54 630 (84.5)	10 023 (15.5)	64 653 (3.3)	0.96 (0.95 to 0.97)	<0.001
Renal disease	15 105 (80.6)	3643 (19.4)	18 748 (0.9)	1.00 (0.99 to 1.02)	0.764
Other					
Osteoporosis	8861 (74.5)	3034 (25.5)	11 895 (0.6)	1.07 (1.05 to 1.09)	<0.001
Malabsorption conditions	20 452 (70.7)	8460 (29.3)	28 912 (1.5)	1.13 (1.11 to 1.14)	<0.001
Endocrine conditions	15 380 (76.0)	4862 (24.0)	20 242 (1.0)	1.06 (1.04 to 1.07)	<0.001
Sickle cell disease	746 (94.0)	48 (6.0)	794 (0.0)	0.88 (0.82 to 0.94)	<0.001
Sickle cell trait	6001 (92.5)	488 (7.5)	6489 (0.3)	0.89 (0.87 to 0.91)	<0.001
Thalassaemia	5256 (87.9)	726 (12.1)	5982 (0.3)	0.93 (0.91 to 0.95)	<0.001
Any cancer diagnoses	41 248 (82.5)	8778 (17.5)	50 026 (2.5)	0.97 (0.96 to 0.98)	<0.001
Breast cancer	22 485 (97.5)	577 (2.5)	23 062 (1.2)	0.85 (0.84 to 0.86)	<0.001
Cirrhosis	1886 (81.8)	420 (18.2)	2306 (0.1)	0.99 (0.95 to 1.04)	0.787
Serious mental illness	42 870 (75.2)	14 161 (24.8)	57 031 (2.9)	1.07 (1.06 to 1.08)	<0.001
Attention deficit hyperactivity disorder	493 (49.1)	512 (50.9)	1005 (0.1)	1.55 (1.46 to 1.65)	<0.001
Homelessness	1102 (82.7)	230 (17.3)	1332 (0.1)	0.98 (0.93 to 1.04)	0.493
Learning disability	6442 (91.5)	598 (8.5)	7040 (0.4)	0.90 (0.87 to 0.92)	<0.001
Down's syndrome	1070 (93.0)	81 (7.0)	1151 (0.1)	0.88 (0.83 to 0.93)	<0.001
Dementia	732 (89.4)	87 (10.6)	819 (0.0)	0.92 (0.86 to 0.98)	0.015
Autism	708 (68.8)	321 (31.2)	1029 (0.1)	1.15 (1.09 to 1.23)	0.001
Parkinson's disease	618 (79.7)	157 (20.3)	775 (0.0)	1.01 (0.94 to 1.08)	0.768
Epilepsy	20 872 (80.5)	5051 (19.5)	25 923 (1.3)	1.00 (0.99 to 1.01)	0.763
Motor neuron disease	94 (80.3)	23 (19.7)	117 (0.0)	1.01 (0.84 to 1.21)	0.925
Multiple sclerosis	6425 (77.5)	1865 (22.5)	8290 (0.4)	1.04 (1.02 to 1.06)	<0.001
Asthma or chronic obstructive pulmonary disease	184 344 (75.7)	59 187 (24.3)	243 531 (12.3)	1.07 (1.06 to 1.07)	<0.001
Rheumatoid arthritis or systemic lupus erythematosus	29 130 (74.0)	10 219 (26.0)	39 349 (2.0)	1.08 (1.07 to 1.09)	<0.001
Body mass index					
Underweight (<18.5)	18 547 (81.2)	4282 (18.8)	22 829 (1.8)	0.95 (0.94 to 0.96)	<0.001
Normal weight (18.5-24.9)	396 395 (77.2)	117 371 (22.8)	513 766 (39.6)	1.00	—
Overweight (25-29.9)	302 416 (77.7)	86 659 (22.3)	389 075 (30)	0.99 (0.99 to 1.00)	<0.001
Obese (≥30)	300 525 (81.1)	70 127 (18.9)	370 652 (28.6)	0.95 (0.95 to 0.96)	<0.001

Data for descriptive characteristics are number (%).

CI, confidence interval.

Table 3 shows the results of the univariable Cox regression model for HRT prescriptions in those with different health conditions. Women who had a diagnosis of coronary heart disease, cardiovascular disease, stroke, deep vein thrombosis, arterial thrombosis, myocardial infarction, stroke or transient ischaemic attack, venous thromboembolism, ischaemic stroke, type 2 diabetes, sickle cell disease, sickle cell trait, thalassaemia, any cancer, breast cancer, learning disabilities, and Down's syndrome had significantly lower rates of receiving a HRT prescription after they had received their diagnosis than those without the condition, with hazard ratios varying from 0.85 (95% CI 0.84 to 0.86) for those with a diagnosis of breast cancer to 0.98 (0.97 to 0.99) for those with venous thromboembolism. Women with arrhythmia, valvular myocarditis, osteoporosis, malabsorption conditions, endocrine conditions, serious mental illness, attention deficit hyperactivity disorder, autism, multiple sclerosis, asthma or chronic obstructive pulmonary disease, and rheumatoid arthritis or systemic lupus erythematosus had significantly higher rates of HRT prescribing than those without the condition in univariable Cox regression models, with hazard ratios varying from 1.04 (95% CI 1.02 to 1.06) for those with a diagnosis of multiple sclerosis to 1.55 (1.46 to 1.65) for those with attention deficit hyperactivity disorder. Women with underweight, overweight, or obesity at the start of follow-up were significantly less likely to receive a HRT prescription than those with normal weight (hazard ratio 0.95, 95% CI 0.94 to 0.96 for underweight; 0.99, 0.99 to 1.00 for overweight; and 0.95, 0.95 to 0.96 for obesity).

Online supplemental table 14 shows Cox regression results for any HRT prescribing after adjusting for body mass index, smoking status, and existing cardiovascular disease. Adjusting for these factors did not explain the differences in prescribing, with hazard ratios for Chinese and other Asian ethnic groups of 0.83 (0.81 to 0.85) and 0.84 (0.83 to 0.85), respectively, and a hazard ratio of 0.92 (0.92 to 0.93) for the most deprived compared with the most affluent group.

## Discussion

### Principal findings

In this analysis of almost two million women aged 40-60 years in 2013-23 in the UK, we found that 19.2% of women received at least two HRT prescriptions, but this value varied from 3.9% of women from the black African ethnic group to 22.6% in white women. HRT prescribing rates for women living in the most affluent neighbourhoods were more than double those in the most deprived neighbourhoods (24.2% *v* 10.9%, respectively), a pattern that remained consistent across each ethnic group. These patterns in prescribing were seen for all HRT formulations. Regression analyses confirmed significantly lower proportions of HRT prescribing from age 40 years for women from all ethnic groups than for white women, after adjusting for deprivation and region, with adjusted hazard ratios ranging from 0.85 in Chinese women to 0.92 in Caribbean women during follow-up.

The likelihood of receiving two or more HRT prescriptions from age 40 years was significantly lower for each increase in social deprivation group, with a hazard ratio of 0.92 for the most deprived (group 5) compared with the most affluent (group 1) group during follow-up. Women in the most deprived areas were less likely to receive transdermal preparations than those in the most affluent areas, for both oestrogen prescriptions (odds ratio 0.77, 95% CI 0.68 to 0.87) and combined oestrogen-progestogen formulations (odds ratio 0.89, 0.82 to 0.96). HRT prescribing rates were significantly higher in all regions of England than in London, after adjusting for ethnic group and social deprivation. These patterns in prescribing were seen for all types of HRT and routes of administration examined. The trends in prescribing by ethnic group and social deprivation remained after publication of the NICE menopause guideline in 2015,^8^ but the hazard ratios suggested that these differences were smaller. This finding indicates that differences in HRT prescribing by ethnic group, region of England, and levels of deprivation might be improving over time, but formal testing has not been done.

### Strengths and limitations of this study

In this study, we quantified ethnic differences in HRT prescribing at an individual level for nine ethnic groups in English primary care data. We used data from a large cohort of women with robust data on self-assigned ethnic group, social deprivation, and region of England, so that we could quantify differences in HRT prescribing across different sociodemographic characteristics. By using electronic health records of HRT prescriptions and dates from general practice records, we could accurately determine the number and timing of prescriptions for each individual. Our study used several different statistical methods to analyse the data and we specifically looked at recent HRT prescribing to determine whether publication of the NICE guidance in 2015 has changed prescribing patterns.^8^ Also, we described the proportion of women with comorbidities or conditions that might have affected their willingness to take HRT or their likelihood of being prescribed HRT, in those groups of women who were prescribed and those who were not prescribed HRT. Our results showed that a number of health conditions were associated with lower or higher rates of HRT prescribing, which has not previously been reported for such a wide range of health conditions.

Our study had some limitations. Having a HRT prescription does not necessarily mean that an individual received their drug treatment or took it appropriately. To mitigate against this limitation, we used data only from women who received two or more prescriptions for the same type of drug treatment in our primary analyses as an indicator of likely use. We also reported the proportion of women who received at least one prescription and three or more prescriptions of HRT as well as the characteristics of those who had longer follow-up times to capture more women who might have had menopausal symptoms. Trends in prescribing were similar in all groups but our data did not include prescriptions from specialist secondary care or private settings. Inclusion of private prescription data, however, could exacerbate the trends seen in socioeconomic status.

We did not have data on intrauterine devices fitted in secondary care, but we included oestrogen only prescriptions and therefore these would be included under any HRT prescriptions. Although ethnic group was recorded based on self-identified ethnic group and was complete for >94% of women, many ethnic groups or different cultural groups exist that were not included within the nine groups in the QResearch database (eg, Eastern European populations living in England). In our analysis, missing data on ethnic group and social deprivation were included as a missing category.

Our study was informed and supported by a diverse patient group who helped us identify concerns and ensured our work was relevant to women. The Townsend deprivation index is a widely used measure of material deprivation based on four variables, including car ownership. Car ownership may not be the best measure of deprivation, however, particularly in urban areas, not owning a car might not always accurately reflect actual deprivation, which is a limitation of our analyses.

### Comparison with other studies

Previous reports of HRT prescribing in different ethnic groups in the US^33^ found significantly lower prescribing rates in African American, Asian, Latina, and Soviet immigrants than in white women (P<0.01), between 1992 and 1995. We also found marked differences in HRT prescribing, despite free NHS care, suggesting non-financial factors also contribute to these differences.

In the UK, a 1999 survey in an ethnically diverse population in South London reported that women from Caribbean, West African, or South Asian ethnic groups were less likely to be taking HRT than their white counterparts.^24^ In 2001, an analysis of 15 256 women aged 40-69 years from Health Survey for England^34^ also found that women from South Asian and black ethnic groups were significantly less likely to be users of HRT than white women (unadjusted odds ratio 0.56 and 0.45, P<0.05, respectively), but these differences were no longer significant when adjusted for sociodemographic factors. Our study showed that more than 20 years later and in the nine years after publication of the first NICE menopause guidance,^8^ this pattern in prescribing remained across England, even after adjusting for other sociodemographic factors. We reported adjusted hazard ratios for HRT prescriptions for South Asian women (Indian, Bangladeshi, or Pakistani) that ranged from 0.87 to 0.90 for the full dataset, and from 0.93 to 0.94 for prescriptions since 2016, and in black women (black African and Caribbean) from 0.88 to 0.92 in the full dataset, and from 0.93 to 0.95 for HRT prescriptions since 2016. These patterns were similar for all ethnic groups, suggesting that the gap in HRT prescribing might be decreasing.

In many countries, greater use of HRT has been reported to be associated with higher education status, higher income, and greater affluence.^35^,^38^ Similar patterns were seen in UK data for a range of measures of socioeconomic status, including social class, employment, and home ownership, but only social class remained significant after adjustment for other factors.^34^ Our study, based on UK data, also found that HRT prescriptions were significantly higher in the most affluent group than in all other social deprivation groups, measured with the Townsend index of deprivation. This trend remained significant after adjusting for other sociodemographic factors. Our work builds on the results of Hillman et al^23^ who found that HRT prescribing rates in general practices in the most deprived parts of the UK were lower than those in the most affluent areas. We have confirmed this finding in our analysis and identified individual factors associated with HRT prescribing. Also, by including an interaction term between social deprivation group and ethnic group in our analysis, we established that the trend towards lower prescribing in more deprived groups remained for all ethnic groups. This result suggests that socioeconomic status is an important determinant of HRT prescribing, regardless of ethnic group, which is consistent with other reports of UK data.^23 39^

When we examined prescription of individual HRT formulations, with oral and transdermal oestrogen prescriptions as example formulations, we found similar trends for transdermal oestrogen prescribing, but the trend for fewer prescriptions with increasing social deprivation was no longer significant for oral oestrogen prescriptions. When comparing prescriptions of transdermal with oral preparations, however, we found a significant trend towards lower rates of prescribing with increasing deprivation for both oestrogens and progestogens. Although lower prescribing of transdermal preparations in the most deprived group was no longer significant after publication of NICE guidance,^8^ a trend remained. This pattern, which has previously been found in an analysis of UK prescribing data,^23^ is unexpected because women in areas of high social deprivation have higher rates of cardiovascular disease^25^ and transdermal HRT preparations are recommended for those with a greater risk of cardiovascular disease.^40 41^ Qualitative studies with prescribers might help to determine why there are fewer prescriptions for transdermal HRT preparations in this group. In sensitivity analyses adjusting for smoking status, body mass index, and cardiovascular comorbidities, we found no difference in patterns of prescribing of any HRT.

Our analysis showed that those with breast cancer and sickle cell disease had the lowest rates of HRT prescribing. HRT is associated with an increased risk of breast cancer, which varies by type and length of use of HRT.^40 41^ HRT is not recommended in women with existing or a history of breast cancer,^42 43^ and so lower prescribing in those with breast cancer in our study is expected. For those with sickle cell disease, little evidence exists about how HRT might affect health outcomes.^44 45^ Women with sickle cell disease have a higher risk of blood clots^46^ (an increased risk with oral oestrogen) but the disease can adversely affect bone density,^47 48^ which would be improved with the use of HRT. Furthermore, sickle cell disease occurs almost exclusively in ethnic groups that already have the lowest prescribing rates and for whom mistrust in healthcare has been reported.^49^

We found that those with attention deficit hyperactivity disorder, malabsorption conditions, and osteoporosis had the highest rates of HRT prescribing. HRT can be beneficial for those with osteoporosis.^8^ Only 0.1% of the cohort had a record of attention deficit hyperactivity disorder and >50% had prescriptions for HRT. Why those with attention deficit hyperactivity disorder had higher prescribing rates is unclear, but menopause could exacerbate symptoms,^50^ or these groups might already have active engagement with healthcare.

Some lifestyle factors and health conditions that are most prevalent in areas of high social deprivation, such as diabetes,^16^ obesity,^51^ and smoking,^52 53^ are associated with severe symptoms of menopause. In our age adjusted analysis, however, we showed that those with diabetes and with overweight or obesity were significantly less likely to receive a prescription for HRT than those without diabetes or with normal weight, which might reflect cautious prescribing, as recommended by NICE guidance.^43^

Although the average length of menopausal symptoms is seven years,^1^ this varies by population characteristics. Those with a lower level of education have been reported to have a longer length of symptoms than those with a higher level of education.^1^ Ethnic group is also associated with length of symptoms, with Hispanic and Chinese women reporting the shortest length of symptoms (median <6 years) and African American women the longest (median >10 years).^1^ The SWAN study found a higher burden and length of symptoms, including vasomotor symptoms (hot flashes and night sweats), clinically significant depressive symptoms, and lower quality of life in black than in white women.^13^ Some evidence suggests that the types of symptoms that affect women differ between racial groups,^54^ which might not always be considered. Our work has established that black women are less likely to receive HRT than white women, but mean length of use of oestrogen formulations in Caribbean women was higher than in other ethnic groups, which suggests that the long length of symptoms in this UK population might be consistent with US data in African American women.^1^

We also established that a proportion of women who receive one prescription of HRT do not then receive a second prescription. The proportion of women who received only one prescription relative to those who received a second prescription was higher in women from all ethnic groups other than white women. We do not know the reasons for this difference without further investigation, but these women might have changed their mind about taking HRT, or wanted to accept the prescription to please their doctor, or they tried HRT and did not find it helpful enough. These data indicate differences in populations with symptoms of menopause and the proportion that then receives HRT.

We found that the average age of the first HRT prescription was 49 years. As the average age of menopause in the UK is 51 years,^55^ this finding could suggest that more women are being prescribed HRT in the menopause transition or perimenopause period, even though limited trial evidence exists on the efficacy of HRT in this group. Alternatively, women of a younger age at menopause might be more likely to be prescribed HRT. We also found that HRT prescribing was higher in every region of England compared with London, consistent with findings from other research in the UK.^34^ This effect remained even after adjusting for age and ethnic group in our analysis to account for the population living in London being more ethnically diverse than other regions of England and having a slightly lower mean age.

### Study implications for clinicians and policy makers

In this study, we identified, described, and quantified differences in HRT prescribing by individual characteristics based on UK primary care data. Whether women who received HRT were those with the most severe symptoms of menopause is unclear, as is whether comorbidities or preferences against the prescribing of HRT exist in some of these groups. One explanation for the lower rates of prescribing in some groups could be lack of awareness, difficulty in describing menopause and symptoms, or not receiving care from a female practitioner for women from some ethnic groups.^56^ Clinicians responsible for prescribing HRT must ensure that women with problematic or severe menopausal symptoms have the same access to care, despite social deprivation or ethnic group. Understanding women's preferences and what interventions are required to deal with the inequities in menopause care is complex and outside the scope of this project; further exploration with qualitative methodologies is needed. Developing knowledge and understanding about how menopausal experiences vary across different countries, cultures, and ethnic backgrounds worldwide was identified as a top 10 priority by the James Lind Alliance Priority Setting Partnership.^57^ Women’s experiences of menopause and accessing care, as well as how perceptions and approaches might be shaped by cultural norms, could be explored with in-depth semi-structured interviews and focus groups of women with symptoms of menopause and the healthcare professionals caring for them.

### Future research

Further research should determine if women with the most severe symptoms are those receiving HRT or if some women who need HRT are not receiving this treatment, leaving an unmet clinical need. Understanding more about choices made by women and potential preferences for non-hormonal treatments is important. Also, understanding whether women need more information about managing the symptoms of menopause, and how clinicians navigate consultations about menopause and treatments including HRT.

### Conclusions

We have identified differences in HRT prescribing in England based on ethnic group, socioeconomic status, and geographical location. White women and those in more affluent neighbourhoods were more likely to receive HRT than non-white women and those in more deprived areas. These findings suggest potential inequities that require further study.

## Supplementary material

10.1136/bmjmed-2025-001349 online supplemental file 1

10.1136/bmjmed-2025-001349 online supplemental figure 1

10.1136/bmjmed-2025-001349 online supplemental figure 2

## Data Availability

No data are available.
