# Supplementary material for: Inequalities in hormone replacement therapy prescribing in UK primary care: population based cohort study
Source: BMJ Med. 2025 Sep 25;4(1):e001349. doi: 10.1136/bmjmed-2025-001349 (PMC12481343; doi:10.1136/bmjmed-2025-001349)
Supplement: online supplemental file 1 [file bmjmed-4-1-s001.pdf]

Supplementary data

Supplementary Table 1 – comparison between ethnicity data in QResearch dataset with the ONS 2021 census(1) data in females aged 40-60.

Ethnicity	QResearch data	2021 census data(1)
White	1,506,788 (76.2%)	6,326,515 (75.9%)
Indian	60,142 (3.0%)	262,525 (3.1%)
Pakistani	33,909 (1.7%)	177,570 (2.1%)
Bangladeshi	16,579 (0.8%)	67,985 (0.8%)
Other Asian	38,279 (1.9%)	166,510 (2.0%)
Caribbean	32,951 (1.7%)	118,435 (1.4%)
Black African	67,320 (3.4%)	233,350 (2.8%)
Chinese	19,651 (1.0%)	69,235 (0.8%)
Other	87,175 (4.4%)	937,790 (11.2%)
Not recorded	115,554 (5.8%)	-

Supplementary Table 2 - Cohort characteristics of women **a.** aged 40-60 at study entry with a minimum of 5 years follow-up and **b.** age 45-60 at study entry with a minimum of 5 years follow-up, stratified by whether they received two or more prescriptions for HRT. Figures are numbers (column %).

	a. Population with > 5 years follow-up			b. Population age >45 at cohort entry with > 5 years follow-up		
	**No HRT**	**Any HRT formulation**	**Total**	**No HRT**	**Any HRT formulation**	**Total**
	(N=934,622)	(N=272,100)	(N=1,206,722)	(N=692,840)	(N=208,138)	(N=900,978)
Median (IQR) age at cohort entry	44(49, 54)	45(49, 54)	45(49, 54)	48(52, 56)	48(51, 56)	48(52, 56)
**Ethnicity N (column %)**						
White	706,021 (75.5%)	244,070 (89.7%)	950,091 (78.7%)	531,613 (76.7%)	186,714 (89.7%)	718,327 (79.7%)
Indian	30,906 (3.3%)	3,777 (1.4%)	34,683 (2.9%)	21,606 (3.1%)	2,717 (1.3%)	24,323 (2.7%)
Pakistani	18,753 (2.0%)	1,733 (0.6%)	20,486 (1.7%)	11,919 (1.7%)	1,228 (0.6%)	13,147 (1.5%)
Bangladeshi	9,290 (1.0%)	786 (0.3%)	10,076 (0.8%)	5,399 (0.8%)	503 (0.2%)	5,902 (0.7%)
Other Asian	18,109 (1.9%)	1,421 (0.5%)	19,530 (1.6%)	11,698 (1.7%)	1,027 (0.5%)	12,725 (1.4%)
Caribbean	19,947 (2.1%)	2,267 (0.8%)	22,214 (1.8%)	15,767 (2.3%)	1,941 (0.9%)	17,708 (2.0%)
Black African	31,449 (3.4%)	1,781 (0.7%)	33,230 (2.8%)	21,077 (3.0%)	1,291 (0.6%)	22,368 (2.5%)
Chinese	8,465 (0.9%)	563 (0.2%)	9,028 (0.7%)	5,909 (0.9%)	408 (0.2%)	6,317 (0.7%)
Other	37,346 (4.0%)	5,468 (2.0%)	42,814 (3.5%)	25,083 (3.6%)	4,027 (1.9%)	29,110 (3.2%)
Not recorded	54,336 (5.8%)	10,234 (3.8%)	64,570 (5.4%)	42,769 (6.2%)	8,282 (4.0%)	51,051 (5.7%)
**Quintile of Townsend deprivation N (column %)**						
1 – most affluent	264,632 (28.3%)	95,479 (35.1%)	360,111 (29.8%)	203,956 (29.4%)	72,919 (35.0%)	276,875 (30.7%)

2	216,822 (23.2%)	71,595 (26.3%)	288,417 (23.9%)	162,867 (23.5%)	54,601 (26.2%)	217,468 (24.1%)
3	176,994 (18.9%)	49,200 (18.1%)	226,194 (18.7%)	129,382 (18.7%)	37,665 (18.1%)	167,047 (18.5%)
4	146,043 (15.6%)	33,216 (12.2%)	179,259 (14.9%)	104,950 (15.1%)	25,622 (12.3%)	130,572 (14.5%)
5 – most deprived	125,826 (13.5%)	21,006 (7.7%)	146,832 (12.2%)	88,835 (12.8%)	16,192 (7.8%)	105,027 (11.7%)
Not recorded	4,305 (0.5%)	1,604 (0.6%)	5,909 (0.5%)	2,850 (0.4%)	1,139 (0.5%)	3,989 (0.4%)
**Region N (column %)**						
East Midlands	18,966 (2.0%)	6,080 (2.2%)	25,046 (2.1%)	14,207 (2.1%)	4,518 (2.2%)	18,725 (2.1%)
East of England	39,548 (4.2%)	12,844 (4.7%)	52,392 (4.3%)	29,478 (4.3%)	9,720 (4.7%)	39,198 (4.4%)
London	222,493 (23.8%)	38,319 (14.1%)	260,812 (21.6%)	157,192 (22.7%)	29,252 (14.1%)	186,444 (20.7%)
North East	26,147 (2.8%)	7,759 (2.9%)	33,906 (2.8%)	20,004 (2.9%)	6,044 (2.9%)	26,048 (2.9%)
North West	188,868 (20.2%)	63,829 (23.5%)	252,697 (20.9%)	142,827 (20.6%)	49,159 (23.6%)	191,986 (21.3%)
South Central	128,185 (13.7%)	43,234 (15.9%)	171,419 (14.2%)	95,090 (13.7%)	33,049 (15.9%)	128,139 (14.2%)
South East	101,854 (10.9%)	33,841 (12.4%)	135,695 (11.2%)	76,159 (11.0%)	26,196 (12.6%)	102,355 (11.4%)
South West	79,829 (8.5%)	27,884 (10.2%)	107,713 (8.9%)	60,910 (8.8%)	20,891 (10.0%)	81,801 (9.1%)
West Midlands	100,397 (10.7%)	29,046 (10.7%)	129,443 (10.7%)	75,613 (10.9%)	22,308 (10.7%)	97,921 (10.9%)
Yorkshire & Humber	28,335 (3.0%)	9,264 (3.4%)	37,599 (3.1%)	21,360 (3.1%)	7,001 (3.4%)	28,361 (3.1%)

Supplementary Table 3 – Two or more prescriptions of different HRT types and modes of administration by ethnicity. Data are presented as numbers and column percent unless otherwise specified

**Ethnicity**	**White** (N=1,506,788)	**Indian** (N=60,142)	**Pakistani** (N=33,909)	**Bangladeshi** (N=16,579)	**Other Asian** (N=38,279)	**Caribbean** (N=32,951)	**Black African** (N=67,320)	**Chinese** (N=19,651)	**Other** (N=87,175)	**Not reported** (N=115,554)
Mean(SD) age at cohort entry	49.6 (5.9)	48.5 (6.1)	48.0 (6.0)	47.2 (6.0)	48.0 (5.8)	50.0 (5.6)	48.0 (5.6)	48.1 (5.9)	48.1 (5.8)	49.7 (5.9)
Mean(SD) age at first HRT prescription	50.0 (5.4)	49.34 (5.34)	48.8 (5.5)	47.6 (5.4)	49.2 (5.2)	50.3 (5.9)	48.3 (5.8)	49.8 (5.3)	49.9 (5.2)	50.2 (5.4)
**Quintile of Townsend**										

**deprivation, N (col%)**										
1 – most affluent	467,910 (31.1%)	11,955 (19.9%)	2,970 (8.8%)	564 (3.4%)	4,766 (12.5%)	1,336 (4.1%)	2,755 (4.1%)	3,730 (19.0%)	10,322 (11.8%)	34,198 (29.6%)
2	384,593 (25.5%)	13,295 (22.1%)	4,910 (14.5%)	1,044 (6.3%)	7,000 (18.3%)	2,875 (8.7%)	5,127 (7.6%)	3,860 (19.6%)	13,157 (15.1%)	28,687 (24.8%)
3	286,507 (19.0%)	14,958 (24.9%)	9,362 (27.6%)	1,737 (10.5%)	9,365 (24.5%)	5,424 (16.5%)	9,995 (14.8%)	3,708 (18.9%)	16,830 (19.3%)	22,296 (19.3%)
4	212,985 (14.1%)	12,412 (20.6%)	10,662 (31.4%)	3,669 (22.1%)	8,917 (23.3%)	8,640 (26.2%)	16,179 (24.0%)	3,582 (18.2%)	19,784 (22.7%)	16,641 (14.4%)
5 – most deprived	143,088 (9.5%)	7,020 (11.7%)	5,844 (17.2%)	9,464 (57.1%)	7,812 (20.4%)	14,486 (44.0%)	32,498 (48.3%)	4,335 (22.1%)	26,103 (29.9%)	13,000 (11.3%)
Missing	11,705 (0.8%)	502 (0.8%)	161 (0.5%)	101 (0.6%)	419 (1.1%)	190 (0.6%)	766 (1.1%)	436 (2.2%)	979 (1.1%)	732 (0.6%)
**HRT prescriptions, N (col%)** Any type of HRT	340,434 (22.6%)	5,213 (8.7%)	2,305 (6.8%)	1,021 (6.2%)	2,061 (5.4%)	2,940 (8.9%)	2,650 (3.9%)	819 (4.2%)	8,038 (9.2%)	14,430 (12.5%)
**Any oestrogen**	174,628 (11.6%)	2,545 (4.2%)	934 (2.8%)	301 (1.8%)	915 (2.4%)	1,488 (4.5%)	1,021 (1.5%)	356 (1.8%)	3,818 (4.4%)	6,478 (5.6%)
Oral oestrogen	52,723 (3.5%)	594 (1.0%)	278 (0.8%)	97 (0.6%)	242 (0.6%)	454 (1.4%)	275 (0.4%)	88 (0.4%)	867 (1.0%)	1,766 (1.5%)
Transdermal oestrogen	137,267 (9.1%)	2,088 (3.5%)	689 (2.0%)	217 (1.3%)	706 (1.8%)	1,148 (3.5%)	790 (1.2%)	274 (1.4%)	3,196 (3.7%)	5,177 (4.5%)
Conjugated oestrogen	19,537 (1.3%)	242 (0.4%)	135 (0.4%)	43 (0.3%)	115 (0.3%)	228 (0.7%)	122 (0.2%)	31 (0.2%)	328 (0.4%)	694 (0.6%)
Topical vaginal oestrogen	74 (0.0%)	<5	<5	<5	<5	<5	<5	<5	<5	<5
Implant oestrogen	37 (0.0%)	<5	<5	<5	<5	<5	<5	<5	<5	<5
**Any oestrogen-progestogen**	211,137 (14.0%)	3,266 (5.4%)	1,590 (4.7%)	788 (4.8%)	1,330 (3.5%)	1,795 (5.4%)	1,844 (2.7%)	548 (2.8%)	5,238 (6.0%)	9,669 (8.4%)
Oral oestrogen-progestogen	147,360 (9.8%)	1,929 (3.2%)	1,099 (3.2%)	592 (3.6%)	845 (2.2%)	1,079 (3.3%)	1,182 (1.8%)	331 (1.7%)	3,192 (3.7%)	6,944 (6.0%)
Transdermal oestrogen-progestogen	88,531 (5.9%)	1,598 (2.7%)	617 (1.8%)	263 (1.6%)	600 (1.6%)	929 (2.8%)	798 (1.2%)	245 (1.2%)	2,602 (3.0%)	3,669 (3.2%)
**Any progestogen**	162,392 (10.8%)	3,342 (5.6%)	1,636 (4.8%)	565 (3.4%)	1,254 (3.3%)	1,378 (4.2%)	1,784 (2.7%)	477 (2.4%)	4,265 (4.9%)	6,983 (6.0%)
Oral progestogen	98,842 (6.6%)	2,084 (3.5%)	1,101 (3.2%)	382 (2.3%)	684 (1.8%)	798 (2.4%)	799 (1.2%)	257 (1.3%)	2,542 (2.9%)	4,172 (3.6%)
Vaginal progestogen	8,032 (0.5%)	296 (0.5%)	103 (0.3%)	42 (0.3%)	106 (0.3%)	97 (0.3%)	246 (0.4%)	43 (0.2%)	308 (0.4%)	340 (0.3%)
IUD progestogen	157,839 (10.5%)	3,036 (5.0%)	1,453 (4.3%)	464(2.8%)	1,486 (3.9%)	1,752 (5.3%)	2,767 (4.1%)	655 (3.3%)	4,750 (5.4%)	7,156 (6.2%)

Tibolone	3,558 (0.2%)	48 (0.1%)	41 (0.1%)	10 (0.1%)	22 (0.1%)	44 (0.1%)	37 (0.1%)	5 (0.0%)	97 (0.1%)	116 (0.1%)

Supplementary Table 4 – Prescriptions of different HRT types and modes of administration by ethnicity tabulated as those who received only a single prescription and those who received two or more prescriptions. Data are presented as numbers and column percent

**Ethnicity**	White (N=1,506,788)	Indian (N=60,142)	Pakistani (N=33,909)	Bangladeshi (N=16,579)	Other Asian (N=38,279)	Caribbean (N=32,951)	Black African (N=67,320)	Chinese (N=19,651)	Other (N=87,175)	Not reported (N=115,554)
Mean(SD) age at first HRT prescription	50.0 (5.4)	49.34 (5.34)	48.8 (5.5)	47.6 (5.4)	49.2 (5.2)	50.3 (5.9)	48.3 (5.8)	49.8 (5.3)	49.9 (5.2)	50.2 (5.4)
**HRT prescriptions, N (col%)**										
Any type of HRT	340,434 (22.6%)	5,213 (8.7%)	2,305 (6.8%)	1,021 (6.2%)	2,061 (5.4%)	2,940 (8.9%)	2,650 (3.9%)	819 (4.2%)	8,038 (9.2%)	14,430 (12.5%)
**Numbers of prescriptions:**										
**Any oestrogen**										
1	39,459 (2.6%)	952 (1.6%)	394 (1.2%)	151 (0.9%)	351 (0.9%)	477 (1.4%)	525 (0.8%)	114 (0.6%)	1,214 (1.4%)	1,616 (1.4%)
2	174,628 (11.6%)	2,545 (4.2%)	934 (2.8%)	301 (1.8%)	915 (2.4%)	1,488 (4.5%)	1,021 (1.5%)	356 (1.8%)	3,818 (4.4%)	6,478 (5.6%)
Oral oestrogen										
1	13,671 (0.9%)	294 (0.5%)	149 (0.4%)	48 (0.3%)	107 (0.3%)	191 (0.6%)	192 (0.3%)	31 (0.2%)	330 (0.4%)	530 (0.5%)
2	52,723 (3.5%)	594 (1.0%)	278 (0.8%)	97 (0.6%)	242 (0.6%)	454 (1.4%)	275 (0.4%)	88 (0.4%)	867 (1.0%)	1,766 (1.5%)
Transdermal oestrogen										
1	36,024 (2.4%)	793 (1.3%)	303 (0.9%)	116 (0.7%)	295 (0.8%)	392 (1.2%)	398 (0.6%)	98 (0.5%)	1,052 (1.2%)	1,368 (1.2%)
2	137,267 (9.1%)	2,088 (3.5%)	689 (2.0%)	217 (1.3%)	706 (1.8%)	1,148 (3.5%)	790 (1.2%)	274 (1.4%)	3,196 (3.7%)	5,177 (4.5%)

Conjugated oestrogen										
1	6,675 (0.4%)	121 (0.2%)	88 (0.3%)	25 (0.2%)	36 (0.1%)	96 (0.3%)	71 (0.1%)	11 (0.1%)	146 (0.2%)	245 (0.2%)
2	19,537 (1.3%)	242 (0.4%)	135 (0.4%)	43 (0.3%)	115 (0.3%)	228 (0.7%)	122 (0.2%)	31 (0.2%)	328 (0.4%)	694 (0.6%)
Topical vaginal oestrogen										
1	44 (0.0%)	3 (0.0%)	0 (0.0%)	0 (0.0%)	0 (0.0%)	0 (0.0%)	0 (0.0%)	0 (0.0%)	2 (0.0%)	2 (0.0%)
2	74 (0.0%)	0 (0.0%)	2 (0.0%)	0 (0.0%)	0 (0.0%)	0 (0.0%)	0 (0.0%)	0 (0.0%)	1 (0.0%)	4 (0.0%)
Implant oestrogen										
1	12 (0.0%)	0 (0.0%)	0 (0.0%)	0 (0.0%)	0 (0.0%)	0 (0.0%)	0 (0.0%)	0 (0.0%)	0 (0.0%)	1 (0.0%)
2	37 (0.0%)	1 (0.0%)	0 (0.0%)	0 (0.0%)	0 (0.0%)	0 (0.0%)	0 (0.0%)	0 (0.0%)	1 (0.0%)	2 (0.0%)
**Any oestrogen-progestogen**										
1	54,798 (3.6%)	1,487 (2.5%)	884 (2.6%)	373 (2.2%)	606 (1.6%)	737 (2.2%)	942 (1.4%)	232 (1.2%)	1,756 (2.0%)	2,529 (2.2%)
2	211,137 (14.0%)	3,266 (5.4%)	1,590 (4.7%)	788 (4.8%)	1,330 (3.5%)	1,795 (5.4%)	1,844 (2.7%)	548 (2.8%)	5,238 (6.0%)	9,669 (8.4%)
Oral oestrogen-progestogen										
1	44,907 (3.0%)	1,131 (1.9%)	707 (2.1%)	281 (1.7%)	439 (1.1%)	561 (1.7%)	686 (1.0%)	180 (0.9%)	1,324 (1.5%)	2,105 (1.8%)
2	147,360 (9.8%)	1,929 (3.2%)	1,099 (3.2%)	592 (3.6%)	845 (2.2%)	1,079 (3.3%)	1,182 (1.8%)	331 (1.7%)	3,192 (3.7%)	6,944 (6.0%)
Transdermal oestrogen-progestogen										
1	34,517 (2.3%)	809 (1.3%)	369 (1.1%)	165 (1.0%)	317 (0.8%)	406 (1.2%)	507 (0.8%)	121 (0.6%)	1,066 (1.2%)	1,422 (1.2%)
2	88,531 (5.9%)	1,598 (2.7%)	617 (1.8%)	263 (1.6%)	600 (1.6%)	929 (2.8%)	798 (1.2%)	245 (1.2%)	2,602 (3.0%)	3,669 (3.2%)
**Any progestogen**										
1	132,607 (8.8%)	3,982 (6.6%)	2,371 (7.0%)	922 (5.6%)	1,668 (4.4%)	1,912 (5.8%)	2,932 (4.4%)	671 (3.4%)	4,827 (5.5%)	6,299 (5.5%)
2	162,392 (10.8%)	3,342 (5.6%)	1,636 (4.8%)	565 (3.4%)	1,254 (3.3%)	1,378 (4.2%)	1,784 (2.7%)	477 (2.4%)	4,265 (4.9%)	6,983 (6.0%)
Oral progestogen										
1	54,460 (3.6%)	2,156 (3.6%)	1,546 (4.6%)	637 (3.8%)	714 (1.9%)	732 (2.2%)	874 (1.3%)	188 (1.0%)	1,868 (2.1%)	2,388 (2.1%)
2	98,842 (6.6%)	2,084 (3.5%)	1,101 (3.2%)	382 (2.3%)	684 (1.8%)	798 (2.4%)	799 (1.2%)	257 (1.3%)	2,542 (2.9%)	4,172 (3.6%)
Vaginal progestogen										

1	8,399 (0.6%)	304 (0.5%)	93 (0.3%)	40 (0.2%)	117 (0.3%)	125 (0.4%)	248 (0.4%)	72 (0.4%)	364 (0.4%)	383 (0.3%)
2	8,032 (0.5%)	296 (0.5%)	103 (0.3%)	42 (0.3%)	106 (0.3%)	97 (0.3%)	246 (0.4%)	43 (0.2%)	308 (0.4%)	340 (0.3%)
IUD progestogen										
1	101,853 (6.8%)	2,155 (3.6%)	1,075 (3.2%)	344 (2.1%)	1,063 (2.8%)	1,316 (4.0%)	2,070 (3.1%)	477 (2.4%)	3,385 (3.9%)	4,665 (4.0%)
2	55,986 (3.7%)	881 (1.5%)	378 (1.1%)	120 (0.7%)	423 (1.1%)	436 (1.3%)	697 (1.0%)	178 (0.9%)	1,365 (1.6%)	2,491 (2.2%)
Tibolone										
1	1,881 (0.1%)	41 (0.1%)	36 (0.1%)	13 (0.1%)	18 (0.0%)	37 (0.1%)	39 (0.1%)	9 (0.0%)	73 (0.1%)	75 (0.1%)
2	3,558 (0.2%)	48 (0.1%)	41 (0.1%)	10 (0.1%)	22 (0.1%)	44 (0.1%)	37 (0.1%)	5 (0.0%)	97 (0.1%)	116 (0.1%)

Supplementary Table 5 – Prescriptions of different HRT types and modes of administration by Quintile of Townsend deprivation where 1 is most affluent and 5 is most deprived. **Figures are numbers (column %) unless otherwise stated**

	Quintile of Townsend						
	1	2	3	4	5	Not reported	Total
N	540,506	464,548	380,182	313,471	263,650	15,991	1,978,348
Age at cohort entry, mean (SD)	49.9 (5.9)	49.6 (5.9)	49.2 (5.956)	49.0 (5.9)	48.6 (5.9)	48.4 (6.0)	49.4 (6.0)
Age at first HRT prescription, mean (SD)	50.2 (5.2)	50.0 (5.4)	49.4 (5.5)	49.1 (5.5)	49.0 (5.5)	49.6 (5.2)	49.8 (5.4)
Any type of HRT	130,677 (24.2%)	101,593 (21.9%)	69,378 (18.2%)	46,218 (14.7%)	28,781 (10.9%)	3,264 (20.4%)	379,911 (19.2%)
**Prescriptions:**							
**Any oestrogen**	69,555 (12.9%)	52,855 (11.4%)	34,382 (9.0%)	21,611 (6.9%)	12,210 (4.6%)	1,871 (11.7%)	192,484 (9.7%)

Oral oestrogen	18,437 (3.4%)	15,065 (3.2%)	11,280 (3.0%)	7,813 (2.5%)	4,362 (1.7%)	427 (2.7%)	57,384 (2.9%)
Transdermal oestrogen	56,622 (10.5%)	42,141 (9.1%)	26,316 (6.9%)	15,951 (5.1%)	8,938 (3.4%)	1,584 (9.9%)	151,552 (7.7%)
Conjugated oestrogen	6,288 (1.2%)	5,253 (1.1%)	4,396 (1.2%)	3,381 (1.1%)	2,023 (0.8%)	134 (0.8%)	21,475 (1.1%)
Topical vaginal oestrogen	27 (0.0%)	21 (0.0%)	13 (0.0%)	14 (0.0%)	6 (0.0%)	<5	81 (0.0%)
implant oestrogens	19 (0.0%)	9 (0.0%)	9 (0.0%)	<5	<5	<5	41 (0.0%)
**Any oestrogen-progestogen**	79,729 (14.8%)	62,484 (13.5%)	43,593 (11.5%)	29,760 (9.5%)	19,742 (7.5%)	1,897 (11.9%)	237,205 (12.0%)
Oral oestrogen-progestogen	54,192 (10.0%)	43,123 (9.3%)	30,521 (8.0%)	21,421 (6.8%)	14,098 (5.3%)	1,198 (7.5%)	164,553 (8.3%)
Transdermal oestrogen-progestogen	34,942 (6.5%)	26,588 (5.7%)	17,990 (4.7%)	11,613 (3.7%)	7,799 (3.0%)	920 (5.8%)	99,852 (5.0%)
**Any progestogen**	69,831 (12.9%)	51,650 (11.1%)	32,110 (8.4%)	18,702 (6.0%)	10,023 (3.8%)	1,760 (11.0%)	184,076 (9.3%)
Oral progestogen	42,059 (7.8%)	30,696 (6.6%)	19,379 (5.1%)	11,713 (3.7%)	6,669 (2.5%)	1,145 (7.2%)	111,661 (5.6%)
Vaginal progestogen	3,578 (0.7%)	2,606 (0.6%)	1,604 (0.4%)	1,099 (0.4%)	652 (0.2%)	74 (0.5%)	9,613 (0.5%)
IUD progestogen	66,331 (12.3%)	51,410 (11.1%)	33,004 (8.7%)	19,054 (6.1%)	9,928 (3.8%)	1,631 (10.2%)	181,358 (9.2%)
Tibolone	1,308 (0.2%)	1,031 (0.2%)	755 (0.2%)	560 (0.2%)	304 (0.1%)	20 (0.1%)	3,978 (0.2%)

Supplementary Table 6 – Prescriptions of different HRT types and modes of administration by Quintile of Townsend deprivation where 1 is most affluent and 5 is most deprived tabulated as those who received only a single prescription and those who received

two or more prescriptions.. Data are presented as numbers and column percent.

	Quintile of Townsend						
	1	2	3	4	5	Not reported	Total
N	540,506	464,548	380,182	313,471	263,650	15,991	1,978,348
Age at first HRT prescription, mean (SD)	50.2 (5.2)	50.0 (5.4)	49.4 (5.5)	49.1 (5.5)	49.0 (5.5)	49.6 (5.2)	49.8 (5.4)
Any type of HRT	130,677 (24.2%)	101,593 (21.9%)	69,378 (18.2%)	46,218 (14.7%)	28,781 (10.9%)	3,264 (20.4%)	379,911 (19.2%)
**Numbers of prescriptions:**							
**Any oestrogen**							
1	14,997 (2.8%)	12,109 (2.6%)	8,406 (2.2%)	5,711 (1.8%)	3,610 (1.4%)	420 (2.6%)	45,253 (2.3%)
2+	69,555 (12.9%)	52,855 (11.4%)	34,382 (9.0%)	21,611 (6.9%)	12,210 (4.6%)	1,871 (11.7%)	192,484 (9.7%)
Oral oestrogen							
1	4,691 (0.9%)	4,186 (0.9%)	3,056 (0.8%)	2,181 (0.7%)	1,326 (0.5%)	103 (0.6%)	15,543 (0.8%)
2+	18,437 (3.4%)	15,065 (3.2%)	11,280 (3.0%)	7,813 (2.5%)	4,362 (1.7%)	427 (2.7%)	57,384 (2.9%)
Transdermal oestrogen							
1	13,926 (2.6%)	10,913 (2.3%)	7,558 (2.0%)	4,957 (1.6%)	3,069 (1.2%)	416 (2.6%)	40,839 (2.1%)
2+	56,622 (10.5%)	42,141 (9.1%)	26,316 (6.9%)	15,951 (5.1%)	8,938 (3.4%)	1,584 (9.9%)	151,552 (7.7%)
Conjugated oestrogen							
1	2,056 (0.4%)	1,906 (0.4%)	1,583 (0.4%)	1,177 (0.4%)	743 (0.3%)	49 (0.3%)	7,514 (0.4%)
2+	6,288 (1.2%)	5,253 (1.1%)	4,396 (1.2%)	3,381 (1.1%)	2,023 (0.8%)	134 (0.8%)	21,475 (1.1%)

Topical vaginal oestrogen							
1	8 (0.0%)	14 (0.0%)	16 (0.0%)	<5	8 (0.0%)	<5	51 (0.0%)
2+	27 (0.0%)	21 (0.0%)	13 (0.0%)	14 (0.0%)	6 (0.0%)	<5	81 (0.0%)
Implant							
1	6 (0.0%)	<5	<5	<5	<5	<5	13 (0.0%)
2+	19 (0.0%)	9 (0.0%)	9 (0.0%)	<5	<5	<5	41 (0.0%)
**Any oestrogen-progestogen**							
1	19,707 (3.6%)	16,092 (3.5%)	12,389 (3.3%)	9,014 (2.9%)	6,618 (2.5%)	524 (3.3%)	64,344 (3.3%)
2+	79,729 (14.8%)	62,484 (13.5%)	43,593 (11.5%)	29,760 (9.5%)	19,742 (7.5%)	1,897 (11.9%)	237,205 (12.0%)
Oral oestrogen-progestogen							
1	15,911 (2.9%)	13,148 (2.8%)	10,044 (2.6%)	7,449 (2.4%)	5,369 (2.0%)	400 (2.5%)	52,321 (2.6%)
2+	54,192 (10.0%)	43,123 (9.3%)	30,521 (8.0%)	21,421 (6.8%)	14,098 (5.3%)	1,198 (7.5%)	164,553 (8.3%)
Transdermal oestrogen-progestogen							
1	12,966 (2.4%)	10,305 (2.2%)	7,350 (1.9%)	5,060 (1.6%)	3,640 (1.4%)	378 (2.4%)	39,699 (2.0%)
2+	34,942 (6.5%)	26,588 (5.7%)	17,990 (4.7%)	11,613 (3.7%)	7,799 (3.0%)	920 (5.8%)	99,852 (5.0%)
**Any progestogen**							
1	52,692 (9.7%)	42,285 (9.1%)	30,179 (7.9%)	19,800 (6.3%)	11,829 (4.5%)	1,406 (8.8%)	158,191 (8.0%)
2+	69,831 (12.9%)	51,650 (11.1%)	32,110 (8.4%)	18,702 (6.0%)	10,023 (3.8%)	1,760 (11.0%)	184,076 (9.3%)
Oral progestogen							
1	21,581 (4.0%)	17,000 (3.7%)	12,613 (3.3%)	8,624 (2.8%)	5,150 (2.0%)	595 (3.7%)	65,563 (3.3%)
2+	42,059 (7.8%)	30,696 (6.6%)	19,379 (5.1%)	11,713 (3.7%)	6,669 (2.5%)	1,145 (7.2%)	111,661 (5.6%)

Vaginal progestogen							
1	3,546 (0.7%)	2,674 (0.6%)	1,783 (0.5%)	1,172 (0.4%)	878 (0.3%)	92 (0.6%)	10,145 (0.5%)
2+	3,578 (0.7%)	2,606 (0.6%)	1,604 (0.4%)	1,099 (0.4%)	652 (0.2%)	74 (0.5%)	9,613 (0.5%)
IUD progestogen							
1	3,546 (0.7%)	2,674 (0.6%)	1,783 (0.5%)	1,172 (0.4%)	878 (0.3%)	92 (0.6%)	10,145 (0.5%)
2+	3,578 (0.7%)	2,606 (0.6%)	1,604 (0.4%)	1,099 (0.4%)	652 (0.2%)	74 (0.5%)	9,613 (0.5%)
Tibolone							
1	41,530 (7.7%)	33,025 (7.1%)	22,093 (5.8%)	13,353 (4.3%)	7,323 (2.8%)	1,079 (6.7%)	118,403 (6.0%)
2+	24,801 (4.6%)	18,385 (4.0%)	10,911 (2.9%)	5,701 (1.8%)	2,605 (1.0%)	552 (3.5%)	62,955 (3.2%)

Supplementary Table 7 – Duration of HRT use by ethnic group in women who had two or more HRT prescriptions (or 1+ prescriptions of IUD progestogen)

	Ethnicity										
	White	Indian	Pakistani	Bangladeshi	Other Asian	Caribbean	Black African	Chinese	Other	Not reported	Total
**Duration of HRT use, median (IQR) days**											
**Any HRT**	957 (338-2207)	713 (267-1918)	715 (267-1830)	780 (308-1828)	744 (256-1853)	946 (310-2154)	725 (252-1758)	652 (244-1618)	748 (294-1816)	921 (333-2120)	939 (335-2183)
Any oestrogen	657 (257-1834)	562 (227-1583)	539 (195-1526)	544 (155-1387)	633 (198-1668)	816 (235-2054)	574 (193-1460)	465 (182-1304)	578 (232-1515)	642 (253-1735)	652 (255-1815)
Oral oestrogen	1312 (417-2778)	1063 (345-2282)	948 (362-2130)	852 (227-1738)	1238 (499-2378)	1032 (309-2387)	745 (246-1910)	712 (160-2324)	1075 (328-2295)	1269 (463-2676)	1294 (413-2743)
Transdermal oestrogen	471 (200-1197)	447 (194-1178)	407 (154-945)	407 (135-1088)	442 (160-1211)	603 (184-1590)	470 (172-1214)	434 (174-1019)	462 (204-1134)	483 (205-1204)	471 (199-1197)
Conjugated oestrogen	1173 (350-2701)	1405 (528-2633)	868 (337-1984)	912 (266-2372)	1252 (358-2365)	926 (298-1840)	1040 (333-2000)	529 (130-1375)	1126 (314-2282)	1274 (416-2707)	1170 (350-2673)
Topical vaginal oestrogen	543 (213-957)		-						-	706 (314-952)	526 (189-945)
Implant oestrogen	903 (356-1506)									836 (344-1328)	928 (356-1545)
Any oestrogen-progestogen	793 (273-1905)	611 (216-1652)	637 (232-1702)	752 (306-1758)	633 (215-1660)	687 (244-1731)	674 (238-1661)	612 (231-1532)	655 (245-1606)	812 (282-1937)	782 (270-1890)
Oral oestrogen-progestogen	995 (337-2081)	897 (297-1955)	866 (272-1854)	830 (333-1915)	798 (269-1815)	906 (314-1896)	784 (266-1732)	864 (276-1835)	848 (287-1802)	984 (330-2104)	985 (335-2067)
Transdermal oestrogen-progestogen	331 (132-751)	293 (125-690)	323 (119-688)	452 (160-985)	329 (135-722)	366 (155-975)	338 (154-820)	361 (114-738)	352 (139-768)	368 (149-826)	333 (133-756)

Any progestogen	1112 (281-2809)	754 (203-2050)	796 (204-2110)	627 (151-1840)	810 (193-2069)	721 (181-1981)	780 (167-1954)	741 (194-2060)	799 (230-2146)	1118 (292-2618)	1079 (275-2711)
Oral progestogen	364 (161-759)	402 (149-1004)	421 (130-1299)	373 (110-1173)	354 (138-878)	314 (115-812)	265 (92-663)	328 (123-629)	348 (138-759)	395 (170-800)	365 (160-770)
Vaginal progestogen	182 (50-700)	166 (51-732)	216 (45-886)	156 (62-428)	149 (39-692)	140 (57-517)	123 (47-656)	83 (33-246)	162 (49-692)	220 (58-940)	177 (50-701)
IUD progestogen											
Tibolone	362 (106-1344)	344 (155-1021)	275 (120-665)	268 (177-561)	283 (85-793)	236 (60-894)	127 (57-334)	797 (94-971)	241 (85-1147)	426 (98-1181)	351 (104-1315)

Supplementary Table 8 – Univariable and multivariable logistic regression for two or more prescriptions of any type of HRT. Multivariable odds ratios are adjusted for age, ethnic group, quintile of Townsend deprivation and region of England

	Unadjusted OR (95% CI)	p	Multivariable OR (95% CI)	p
Age at cohort entry (years)	1.03 (1.03, 1.03)	<0.0001	1.02 (1.02, 1.02)	<0.0001
**Ethnicity**				
White	1.00	<0.0001	1.00	<0.0001
Indian	0.33 (0.32, 0.34)		0.39 (0.37, 0.40)	
Pakistani	0.24 (0.23, 0.26)		0.30 (0.28, 0.31)	
Bangladeshi	0.21 (0.20, 0.23)		0.34 (0.32, 0.37)	
Other Asian	0.19 (0.19, 0.20)		0.25 (0.23, 0.26)	
Caribbean	0.33 (0.32, 0.35)		0.48 (0.46, 0.50)	
Black African	0.14 (0.13, 0.14)		0.20 (0.19, 0.21)	
Chinese	0.15 (0.14, 0.16)		0.18 (0.17, 0.19)	
Other	0.35 (0.34, 0.36)		0.46 (0.45, 0.47)	
Not reported	0.49 (0.48, 0.50)		0.50 (0.49, 0.51)	
**Quintile of Townsend deprivation**				
1 – most affluent	1.00	<0.0001	1.00	<0.0001
2	0.87 (0.86, 0.88)		0.92 (0.91, 0.93)	
3	0.69 (0.68, 0.69)		0.80 (0.79, 0.80)	
4	0.53 (0.52, 0.54)		0.68 (0.67, 0.69)	
5 – most deprived	0.37 (0.37, 0.38)		0.59 (0.58, 0.59)	
Not reported				
**Region**		<0.0001		<0.0001
East Midlands	2.02 (1.96, 2.07)		1.26 (1.22, 1.29)	

East of England	2.04 (2.00, 2.07)		1.27 (1.25, 1.30)	
London (Ref)	1.00		1.00	
North East	1.93 (1.88, 1.98)		1.25 (1.22, 1.28)	
North West	2.13 (2.10, 2.15)		1.36 (1.35, 1.38)	
South Central	2.08 (2.05, 2.11)		1.34 (1.32, 1.35)	
South East	2.09 (2.06, 2.11)		1.31 (1.30, 1.33)	
South West	2.32 (2.29, 2.36)		1.41 (1.39, 1.44)	
West Midlands	1.85 (1.82, 1.88)		1.24 (1.22, 1.26)	
Yorkshire & Humber	2.13 (2.08, 2.18)		1.33 (1.30, 1.37)	

Supplementary Table 9. Sensitivity analysis. Univariable and multivariable Cox regression for: **a.** one or more or **b.** three or more prescriptions of any type of HRT. Hazard ratios are adjusted for ethnic group, quintile of Townsend deprivation and region of England

	**a. One or more prescriptions**				**b. Three or more prescriptions**			
	**Unadjusted**		**Multivariable**		**Unadjusted**		**Multivariable**	
	**HR (95% CI)**	**p**	**HR (95% CI)**	**p**	**HR (95% CI)**	**p**	**HR (95% CI)**	**p**
**Ethnicity**								
White	1.00	<0.0001	1.00	<0.0001	1.00	<0.0001	1.00	<0.0001
Indian	0.85 (0.85, 0.86)		0.88 (0.87, 0.88)		0.88 (0.87, 0.88)		0.90 (0.89, 0.90)	
Pakistani	0.84 (0.83, 0.85)		0.86 (0.85, 0.87)		0.86 (0.85, 0.87)		0.88 (0.87, 0.89)	
Bangladeshi	0.83 (0.82, 0.84)		0.89 (0.87, 0.90)		0.86 (0.84, 0.87)		0.91 (0.89, 0.92)	
Other Asian	0.82 (0.81, 0.83)		0.85 (0.84, 0.86)		0.85 (0.85, 0.86)		0.88 (0.87, 0.89)	
Caribbean	0.85 (0.84, 0.86)		0.91 (0.90, 0.92)		0.88 (0.87, 0.89)		0.92 (0.91, 0.93)	
Black African	0.81 (0.80, 0.81)		0.86 (0.85, 0.86)		0.84 (0.84, 0.85)		0.89 (0.88, 0.89)	

Chinese	0.80 (0.79, 0.82)		0.83 (0.82, 0.84)		0.85 (0.83, 0.86)		0.86 (0.85, 0.88)	
Other	0.85 (0.85, 0.86)		0.89 (0.88, 0.90)		0.88 (0.88, 0.89)		0.91 (0.91, 0.92)	
Missing	0.88 (0.88, 0.89)		0.88 (0.88, 0.89)		0.91 (0.90, 0.91)		0.91 (0.91, 0.92)	
**Quintile of Townsend deprivation**								
1 – most affluent	1.00	<0.0001	1.00	<0.0001	1.00	<0.0001	1.00	<0.0001
2	0.97 (0.97, 0.98)		0.98 (0.98, 0.99)		0.98 (0.97, 0.98)		0.98 (0.98, 0.99)	
3	0.93 (0.93, 0.93)		0.95 (0.95, 0.96)		0.94 (0.94, 0.94)		0.96 (0.95, 0.96)	
4	0.89 (0.89, 0.90)		0.93 (0.93, 0.94)		0.91 (0.91, 0.91)		0.94 (0.94, 0.94)	
5 – most deprived	0.85 (0.85, 0.86)		0.92 (0.91, 0.92)		0.88 (0.87, 0.88)		0.93 (0.92, 0.93)	
**Region**		<0.0001		<0.0001		<0.0001		<0.0001
East Midlands	1.12 (1.10, 1.13)		1.04 (1.02, 1.05)		1.10 (1.08, 1.11)		1.03 (1.02, 1.04)	
East of England	1.12 (1.11, 1.13)		1.04 (1.03, 1.05)		1.09 (1.09, 1.10)		1.03 (1.02, 1.04)	
London (Ref)	1.00		1.00		1.00		1.00	
North East	1.12 (1.11, 1.13)		1.04 (1.03, 1.05)		1.08 (1.07, 1.09)		1.02 (1.01, 1.03)	
North West	1.14 (1.13, 1.14)		1.06 (1.05, 1.06)		1.10 (1.10, 1.11)		1.04 (1.04, 1.05)	
South Central	1.13 (1.12, 1.13)		1.05 (1.04, 1.06)		1.10 (1.09, 1.10)		1.04 (1.03, 1.04)	
South East	1.13 (1.12, 1.13)		1.05 (1.04, 1.05)		1.10 (1.09, 1.10)		1.04 (1.03, 1.04)	
South West	1.15 (1.15, 1.16)		1.07 (1.06, 1.07)		1.12 (1.11, 1.13)		1.05 (1.04, 1.06)	
West Midlands	1.10 (1.10, 1.11)		1.04 (1.03, 1.04)		1.08 (1.07, 1.08)		1.02 (1.02, 1.03)	
Yorkshire & Humber	1.13 (1.12, 1.15)		1.05 (1.04, 1.06)		1.10 (1.09, 1.11)		1.04 (1.03, 1.05)	

Supplementary table 10 - Multivariable Cox regression for two or more prescriptions of any type of HRT after 1st January 2016. Hazard ratios are adjusted for ethnic group, quintile of Townsend deprivation and region of England

	**Multivariable HR (95% CI)**	**p**
**Ethnicity**		
White	1.00	<0.0001
Indian	0.94 (0.93, 0.94)	
Pakistani	0.93 (0.92, 0.94)	
Bangladeshi	0.94 (0.92, 0.95)	
Other Asian	0.92 (0.91, 0.93)	
Caribbean	0.95 (0.94, 0.96)	
Black African	0.93 (0.92, 0.94)	
Chinese	0.91 (0.90, 0.93)	
Other	0.95 (0.94, 0.96)	
Not reported	0.94 (0.93, 0.95)	
**Quintile of Townsend deprivation**		
1 – most affluent	1.00	<0.0001
2	0.99 (0.98, 0.99)	
3	0.97 (0.96, 0.97)	
4	0.95 (0.95, 0.95)	
5 – most deprived	0.94 (0.93, 0.94)	
**Region**		
East Midlands	1.00 (0.99, 1.01)	0.848

East of England	1.01 (1.00, 1.02)	0.064
London (Ref)	1.00	
North East	0.98 (0.98, 0.99)	0.003
North West	1.01 (1.00, 1.01)	0.0002
South Central	1.01 (1.01, 1.02)	<0.0001
South East	1.02 (1.02, 1.03)	<0.0001
South West	1.03 (1.02, 1.03)	<0.0001
West Midlands	1.00 (1.00, 1.01)	0.254
Yorkshire & Humber	1.01 (1.00, 1.02)	0.232

Supplementary table 11. Cox regression for two or more prescriptions of any HRT including interaction term between ethnicity and quintile of deprivation

Interaction	Variable	OR (95% CI)	p
**Interactions between ethnicity and SES**			
White	1 – most affluent	1.00	.
White	2	1.00	.
White	3	1.00	.
White	4	1.00	.
White	5 – most deprived	1.00	.
Indian	1 – most affluent	0.88 (0.87, 0.90)	.
Indian	2	0.88 (0.86, 0.89)	<0.0001
Indian	3	0.88 (0.87, 0.90)	
Indian	4	0.90 (0.88, 0.92)	
Indian	5 – most deprived	0.91 (0.89, 0.94)	

Pakistani	1 – most affluent	0.88 (0.87, 0.90)	<0.0001
Pakistani	2	0.88 (0.86, 0.89)	
Pakistani	3	0.88 (0.87, 0.90)	
Pakistani	4	0.90 (0.88, 0.92)	
Pakistani	5 – most deprived	0.91 (0.89, 0.94)	
Bangladeshi	1 – most affluent	0.86 (0.79, 0.94)	<0.0001
Bangladeshi	2	0.86 (0.81, 0.91)	
Bangladeshi	3	0.87 (0.83, 0.91)	
Bangladeshi	4	0.89 (0.86, 0.92)	
Bangladeshi	5 – most deprived	0.93 (0.91, 0.95)	
Other Asian	1 – most affluent	0.85 (0.82, 0.87)	<0.0001
Other Asian	2	0.85 (0.83, 0.87)	
Other Asian	3	0.86 (0.84, 0.88)	
Other Asian	4	0.88 (0.86, 0.90)	
Other Asian	5 – most deprived	0.91 (0.88, 0.93)	
Caribbean	1 – most affluent	0.89 (0.85, 0.94)	<0.0001
Caribbean	2	0.90 (0.87, 0.94)	
Caribbean	3	0.90 (0.88, 0.93)	
Caribbean	4	0.92 (0.90, 0.94)	
Caribbean	5 – most deprived	0.94 (0.92, 0.96)	
Black African	1 – most affluent	0.83 (0.80, 0.86)	<0.0001
Black African	2	0.84 (0.81, 0.86)	
Black African	3	0.85 (0.84, 0.87)	

Black African	4	0.88 (0.86, 0.89)	
Black African	5 – most deprived	0.90 (0.89, 0.91)	
Chinese	1 – most affluent	0.82 (0.80, 0.85)	<0.0001
Chinese	2	0.83 (0.80, 0.86)	
Chinese	3	0.85 (0.82, 0.88)	
Chinese	4	0.87 (0.84, 0.90)	
Chinese	5 – most deprived	0.89 (0.87, 0.92)	
Other	1 – most affluent	0.90 (0.88, 0.92)	<0.0001
Other	2	0.89 (0.87, 0.91)	
Other	3	0.89 (0.88, 0.91)	
Other	4	0.91 (0.89, 0.92)	
Other	5 – most deprived	0.93 (0.92, 0.94)	
Not reported	1 – most affluent	0.90 (0.89, 0.91)	<0.0001
Not reported	2	0.90 (0.89, 0.91)	
Not reported	3	0.90 (0.89, 0.91)	
Not reported	4	0.91 (0.89, 0.92)	
Not reported	5 – most deprived	0.93 (0.91, 0.94)	

Supplementary table 12 - Multinomial logistic regression on prescriptions of oral or transdermal oestrogen preparations, 0, 1 or 2+ prescriptions. a. Oral oestrogen and b. transdermal oestrogen

		a. Oral oestrogen		b. Transdermal oestrogen	
Numbers of prescriptions	**Variable**	**RRR (95% CI)**	**p**	**RRR (95% CI)**	**p**
**Ethnicity**					
1	White	1.00 (1.00, 1.00)	.	1.00 (1.00, 1.00)	.
1	Indian	0.61 (0.54, 0.69)	<0.0001	0.57 (0.53, 0.61)	<0.0001
1	Pakistani	0.50 (0.42, 0.59)	<0.0001	0.40 (0.36, 0.45)	<0.0001
1	Bangladeshi	0.44 (0.33, 0.59)	<0.0001	0.38 (0.31, 0.46)	<0.0001
1	Other Asian	0.37 (0.31, 0.45)	<0.0001	0.34 (0.30, 0.38)	<0.0001
1	Caribbean	0.86 (0.74, 0.99)	0.041	0.64 (0.57, 0.70)	<0.0001
1	Black African	0.42 (0.36, 0.48)	<0.0001	0.31 (0.28, 0.34)	<0.0001
1	Chinese	0.20 (0.14, 0.29)	<0.0001	0.21 (0.17, 0.26)	<0.0001
1	Other	0.49 (0.44, 0.55)	<0.0001	0.56 (0.53, 0.60)	<0.0001
1	Not reported	0.51 (0.46, 0.55)	<0.0001	0.47 (0.44, 0.49)	<0.0001
**Townsend Deprivation**					
1	1 – most affluent	1.00	.	1.00 (1.00, 1.00)	.
1	2	1.08 (1.04, 1.13)	0.0003	0.92 (0.90, 0.94)	<0.0001
1	3	1.04 (0.99, 1.09)	0.1244	0.81 (0.78, 0.83)	<0.0001
1	4	0.98 (0.93, 1.04)	0.5742	0.68 (0.66, 0.70)	<0.0001
1	5 – most deprived	0.87 (0.81, 0.93)	<0.0001	0.57 (0.54, 0.59)	<0.0001
**Region**					
1	East Midlands	1.66 (1.47, 1.87)	<0.0001	0.89 (0.82, 0.96)	0.005
1	East of England	1.48 (1.36, 1.63)	<0.0001	1.21 (1.15, 1.27)	<0.0001
1	London	1.00	.	1.00 (1.00, 1.00)	.
1	North East	1.59 (1.42, 1.78)	<0.0001	0.92 (0.86, 0.99)	<0.0001
1	North West	1.71 (1.61, 1.81)	<0.0001	1.09 (1.06, 1.13)	<0.0001
1	South Central	1.76 (1.66, 1.88)	<0.0001	1.22 (1.17, 1.27)	<0.0001

1	South East	1.55 (1.46, 1.65)	<0.0001	1.31 (1.26, 1.36)	<0.0001
1	South West	1.71 (1.60, 1.84)	<0.0001	1.28 (1.23, 1.34)	<0.0001
1	West Midlands	1.74 (1.63, 1.86)	<0.0001	1.12 (1.08, 1.17)	<0.0001
1	Yorkshire & Humber	1.52 (1.37, 1.70)	<0.0001	1.07 (1.00, 1.15)	0.040
**Ethnicity**					
2	White	1.00 (1.00, 1.00)	.	1.00 (1.00, 1.00)	.
2	Indian	0.34 (0.32, 0.37)	<0.0001	0.40 (0.39, 0.42)	<0.0001
2	Pakistani	0.25 (0.22, 0.28)	<0.0001	0.26 (0.24, 0.28)	<0.0001
2	Bangladeshi	0.26 (0.21, 0.32)	<0.0001	0.21 (0.19, 0.25)	<0.0001
2	Other Asian	0.23 (0.20, 0.26)	<0.0001	0.22 (0.21, 0.24)	<0.0001
2	Caribbean	0.62 (0.56, 0.68)	<0.0001	0.55 (0.52, 0.58)	<0.0001
2	Black African	0.17 (0.15, 0.20)	<0.0001	0.18 (0.17, 0.19)	<0.0001
2	Chinese	0.15 (0.12, 0.19)	<0.0001	0.17 (0.15, 0.19)	<0.0001
2	Other	0.36 (0.34, 0.39)	<0.0001	0.48 (0.47, 0.50)	<0.0001
2	Not recorded	0.44 (0.42, 0.46)	<0.0001	0.46 (0.45, 0.48)	<0.0001
**Townsend Deprivation**					
2	1 – most affluent	1.02 (0.99, 1.04)	.	1.00 (1.00, 1.00)	.
2	2	1.04 (1.01, 1.06)	0.161	0.88 (0.87, 0.89)	<0.0001
2	3	0.99 (0.96, 1.02)	0.004	0.71 (0.70, 0.72)	<0.0001
2	4	0.88 (0.85, 0.91)	0.443	0.56 (0.55, 0.57)	<0.0001
2	5 – most deprived	1.02 (0.99, 1.04)	<0.0001	0.44 (0.43, 0.46)	<0.0001
**Region**					
2	East Midlands	2.32 (2.18, 2.47)	<0.0001	0.98 (0.94, 1.02)	0.336
2	East of England	1.91 (1.82, 2.01)	<0.0001	1.17 (1.14, 1.21)	<0.0001
2	London	1.00 (1.00, 1.00)	.	1.00 (1.00, 1.00)	.
2	North East	2.36 (2.23, 2.50)	0.0002	0.87 (0.84, 0.91)	0.0002
2	North West	2.32 (2.25, 2.40)	<0.0001	1.11 (1.09, 1.13)	<0.0001

2	South Central	2.28 (2.20, 2.36)	<0.0001	1.33 (1.31, 1.36)	<0.0001
2	South East	1.99 (1.92, 2.07)	<0.0001	1.44 (1.41, 1.47)	<0.0001
2	South West	2.20 (2.12, 2.29)	<0.0001	1.39 (1.36, 1.42)	<0.0001
2	West Midlands	2.33 (2.25, 2.42)	<0.0001	1.18 (1.15, 1.20)	<0.0001
2	Yorkshire & Humber	2.12 (2.01, 2.24)	<0.0001	1.09 (1.05, 1.13)	<0.0001

Supplementary table 13 – Logistic regression comparing two or more prescriptions of (a) transdermal oestrogen to oral oestrogen and (b) transdermal oestrogen/progestogen to oral oestrogen/progestogen in the full dataset and for prescriptions from 2016 onwards.

	a Oestrogen				b Oestrogen/Progestogen combined			
	Transdermal-vs Oral				Transdermal-vs Oral			
	All data		Prescriptions from 2016		All data		Prescriptions from 2016	
**Variable**	**OR (95% CI)**	**p**	**OR (95% CI)**	**p**	**OR (95% CI)**	**p**	**OR (95% CI)**	**p**
**Ethnicity**								
White	1.00	.			1.00	.		
Indian	1.25 (0.97, 1.62)	0.090	1.25 (0.76, 2.06)	0.369	1.36 (1.16, 1.60)	0.0002	1.41 (1.13, 1.75)	0.002
Pakistani	1.17 (0.74, 1.85)	0.497	1.27 (0.58, 2.81)	0.552	1.26 (0.99, 1.60)	0.066	1.00 (0.73, 1.38)	0.989
Bangladeshi	1.10 (0.45, 2.64)	0.840	1.96 (0.38, 10.14)	0.424	1.22 (0.85, 1.76)	0.287	1.35 (0.82, 2.24)	0.241
Other Asian	1.18 (0.73, 1.90)	0.503	1.91 (0.79, 4.59)	0.149	1.18 (0.91, 1.54)	0.218	1.10 (0.77, 1.57)	0.588
Caribbean	1.14 (0.85, 1.54)	0.381	0.68 (0.37, 1.26)	0.225	1.28 (1.05, 1.58)	0.017	1.04 (0.78, 1.39)	0.791
Black African	1.42 (0.94, 2.15)	0.093	1.63 (0.76, 3.48)	0.211	1.54 (1.22, 1.93)	0.0002	1.33 (0.97, 1.82)	0.073
Chinese	1.97 (0.78, 4.94)	0.150	1.00 (1.00, 1.00)	.	1.50 (0.95, 2.36)	0.080	0.95 (0.48, 1.88)	0.876
Other	1.34 (1.08, 1.66)	0.007	1.58 (1.11, 2.26)	0.011	1.24 (1.09, 1.40)	0.0007	1.24 (1.05, 1.46)	0.020
Not reported	1.06 (0.90, 1.24)	0.510	1.16 (0.85, 1.59)	0.338	1.07 (0.97, 1.18)	0.182	1.12 (0.98, 1.28)	0.090
**Deprivation quintile**								
1 – most affluent	1.00				1.00			
2	0.97 (0.90, 1.03)	0.312	0.92 (0.81, 1.04)	0.16	0.98 (0.93, 1.03)	0.391	0.97 (0.91, 1.03)	0.276
3	0.87 (0.81, 0.94)	0.0005	0.94 (0.81, 1.08)	0.360	0.92 (0.88, 0.97)	0.004	0.93 (0.86, 0.99)	0.033
4	0.83 (0.76, 0.91)	<0.0001	0.92 (0.78, 1.08)	0.299	0.89 (0.84, 0.95)	0.0004	0.91 (0.84, 0.99)	0.028
5 – most deprived	0.77 (0.68, 0.87)	<0.0001	0.94 (0.75, 1.18)	0.596	0.89 (0.82, 0.96)	0.002	0.89 (0.80, 0.98)	0.022
**Region**								
East Midlands	0.93 (0.76, 1.14)	0.4932	1.05 (0.71, 1.54)	0.817	0.95 (0.83, 1.08)	0.402	0.97 (0.82, 1.15)	0.721

East of England	0.78 (0.67, 0.91)	0.002	0.85 (0.64, 1.13)	0.256	0.81 (0.74, 0.89)	<0.0001	0.82 (0.72, 0.94)	0.004
London	1.00	.			1.00	.		
North East	0.65 (0.53, 0.78)	<0.0001		0.080	0.82 (0.73, 0.93)	0.003	0.92 (0.77, 1.09)	0.330
North West	0.85 (0.77, 0.95)	0.002	0.71 (0.48, 1.04)	0.478	0.81 (0.76, 0.86)	<0.0001	0.80 (0.74, 0.87)	0
South Central	0.86 (0.77, 0.96)	0.008	0.94 (0.78, 1.12)	0.532	0.86 (0.80, 0.92)	<0.0001	0.86 (0.78, 0.94)	0.002
South East	0.96 (0.86, 1.07)	0.491	1.06 (0.88, 1.29)	0.174	0.93 (0.87, 1.00)	0.056	0.90 (0.82, 0.98)	0.023
South West	0.87 (0.78, 0.98)	0.026	1.15 (0.94, 1.39)	0.470	0.89 (0.83, 0.96)	0.004	0.86 (0.78, 0.95)	0.003
West Midlands	0.84 (0.75, 0.94)	0.003	1.08 (0.88, 1.32)	0.352	0.85 (0.79, 0.92)	<0.0001	0.86 (0.77, 0.95)	0.002
Yorkshire & Humber	0.84 (0.70, 1.00)	0.049	0.90 (0.73, 1.12)	0.835	0.80 (0.71, 0.90)	0.0001	0.82 (0.71, 0.96)	0.012

Supplementary table 14 – Cox regression comparing two or more prescriptions of HRT after adjustment for smoking status, BMI and cardiovascular comorbidities at baseline

	Any HRT	
	**Multivariable HR (95% CI)**	**p**
**Ethnicity**		
White	1.00	<0.0001
Indian	0.86 (0.85, 0.87)	
Pakistani	0.86 (0.85, 0.87)	
Bangladeshi	0.87 (0.85, 0.88)	
Other Asian	0.84 (0.83, 0.85)	
Caribbean	0.89 (0.88, 0.90)	
Black African	0.85 (0.84, 0.86)	
Chinese	0.83 (0.81, 0.85)	
Other	0.90 (0.89, 0.90)	
Not reported	0.92 (0.91, 0.92)	
**Quintile of Townsend deprivation**		
1 – most affluent	1.00	<0.0001
2	0.98 (0.98, 0.99)	
3	0.96 (0.96, 0.97)	
4	0.94 (0.94, 0.95)	
5 – most deprived	0.92 (0.92, 0.93)	

NIHR | National Institute for Health and Care Research

PROTOCOL

TITLE

Quantifying disparities in HRT prescribing in the UK. An analysis of routinely-collected healthcare data

Chief Investigator

Jennifer Hirst, University of Oxford

Co-Chief Investigator: Julia Hippisley-Cox, University of Oxford

Co-Investigators:

Sarah Hillman, University of Warwick

Sharon Dixon, PPI lead, University of Oxford

Carol Coupland, University of Oxford

PPI co-applicant: Lynn Tatnell

Funder: National Institute for Health and Care Research (NIHR) under its Research for Patient Benefit (RfPB) Programme (Grant Reference Number NIHR204901).

Abstract

Research question
Are there ethnic and social disparities in HRT uptake in the UK?

Background
Menopause symptoms can be severe in some women and substantially impact on quality of life. Hormone replacement therapy (HRT) is effective in easing these symptoms and offers additional benefits in slowing progress of osteoporosis. Some types of HRT increase the risks of breast cancer or thrombosis and some women with problematic menopausal symptoms may be choosing not taking HRT despite having a low personal risk. We also know that women from GP practices in low-income areas are least likely to be taking HRT, but we do not know at an individual level which women are taking HRT or whether these are women with the most problematic symptoms.

This research will identify factors associated with severe menopause symptoms and describe characteristics of women who are taking different types of HRT and those who are not. This information will be used to inform future work to develop a risk calculator to show a woman's individual risks or benefits from taking HRT.

Aims
To describe HRT uptake and identify factors associated with HRT use in the UK with a specific focus on ethnicity, comorbidity and deprivation.

Objectives

a. To determine the overall uptake of HRT in women of menopausal age and describe uptake of different types of HRT, duration of use and demographics of women taking HRT and those who are not over a 10-year period.
b. To identify factors associated with HRT uptake

Methods
Systematic review and meta-analysis will be used to summarise factors that are known to be associated with HRT use and severe symptoms of menopause from existing literature.

QResearch, a database of anonymised UK primary care records we will be used to describe data on HRT use over the past 10 year. The cohort will include all women aged 45-60 years.

We will report how many women are currently taking different HRT formulations and describe uptake by women's demographics including geographic region, ethnicity and socioeconomic status. We will use logistic regression to identify factors associated with HRT uptake to establish which women are most and least likely to be HRT users.
We will describe women who have consulted to discuss menopause symptoms. We will use logistic regression to identify factors associated with this.

Timelines: This is part of a wider 15-month project

Anticipated impact and dissemination
This information will provide the first UK data on women are taking HRT, identify a cohort who are not taking HRT and estimate a potential unmet need. It will help to inform future research, for example, targeting a population for qualitative work and those who may benefit most from more information.

We will work with patient representatives from diverse backgrounds to make sure our work is relevant and that we share the results with the right stakeholders. We will present results at conferences and publish findings in academic journals and share our findings through menopause support groups, charities, women's groups and through social media networks.

Quantifying disparities in HRT prescribing in the UK. An analysis of routinely-collected healthcare data.

Research plan and protocol

Background and Rationale

Symptoms of the menopause usually last several years and can affect sleep and quality of life if problematic or severe.(2) Hormone replacement therapy (HRT) is known to be effective at treating problematic symptoms of menopause.(3) However, over the years, there have been a number of studies that have reported adverse health outcomes from HRT use, particularly breast cancer,(4) which has resulted in reductions in prescribing.(5) The UK's National Institute for Health and Care Excellence (NICE) recommends an individualised approach to treatment(6) using tables to help explain risks.(7)

Recent reports have quantified the association between HRT and adverse health outcomes, including thromboembolism(8, 9) and breast cancer,(10, 11) particularly for those with BRCA gene mutations.(10) Women who go through menopause at a younger age are at higher risk of cardiovascular events,(12) and these risks remain similar irrespective of whether or not the woman received HRT. Menopause also has an adverse effect on bone health and increases the risk of fracture,(13) which is significantly reduced with HRT treatment.(4) Furthermore, there is now a greater understanding about how the risk of breast cancer varies with different HRT formulations, duration of use and personal characteristics.(11, 14, 15) It is therefore important that decisions about HRT use are considered in the context of severity of symptoms. For example, because there is an increased risk of breast cancer with some types of HRT, women with a family history of breast cancer will want to weigh up the risks and benefits of HRT treatment and may consider using a formulation containing a hormone which is associated with a lower risk of breast cancer, such as estradiol or dydrogesterone, if appropriate for them.(11) Women with a family history of osteoporosis, on the other hand, may decide that there is an overall benefit to starting HRT. Clear communication about the risks and benefits of HRT at an individual level is important to help women understand how HRT may impact on them and their future health.

There are tools used by clinicians in the UK to help discuss HRT risks, but these are tables showing overall numbers,(6, 7) which do not facilitate discussion of individual risk in terms of personal characteristics or risks due to family or personal history. Furthermore, there is less information about different HRT combinations and newer types of HRT. There are known inequalities in HRT prescribing in the UK, with areas of highest deprivation having the lowest HRT prescribing rates.(16) The reasons for this are not understood, but it may be because communication about menopause and the risks and benefits of available treatments is not taking place in clinical consultations.

It is known that some lifestyle factors and health conditions which are most prevalent in areas of high social deprivation including diabetes,(17) obesity(18) and smoking(19, 20) are associated with severe menopause symptoms. A systematic review and meta-analysis reported factors associated with age at menopause(21) and found that higher educational attainment, higher occupational level, higher levels of physical activity and being in Europe, Australia and US are associated with older age at menopause. Smoking was strongly associated with younger age at menopause and this was most noticeable in economically deprived regions.(21) Additionally, the Study of Women's Health Across the Nation (SWAN) in the United States, which followed up over 3000 women since 1995, found that Black

women experienced menopause 8 months earlier than White women.(22) Younger age at menopause is associated with adverse health outcomes in post-menopausal women.(23)

Although average duration of menopausal symptoms is seven years,(2) this varies by population characteristics. Those with lower educational attainment having a longer duration of symptoms compared with those with higher educational attainment. Ethnicity is also associated with duration of symptoms, with Hispanic and Chinese women having the shortest duration of symptoms with a median duration of <6 years and African American women having the longest duration of symptoms at a median over >10 years.(2) The SWAN study found a higher burden and duration of symptoms, including vasomotor symptoms (hot flashes and night sweats), clinically significant depressive symptoms and lower quality of life amongst Black women compared to White women.(24)

These data suggest that women from ethnic minority backgrounds and more deprived women may experience longer duration of symptoms or more problematic symptoms of menopause, yet UK data from 2018 found that HRT prescribing was lowest in GP practices in areas of highest social deprivation.(16) This suggests there may be women living with problematic symptoms of menopause who are not receiving treatment, but the study did not explore HRT prescribing stratified by individual characteristics such as ethnicity or socioeconomic status, as the data used were at GP-practice level. There has been little research on severity of menopause symptoms and HRT use by sociodemographic characteristics including ethnicity in the UK. However, a qualitative study from 2007 looking at black and minority ethnic women's perceptions of menopause and the use of HRT found that there was inadequate information available to them regarding HRT and most of the information did not take their social and cultural diversities into account.(25) Questionnaire data from 2000 suggest that there is a demand for more information amongst Asian women in the UK.(26) Data from the United States show that HRT use in American women was lowest in those in the lowest income groups, those with the lowest education attainment and non-white populations.(27) Overall, the uptake of HRT by socioeconomic class and ethnicity is not well reported, but evidence suggests there may be inequalities in prescribing, despite many of these women experiencing a high burden of menopausal symptom for longer.

The UK government have recently acknowledged that there are stigmas for many women around discussing menopause, that women do not always feel listened to by healthcare professionals and that there are difficulties in accessing HRT.(28) In particular, women called for more information on the menopause, and on treatment options, especially in cases where HRT is not suitable. Access to care and support during menopause is part of the government's strategy to make women's health more accessible and equitable. Understanding the risks and benefits of HRT will form an important part of decision-making on whether to take HRT. However, it is also crucial to understand which women are having the most difficulty accessing the care they need in order to deliver an equitable service.

Review of existing evidence

Risks of HRT

Many prior studies have reported health risks and benefits of HRT use for management of menopausal symptoms.(29) The NICE guidelines on HRT have summarised these risks and recommend an individualised approach to the management and treatment of menopause.(6) Furthermore, NICE have recently announced that they will be updating this guidance which will include data on the effect of HRT on health outcomes.(30) This literature update will provide valuable and up-to-date data on risks and benefits of all types of HRT. The research team at University of Oxford will be able to draw on this as a resource in future work to develop a tool to calculate individual risks and benefits of HRT use and inform a communication tool for use in clinical

consultations with women to discuss menopause and treatment options.

Prevalence of problematic menopause symptoms
A systematic review reported the prevalence of severe menopausal symptoms to be 63% in European women aged 45 to 60.(31) Some reported risk factors associated with the most severe symptoms of menopause include obesity, being a current smoker,(32) having diabetes,(17) low consumption of dairy products, low socioeconomic class,(33) older age, increasing follicle stimulation hormone (FSH) concentrations, being postmenopausal versus premenopausal, using oral contraceptive pill, being in the middle rather than lower quartiles of testosterone concentrations and having more children.(34) Other symptoms reported in the SWAN study to be associated with having menopause symptoms include decreasing level of educational attainment, being from an ethnic minority group, having a higher BMI, past and current smoking and lower levels of physical activity, but symptoms were not stratified by severity in this report.(35) There is some evidence that the types of bothersome symptoms differ between racial groups,(36) which may not always be considered.

HRT prescribing
A recent analysis of UK primary care HRT prescribing data found that there has been an increase in HRT prescribing of HRT, and that the increase was highest in the 50-59 age group and for transdermal preparations and fixed combinations of oestrogen and progesterone.(37) This analysis explored differences in HRT prescribing by age but did not consider any other individual characteristics. Open Prescribing data show that estradiol prescriptions have also increased substantially in the past few years,(38) but they do not have data on differences in uptake by population characteristics. We know that prescribing of HRT is 29% lower in GP practices in the most deprived communities in the UK compared with more affluent GP practices.(16) This is consistent with reports of factors associated with initiation of HRT from other countries,(39, 40) which have found higher HRT uptake in women with higher education status and amongst those informed about the risks of HRT. Two small studies describing HRT uptake in the United States found racial and socioeconomic disparities,(41, 42) but did not explore reasons for low uptake in some populations.

Additionally, the UK data found that there was a higher proportion of prescriptions of oral HRT and lower proportion of transdermal HRT in GP practices in the most deprived areas compared with GP practices in the most affluent areas.(16) As the prevalence of cardiovascular disease is higher in deprived areas(43) and transdermal preparations are recommended for women with a higher risk of thromboembolism or stroke,(6) it may be expected that there should be a higher proportion of prescriptions for transdermal preparations in the most deprived practices. Furthermore, women already taking medications for other conditions may not want the burden of additional pills.

The evidence therefore suggests that there may be a mismatch between the women experiencing the most severe symptoms and those who end up taking HRT. This may mean there is an unmet need in some communities, specifically amongst women from more disadvantaged areas who may have less access to treatment. Therefore, although we know there are disparities in HRT uptake, we currently have no information on which groups of women are most affected and whether there may be lower uptake in some ethnic groups that in others in the UK.

It is therefore important to describe UK HRT prescribing patterns, quantify any disparities in HRT prescribing and understand the scale of potential unmet need using individual-level HRT prescribing data. A large-scale analysis to describe prescriptions of different HRT regimens and identify patient characteristics associated with HRT uptake in the UK (including socioeconomic status and ethnic group) has not previously been carried out.

The longer-term objective of the research team is to develop a risk stratification and communication

tool to help decision-making in HRT. However, for a risk communication tool to be effective, it is important to have a clear understanding of which women have problematic menopause symptoms, and of those, which groups are most or least likely to be accessing HRT. This information is necessary to ensure that communication aids are designed that will help women who are currently not accessing treatment but are most likely to benefit.

Dr Sarah Hillman, co-applicant in this study, has just acquired funding to carry out a piece of qualitative research to establish how women from underserved communities obtain information and access care and support for the menopause, how they experience menopause care and what is needed by women and healthcare professionals to address inequalities in menopause care in general practice. Her work will tie in with this project and may explain some of the findings from this research.

Potential scale of unmet need in the UK
In 2021, there were around 6.7 million women aged 45-59 in the UK.(44) If only 25% of these women have problematic menopause symptoms,(31) there will be 1.6 million women in the UK living with problematic symptoms. University of Oxford's Open Prescribing suggests that there were 430,000 prescriptions for female sex hormones and their modulators in June 2021.(38) Without knowing the frequency of prescriptions, these approximations suggest that as many as two thirds of women in the UK with problematic menopausal symptoms are not being prescribed HRT, but this is a crude calculation without any details of type or duration of treatment. Therefore, the true scale of unmet need may differ substantially from these approximations. In this research, we will quantify the scale of HRT use and describe the populations receiving HRT and those who are not with a specific focus on ethnicity and social deprivation.

The QResearch database is a REC approved research database. It contains detailed anonymised information on a representative sample of over 35 million patients tracking back over 25 years. The data include demographics (age, sex, ethnicity, deprivation, geographical region), diagnoses, referrals, laboratory investigations, symptoms, consultations and prescriptions. Using QResearch, we will be able to determine who has received HRT prescriptions and stratify by type and dose of HRT and by demographics of the population in the context of known characteristics of women who are most likely to experience problematic menopause symptoms.(34) This study will summarise what is already known about menopausal symptoms and HRT uptake from the academic literature and carry out an analysis of the QResearch dataset.

Aims and objectives

Aims
To describe HRT uptake and identify factors associated with HRT use in the UK with a specific focus on ethnicity, comorbidity and deprivation.

Objectives

a. To determine the overall uptake of HRT in women of menopausal age and describe uptake of different types of HRT, duration of use and demographics of women taking HRT and those who are not over a 10-year period.
b. To identify factors associated with HRT uptake

Inclusion criteria
The full cohort will include all women aged between 40 and 60 registered with a GP practice for at

least 1 year during a 10-year period from 2013 to 2022.

Data source and setting
Our analyses will use primary care records from the QResearch database.

Study design
We will use a cohort design

Methods

a. Descriptive analysis of HRT use in the UK over a 10-year period using QResearch.
A descriptive analysis of GP prescribing records held on the QResearch database (35 million patient records derived from 1500 practices in England) will be undertaken to determine the size and characteristics of the exposed population. Results will be presented in tables and graphs.

Data from medical records over the past 10 years will be used. We will use the latest available data and extend back for 10 years (2013-2022). HRT uptake will be defined as those who have at least two prescriptions of the same type of HRT at any time during the 10 years of follow-up. We will describe numbers and characteristics of women taking different types, doses, routes of administration and duration of HRT. For those exposed, numbers and proportions of women with risk factors such as personal and family history of breast cancer, cardiovascular disease, osteoporosis and comorbidities such as diabetes will be reported. We will also describe people living with conditions which may affect uptake, including sickle cell disease, ADHD and autism. We will describe HRT prescribing stratified by ethnicity, socioeconomic status, geographic area, BMI and numbers and types of comorbidities.

We will generate tables and graphs to report demographic characteristics (age, body mass index category, ethnicity using self-assigned ethnic group, quintile of Townsend deprivation score, geographic region of the UK, sub-divided into 10 geographic regions) using appropriate summary statistics of:

- The full cohort of women
- Women taking any formulation or dose of HRT

We will calculate:

- Uptake of different formulations individually and categorised into different groupings as described by previous researchers to include older and newer formulations:
    - oestogen only, oestogen-progesterone or tibiolone formulations.(11)
    - different types of hormone: conjugated equine oestrogen versus estradiol for oestrogen-only preparations, and medroxyprogesterone, levonorgestrel, norethisterone or dydrogesterone for oestrogen-progestogen preparations(11)
    - women who are taking their oestrogen and progesterone separately (namely oestrogen oral or transdermal and progesterone in the form of a mirena or micronized progesterone).
    - different modes of delivery: oral or transdermal preparations of HRT.
- Mean age (with standard deviation) at first HRT prescription and demographics.
- Median(with interquartile range) number of prescriptions stratified by type of HRT
- Duration of use by type of HRT
- Numbers of prescriptions for each year of follow-up

b. Identifying factors associated with HRT uptake in QResearch
A multilevel logistic regression analysis will be used to determine factors associated with HRT prescribing in the full cohort. GP practice will be included as a random effect in the model to account

for similar prescribing behaviours within individual practices.

Study population
The population will be women aged 40-60 experiencing menopause during the 10-year follow-up.

Exposure: Factors associated with HRT use.

Outcome: Two or more prescriptions of the same type of HRT in women aged 40-60 years during the 10-year follow-up.

The multilevel analysis will include covariates associated with HRT use or severe menopause symptoms reported in the literature,(34) including age, BMI, smoking status, ethnicity, socioeconomic status, contraceptive pill use, numbers of comorbidities, individual comorbidities, family history of comorbidities and geographic region of the UK. This will be carried out for use of any type of HRT and separately by grouping into oestogen only, oestogen-progesterone or tibolone, by individual hormone type and transdermal or oral preparations. We will include any other factors identified from the systematic review and meta-analysis if feasible within the dataset.
Some variables reported to be associated with adverse menopausal symptoms: FSH concentrations, testosterone levels, number of children(34) or levels of physical activity
(45) will not be included in the analysis as reliable data will not be available for the majority of women.

A secondary analysis will be conducted to specifically explore any socioeconomic disparities in uptake by stratifying the analysis by quintile of social deprivation (measured using Townsend deprivation score). Ethnic group, geographic region and other factors found to be significantly associated with HRT uptake in the main analysis will be included as covariates.

Results from unadjusted and adjusted analyses to report odds ratios and 95% confidence intervals will be presented in tables. The unadjusted model will include the exposure variable and no covariates. Adjustments will be made separately for confounding factors associated with the increased risk of each HRT outcome and together in the maximally adjustment model. Statistical significance will be assumed at the 1% level. Our studies will be conducted and reported in line with the RECORD and STROBE guidelines for observational studies using routinely collected health data.

Stata version 17SE will be used for the analyses.

Sample size
In 2020 there were 4.6 million women in the 50-60 age category in the UK.(44) QResearch represents 15% of GP practices, suggesting that we will have a cohort of around 700,000 women meeting our inclusion criteria in 2020. Extending back over 10 years, we would expect an additional 300,000 to be within this age range during the follow-up period, giving a total cohort size of approximately 1 million.

If we assume that 15% of women go onto HRT (the outcome)(46) and 1% of the cohort are exposed to a particular factor of interest, we can estimate an odds ratio of 1.09 or more with 5% significance and 90% power.
If 33% take HRT(47) - the minimum odds ratio is 1.07 for 1% exposed
For 40% on HRT(48) it the minimum odds ratio will also be around 1.07 for 1% exposed. For any higher proportions of women are exposed to an individual factors, the minimum detectable odds ratios will be even smaller. Therefore, with around 1,000,000 women we should be able to detect relatively low odds ratios across a range of exposures.

PPI involvement throughout the study:
We will recruit a panel of 4 participants from diverse backgrounds to join us for a total of four meetings during the study. We will do this by reaching out to women who we met with to inform the study application.

In the first meeting with the panel will be introduce the panel to the study team, discuss the study aims and listen to ideas about how results could be shared to reach the most diverse groups of women. We will ask their advice about how we make sense of patterns in HRT uptake that are observed and how we can communicate these findings. We will seek their views on how to communicate what we find.

Project/Research timetable
15-month project – see Gantt chart

Project management
The researcher and JH will meet on a weekly basis throughout the project.
A project management team consisting of the co-applicants and research team will be set up which will meet on a monthly basis for the duration of the study to make key decisions about the methodological approaches, interpret findings, plan next steps and monitor progress.

Ethics / Regulatory approvals
QResearch has ongoing approval from the East Midlands Multi-Centre Research Ethics Committee (Ref: 18/EM/0400)
The University of Oxford is the sole data controller for the datasets which are linked to QResearch (deaths, cancer and hospital data) and the single point of access to the data.

References

1. Office for National Statistics. Census 2021. Ethnic group by age and sex in England and Wales. 2023 [Available from: https://www.ons.gov.uk/peoplepopulationandcommunity/culturalidentity/ethnicity/datasets/ethnicgroupbyageandsexinenglandandwales.
2. Avis NE, Crawford SL, Greendale G, Bromberger JT, Everson-Rose SA, Gold EB, et al. Duration of menopausal vasomotor symptoms over the menopause transition. JAMA Intern Med. 2015;175(4):531-9.
3. Maclennan AH, Broadbent JL, Lester S, Moore V. Oral oestrogen and combined oestrogen/progestogen therapy versus placebo for hot flushes. Cochrane Database Syst Rev. 2004;2004(4):Cd002978.
4. Rossouw JE, Anderson GL, Prentice RL, LaCroix AZ, Kooperberg C, Stefanick ML, et al. Risks and benefits of estrogen plus progestin in healthy postmenopausal women: principal results From the Women's Health Initiative randomized controlled trial. Jama. 2002;288(3):321-33.
5. Iversen L, Delaney EK, Hannaford PC, Black C. Menopause-related workload in general practice 1996-2005: a retrospective study in the UK. Fam Pract. 2010;27(5):499-506.
6. NICE. Menopause: diagnosis and management, NG23 2015 [Available from: https://www.nice.org.uk/guidance/NG23.

7. MHRA. Table 1: Summary of HRT risks and benefits* during current use and current use plus post-treatment from age of menopause up to age 69 years, per 1000 women with 5 years or 10 years use of HRT 2019 [Available from: chrome-extension://efaidnbmnnnibpcajpcglclefindmkaj/https://assets.publishing.service.gov.uk/media/5d680409e5274a1711fbe65a/Table1.pdf.
8. Vinogradova Y, Coupland C, Hippisley-Cox J. Use of hormone replacement therapy and risk of venous thromboembolism: nested case-control studies using the QResearch and CPRD databases. BMJ (Clinical research ed). 2019;364:k4810.
9. Boardman HM, Hartley L, Eisinga A, Main C, Roqué i Figuls M, Bonfill Cosp X, et al. Hormone therapy for preventing cardiovascular disease in post-menopausal women. Cochrane Database Syst Rev. 2015(3):Cd002229.
10. Michaelson-Cohen R, Gabizon-Peretz S, Armon S, Srebnik-Moshe N, Mor P, Tomer A, et al. Breast cancer risk and hormone replacement therapy among BRCA carriers after risk-reducing salpingo-oophorectomy. Eur J Cancer. 2021;148:95-102.
11. Vinogradova Y, Coupland C, Hippisley-Cox J. Use of hormone replacement therapy and risk of breast cancer: nested case-control studies using the QResearch and CPRD databases. BMJ (Clinical research ed). 2020;371:m3873.
12. Zhu D, Chung HF, Dobson AJ, Pandeya N, Giles GG, Bruinsma F, et al. Age at natural menopause and risk of incident cardiovascular disease: a pooled analysis of individual patient data. Lancet Public Health. 2019;4(11):e553-e64.
13. Greendale GA, Huang M, Cauley JA, Liao D, Harlow S, Finkelstein JS, et al. Trabecular Bone Score Declines During the Menopause Transition: The Study of Women's Health Across the Nation (SWAN). J Clin Endocrinol Metab. 2020;105(4):e1872-82.
14. Chlebowski RT, Anderson GL, Aragaki AK, Manson JE, Stefanick ML, Pan K, et al. Association of Menopausal Hormone Therapy With Breast Cancer Incidence and Mortality During Long-term Follow-up of the Women's Health Initiative Randomized Clinical Trials. Jama. 2020;324(4):369-80.
15. Collaborative Group on Hormonal Factors in Breast Cancer. Type and timing of menopausal hormone therapy and breast cancer risk: individual participant meta-analysis of the worldwide epidemiological evidence. Lancet (London, England). 2019;394(10204):1159-68.
16. Hillman S, Shantikumar S, Ridha A, Todkill D, Dale J. Socioeconomic status and HRT prescribing: a study of practice-level data in England. British Journal of General Practice. 2020;70(700):e772-e7.
17. Herber-Gast GC, Mishra GD. Early severe vasomotor menopausal symptoms are associated with diabetes. Menopause. 2014;21(8):855-60.
18. Koo S, Ahn Y, Lim JY, Cho J, Park HY. Obesity associates with vasomotor symptoms in postmenopause but with physical symptoms in perimenopause: a cross-sectional study. BMC Womens Health. 2017;17(1):126.
19. Ziv-Gal A, Flaws JA. Factors that may influence the experience of hot flushes by healthy middle-aged women. J Womens Health (Larchmt). 2010;19(10):1905-14.
20. Thurston RC, Joffe H. Vasomotor symptoms and menopause: findings from the Study of Women's Health across the Nation. Obstet Gynecol Clin North Am. 2011;38(3):489-501.
21. Schoenaker DA, Jackson CA, Rowlands JV, Mishra GD. Socioeconomic position, lifestyle factors and age at natural menopause: a systematic review and meta-analyses of studies across six continents. Int J Epidemiol. 2014;43(5):1542-62.
22. El Khoudary SR, Greendale G, Crawford SL, Avis NE, Brooks MM, Thurston RC, et al. The menopause transition and women's health at midlife: a progress report

from the Study of Women's Health Across the Nation (SWAN). Menopause. 2019;26(10):1213-27.
23. Muka T, Oliver-Williams C, Kunutsor S, Laven JS, Fauser BC, Chowdhury R, et al. Association of Age at Onset of Menopause and Time Since Onset of Menopause With Cardiovascular Outcomes, Intermediate Vascular Traits, and All-Cause Mortality: A Systematic Review and Meta-analysis. JAMA Cardiol. 2016;1(7):767-76.
24. Harlow SD, Burnett-Bowie SM, Greendale GA, Avis NE, Reeves AN, Richards TR, et al. Disparities in Reproductive Aging and Midlife Health between Black and White women: The Study of Women's Health Across the Nation (SWAN). Womens Midlife Health. 2022;8(1):3.
25. OZUZU-NWAIWU J. Black women's perceptions of menopause and the use of HRT. Nursing times. 2007;103(2):34-5.
26. Sethi K, Pitkin J. British-Asian women's views on and attitudes towards menopause and hormone replacement therapy. Climacteric. 2000;3(4):248-53.
27. Friedman-Koss D, Crespo CJ, Bellantoni MF, Andersen RE. The relationship of race/ethnicity and social class to hormone replacement therapy: results from the Third National Health and Nutrition Examination Survey 1988-1994. Menopause. 2002;9(4):264-72.
28. UK Government. Our Vision for the Women's Health Strategy for England. Policy paper 2021 [Available from: https://www.gov.uk/government/publications/our-vision-for-the-womens-health-strategy-for-england/our-vision-for-the-womens-health-strategy-for-england#womens-voices.
29. Hamoda H, Moger S. Looking at HRT in perspective. BMJ (Clinical research ed). 2022;377:o1425.
30. NICE. NICE sets out further details on menopause guideline update 2022 [Available from: https://www.nice.org.uk/news/article/nice-sets-out-further-details-on-menopause-guideline-update.
31. Palacios S, Henderson VW, Siseles N, Tan D, Villaseca P. Age of menopause and impact of climacteric symptoms by geographical region. Climacteric. 2010;13(5):419-28.
32. Anderson DJ, Chung HF, Seib CA, Dobson AJ, Kuh D, Brunner EJ, et al. Obesity, smoking, and risk of vasomotor menopausal symptoms: a pooled analysis of eight cohort studies. Am J Obstet Gynecol. 2020;222(5):478.e1-.e17.
33. Pérez JA, Garcia FC, Palacios S, Pérez M. Epidemiology of risk factors and symptoms associated with menopause in Spanish women. Maturitas. 2009;62(1):30-6.
34. Ford K, Sowers M, Crutchfield M, Wilson A, Jannausch M. A longitudinal study of the predictors of prevalence and severity of symptoms commonly associated with menopause. Menopause. 2005;12(3):308-17.
35. Gold EB, Sternfeld B, Kelsey JL, Brown C, Mouton C, Reame N, et al. Relation of demographic and lifestyle factors to symptoms in a multi-racial/ethnic population of women 40-55 years of age. Am J Epidemiol. 2000;152(5):463-73.
36. Avis NE, Stellato R, Crawford S, Bromberger J, Ganz P, Cain V, et al. Is there a menopausal syndrome? Menopausal status and symptoms across racial/ethnic groups. Soc Sci Med. 2001;52(3):345-56.
37. Alsugeir D, Wei L, Adesuyan M, Cook S, Panay N, Brauer R. Hormone replacement therapy prescribing in menopausal women in the UK: a descriptive study. BJGP Open. 2022.
38. Open Prescribing. 6.4.1: Female sex hormones and their modulators 2022

[Available from: https://openprescribing.net/bnf/060401/.
39. Çilgin H. Predictors of Initiating Hormone Replacement Therapy in Postmenopausal Women: A Cross-Sectional Study. ScientificWorldJournal. 2019;2019:1814804.
40. Manzoli L, Di Giovanni P, Del Duca L, De Aloysio D, Festi D, Capodicasa S, et al. Use of hormone replacement therapy in Italian women aged 50-70 years. Maturitas. 2004;49(3):241-51.
41. Finley C, Gregg EW, Solomon LJ, Gay E. Disparities in hormone replacement therapy use by socioeconomic status in a primary care population. J Community Health. 2001;26(1):39-50.
42. Pershad A, Morris JM, Pace D, Khanna P. Racial disparities in menopausal hormone therapy acceptance: a pilot study. Menopause. 2022;29(11):1263-8.
43. Stringhini S, Carmeli C, Jokela M, Avendaño M, Muennig P, Guida F, et al. Socioeconomic status and the 25 × 25 risk factors as determinants of premature mortality: a multicohort study and meta-analysis of 1·7 million men and women. Lancet (London, England). 2017;389(10075):1229-37.
44. Office for National Statistics. Principal projection - UK population in age groups 2022 [Available from: https://www.ons.gov.uk/peoplepopulationandcommunity/populationandmigration/populationprojections/datasets/tablea21principalprojectionukpopulationinagegroups.
45. Lucas R, Barros H. Life prevalence and determinants of hormone replacement therapy in women living in Porto, Portugal. Maturitas. 2007;57(3):226-32.
46. Parazzini F. Trends of determinants of hormone therapy use in Italian women attending menopause clinics, 1997-2003. Menopause. 2008;15(1):164-70.
47. Oddens BJ, Boulet MJ. Hormone replacement therapy among Danish women aged 45-65 years: prevalence, determinants, and compliance. Obstet Gynecol. 1997;90(2):269-77.
48. Lucas R, Barros H. Life prevalence of hormone replacement therapy and profile of users have not changed among women with self-reported menopause in the last two decades in Porto, Portugal. Climacteric. 2008;11(1):26-31.
