# Supplementary material for: Inequalities in hormone replacement therapy prescribing in UK primary care: population based cohort study
Source: BMJ Med. 2025 Sep 25;4(1):e001349. doi: 10.1136/bmjmed-2025-001349 (PMC12481343; doi:10.1136/bmjmed-2025-001349)
Supplement: Supplementary file 4 [file bmjmed-2025-001349.reviewer_comments.pdf]

PEER REVIEW HISTORY

BMJ Medicine publishes all reviews undertaken for accepted manuscripts. Reviewers are asked to complete a checklist review form and are provided with free text boxes to elaborate on their assessment. These free text comments are reproduced below.

ARTICLE DETAILS

**TITLE (PROVISIONAL)**	Inequalities in hormone replacement therapy prescribing in UK primary care – An analysis of 2 million women using electronic health records
**AUTHORS**	Hirst, Jennifer; Mtika, Wema; Coupland, Carol; Dixon, Sharon; Hippisley-Cox, Julia; hillman, sarah

VERSION 1 - REVIEW

**REVIEWER NAME**	Hickey, Martha
**REVIEWER AFFILIATION**	University of Melbourne, Obstetrics and Gynaecology
**REVIEWER CONFLICT OF INTEREST**	I have published in 2024 with Sarah Hillman<br>I am discussing a joint grant application with Jenny Hirst and Sarah Hillman
**AI DISCLOSURE**	No
**DATE REVIEW RETURNED**	28-Jan-2025

**GENERAL COMMENTS**	Thanks for asking me to review this interesting manuscript. This is a large, GP records based study (not a cohort study as such) of HRT prescribing in the UK, broken down by age, ethnicity and social deprivation, based on postcode.<br><br>Based on the title and abstract, I was confused about what this study is about. The title talks about inequalities in prescribing of HRT but the abstract talks about inequalities in uptake. These are not the same thing and the authors should clarify the focus of their study.<br><br>Why was the age range 40-60 used? The average age at onset of the menopause transition is 47 years, so prior to this most women will be premenopausal (using STRAW criteria). Age at menopause may be younger in UK minority groups might be younger, but this is not yet known.<br><br>The observation that socially advantaged white women are more likely to take HRT is not new and is consistent with large observational studies such as NHS. However, I think this information is new (but not unexpected) for the UK.<br>The conclusion that HRT prescribing represents inequalities is a bit presumptuous. What they have observed is a difference in reporting rates by economic deprivation and minority status. They don't know the reasons for this. These groups may have different menopausal symptoms and/or cultural or personal reasons why they don't wish to take HRT. Based on published observations from LMIC, this might well be the case. Similarly, the evidence shows that white women are more likely to have vasomotor symptoms (the primary indication for HRT) and to ask for treatment. Language is important here. The authors imply that greater prescribing of HRT to these groups would be 1) wanted and 2) beneficial.

	So, why the findings may suggest "health inequities" there are other interpretations which should be acknowleged<br>As recognised by NICE 2024, there is a substantial gap in understanding about the risks and benefits of HRT in minority women. There is also a gap in understanding about the needs of this group for menopause care. This should be highlighted.<br><br>Specific issues<br>In the introduction the authors quote the 2002 MacLennan Cochrane review to support that "HRT treats symptoms of the menopause". As indicated in the Cochrane review title, HRT is effective for vasomotor symptoms. Suggesting efficacy for the vague "symptoms of the menopause" is potentially misleading, given the multiple "symptoms" this might mean. Please revise.<br>Note that NICE guidelines on menopause are now updated<br>Evidence from SWAN about racial differences in vasomotor symptoms has been extremely valuable. However, it is not appropriate to generalise from these AA women to minority groups in the UK - at least not without evidence<br>Regarding the type of HRT, NICE recommend that transdermal preparations are used in those with risk factors for potential risks of HRT such as VTE and stroke. If these risk factors are more prevalent in disadvantaged groups then use of TD preparations should be promoted. Qualitative studies with prescribers may help to determine why this does not happen.<br>I found the comment about "not wanting additional pills" a bit unconvincing (no ref). Many combined TD products require twice weekly changing which may be more annoying than a daily pill!<br>statements about HRT inequalities should not quote evidence from the all party parliamentary group. This group was funded by a pharmaceutical group who manufacture HRT. Suggest sticking with peer reviewed pubs. Specifically, I was not convinced by the suggestion that UK women with the most severe vasomotor symptoms are not getting access to HRT. This seems speculative.<br><br>I am not qualified to discuss the statistical methods<br><br>Results<br>I was interested to see that the mean age at HRT prescription was 49 years. Since the mean age at menopause in the UK is 51 years, this suggests that some women are starting HRT in the menopause transition. The authors should discuss that the safety and efficacy of HRT in this population is very poorly understood, since almost all clinical trials are restricted to postmenopausal women.<br>The study contains interesting (and novel) information about discontinuation of HRT by ethnicity. They report >2 fold % of minority women who had only one prescription of HRT vs white women. Why might this be? This seems very important for the message. Is the issue that these women want/need HRT and are not offered it, are they offered it then discontinue after one prescription or do they decline it? The important data in this study need this information because they can be used to inform practice.

**REVIEWER NAME**	Martin, Florence
**REVIEWER AFFILIATION**	University of Bristol, MRC Integrative Epidemiology Unit
**REVIEWER CONFLICT OF INTEREST**	I am a consultant at IQVIA.
**AI DISCLOSURE**	No
**DATE REVIEW RETURNED**	24-Feb-2025

GENERAL COMMENTS	Thank you to the authors for this interesting work. It is an important study in an underrepresented field, describing HRT utilisation in England using QResearch primary care data (EMIS). Authors have descriptively explored ethnic and socioeconomic differences, as well as prescribing among different comorbidities and characteristics. I have included some suggestions and questions below. I have numbered the pages as reported on the manuscript, not of the proof in its entirety.<br>Abstract<br>Page 2 line 27: not sure what the breakdown of 8.3% and 5.0% is of those receiving HRT? But then what makes up the other 87%. If proportion of the full cohort, what is the other 5.9% if not oral or transdermal. I comment on this later on, but I think when breaking down the types of HRT, the proportions should be out of those taking HRT (as opposed to the full cohort as the denominator).<br>Page 2 line 44: seems like HRT prescribing is systematically lower than I would have expected, which is an important finding too, alongside the inequalities!<br>Introduction<br>Throughout: be consistent with capitalising 'Black' when referring to ethnicity<br>Are there any other studies that report the prevalence of HRT use in the UK? This might be a nice addition to the introduction (e.g., Burkard et al, 2019?) to situate your subsequent findings of HRT use among 40-60 year olds in QResearch, before exploration of relevant subgroups.<br>Something about the risk landscape (DVT, ischaemic stroke, cancer) would be useful here I think, given that you discuss that inequalities in certain comorbidities might drive differences in HRT prescribing among certain groups.<br>Methods<br>Page 5 line 12: if space, I would benefit from a little bit more information about QResearch (namely, where the data is coming from – EMIS) so readers can place the research within their existing understanding of other primary care data repositories like CPRD, rather than having to follow the link, like I did.<br>Page 5 line 29: is Townsend deprivation score individual-level or practice-level? Either way, it is a relatively blunt tool to capture SEP. Of course it's all we have in English EHRs, but important to comment on as I have raised in the discussion.<br>Page 5 line 38: given that Mirena is a brand name, it might be more appropriate to use the generic "intrauterine progesterone-only device" or words to that effect.<br>How were conflicting ethnicity records handled? Presumably there were people in the cohort who have ethnicity recorded more than and in multiple sources (EMIS and HES). In my experience these don't always agree – was this dealt with at all?<br>As I comment on in the results, I'm unsure of the benefit of having both logistic and Cox, particularly where time is important (those with longer follow-up have more chance to be prescribed HRT multiple times and thus be considered to have experienced the outcome under study).<br>Results<br>Page 6 line 56-57: when you say "…received two or more HRT prescriptions at any time…" that is presumably referring to during follow-up right? From the point at which they became eligible (aged ≥40 at the start of 2013 or from the point they turned 40 after the start of the study)? Might be worth clarifying either here or in the

	methods.<br>Page 6 line 56: very similar to the sentence starting line 58 – consider consolidating?<br>Page 7 line 3: I'm struggling to follow these proportions in terms of what their denominator is. Is the denominator the full cohort or those who received ≥2 HRT prescriptions? My vote is for the latter (discussed below). Also, if the groups aren't mutually exclusive, consider informing the reader of this so it is easy to follow.<br>Page 7 line 11: the latter part of this paragraph feels like a comparison between 2013-2023 and 2016-2023. To further aid readability, it might be useful to mirror these parts of the paragraph so that the numbers are easier to compare.<br>Page 8 line 7: interesting that prevalence of prescribing among Caribbean women is less than half but their duration of prescribing is significantly higher? I think more discussion around why this might be useful as I think this finding is interesting.<br>Page 8 lines 13-: the very low ORs attenuate in the Cox models so are they strongly driven by differential follow-up? Proportional hazards probably violated as the "risk" of starting HRT changes over time (most notably post 2015 guidance), which could do with a note in the limitations (similarly commenting on the lack of consideration of length of follow-up in the logistic regression models). Are there changes in proportions of the exposure groups (ethnicity/deprivation etc.) over time which could be contributing to this? Similarly is length of follow-up stratified by exposure groups reported in the supplement? I might have missed it but if not there, that could be helpful in interpreting this.<br>As I raised in the methods, I'm not sure what the benefit of having both logistic regression and Cox is. I would be inclined to say that Cox is more favourable here because it accounts for time in a way that logistic regression doesn't, and you get the same conclusion so seems redundant to have both. If you do really want logistic regression, I would switch them and have Cox in the main manuscript.<br>Page 8 line 47-: what could be driving differences in formulation across subgroups? Weird that the reasons for having a transdermal patch (comorbidities etc.) are higher in the more deprived group but transdermal patch use is lower… But attenuated when restricting to post-2015 guidance. Why are these data not included?<br>Do you have hypotheses regarding differential prescribing? Prescriber sex, education, engagement with women's health? I think the discussion could benefit from a bit more about this.<br>Page 9 line 23-: could engagement with primary care services driving some of the differing proportions of comorbidities among those prescribed HRT compared with those who were not? Some of the findings are a useful sense check but don't necessarily need flagging in the text (here or in the discussion at length); for example, it's expected that those with breast cancer aren't being treated with HRT – that's a clear contraindication for someone with active breast cancer or who is in remission, as you've highlighted in the discussion. More interesting are the ADHD/autism findings (engagement with primary care as I've raised?) and dementia/learning difficulties/Down's syndrome (reduced capability to share that menopause may have started?).<br>Discussion<br>Page 10 line 28: I don't think this is the first study to describe prescribing rates of HRT in England at an individual level – Burkard et al. have done it in 2019 using CPRD data. Not to say that what has been done here isn't an important contribution to a very small field, just might be worth referencing these colleagues.

	Page 11 line 19: the Health Survey for England finding where differences attenuated following adjustment for socioeconomic factors – I think this is important in your study also given the striking ethnic inequalities across the most deprived Townsend group. Do you think that a Townsend quintile is an adequate capture of SEP? If not (which is what I think – these quintiles are not sufficient to believe SEP is fully accounted for following adjustment), this needs mentioning in the limitations.<br>Page 12 line 3: agree regarding the point about the unexpected social trend of transdermal patch prescribing against CV risk. A potentially useful sensitivity analysis to support this claim would be to show indications for transdermal patches (like CVD) by Townsend quintile. This would cement our belief that something else is driving this unrelated to contraindication burden that differs by SEP.<br>Page 13 line 22: be specific about the type of qualitative work that would be useful in this space. Understanding why different women are and are not taking HRT would provide a lot of useful context to the findings of this descriptive study.<br>Box 1<br>Intrauterine device not inter-uterine; define acronym as used below in Table 1.<br>Box 2<br>How were the comorbidities identified/chosen for investigation?<br>Table 1<br>I think a footnote could be useful explaining that the HRT prescription rows are not mutually exclusive from one another.<br>I don't think row %'s are the most useful here – as the reader, I want to see whether the distribution of categories among HRT/no HRT differs. It's obvious that the no HRT characteristics will make up the bulk because it's the largest group. Like in Table 3 where column %'s are reported – it's much more useful in aiding comparison between groups.<br>Table 3<br>I'm wondering if the proportions of stratification on type of HRT would be more interesting using total N of those with an HRT prescription as the denominator rather than out of the full cohort. This would help me get a better feel of the distribution of type within the exposed group (we're not interested in the unexposed here because they didn't use HRT so shouldn't make up most of the denominator). Same then goes for the body of text where these results are reported.<br>Table 6<br>Cardiovascular comorbidities have a lower likelihood of prescribing makes sense from a stroke/DVT risk perspective. Interesting though opposite for myocarditis that could be worth discussing in the main text? With the caveat that the numbers are small.<br>Serious mental illness makes sense from a psychosis perspective but interesting.<br>ADHD and autism findings are interesting.<br>Learning difficulties/Down's syndrome/dementia – communicating that menopause has started?<br>Table S2<br>What is the 1 and 2 referring to? Number of prescriptions like S3? Edit to 2+ like below.<br>Table S4<br>Something weird has happened with the hyphens in the IQR.<br>Table S5<br>Need to clarify that you're not adjusting for the exposure in each of the analyses, i.e., in the multivariable Cox model of age on HRT prescription, you're not adjusting for age (or chouldn't be as this is

	the exposure). Same applies to other tables where adjustments for other “exposures” have been performed.<br>Table S7<br>Really strong social patterning for transdermal oestrogen that is not observed in oral. The opposite being true for regional differences. Interesting as you have flagged in the main text.<br>Table S8<br>Not exactly sure what the exposure/outcomes are here?

**REVIEWER NAME**	Harron, Katie
**REVIEWER AFFILIATION**	
**REVIEWER CONFLICT OF INTEREST**	None
**AI DISCLOSURE**	No
**DATE REVIEW RETURNED**	14-Mar-2025

**GENERAL COMMENTS**	This is an interesting study and the authors present comprehensive results on the association between HRT prescribing and ethnicity and deprivation. Whilst I appreciate that lots of different analyses have been undertaken, I think that the focus on looking at different formulations could be moved to the appendix as I’m not sure this fits well with the main message of the paper which seem to be around inequalities in any prescribing. I think a more valuable analysis would be to look at how much of the association between deprivation (or ethnicity) and prescriptions is explained by or remains after adjusting for comorbidities, smoking and BMI. I believe these data are available within the dataset.<br><br>What this study adds section: “These disparities persist even after adjusting for sociodemographic factors, suggesting potential inequities access and different levels of HRT uptake that warrant exploration.” There is a word missing here. The previous paragraph was referring to deprivation, so by “sociodemographic factors”, does this mean ethnicity? I think this point could be reworded though – even if the disparities were explained by ethnicity, wouldn’t those differences still warrant more in depth exploration?<br><br>The introduction states that The NICE menopause guidance is currently being updated and is scheduled for publication in November 2024. Can this be updated?<br><br>Please justify why you specifically selected women aged 40-60.<br><br>Justification should be given early on for why you focused on those with two or more HRT prescriptions.<br><br>“being of lower socioeconomic bracket” odd wording here. Living in a more deprived neighbourhood? Suggest reword.<br><br>The second paragraph of results states twice that 379,911 (19.2%) of women were receiving two or more prescriptions. Is this an error, is there supposed to be a different number in each of these sentences?<br><br>“When we restricted to prescriptions from 2016” – please state the N included from 2016 onwards.<br><br>“the trend looked less significant” – this isn’t clear, please reword

	(something is either statistically significant according to whatever definition you are using, or it isn't).<br><br>Treatment of missing data needs to be described in the methods section.<br><br>Table 1 and 2 could be combined.<br><br>Please provide overall p-values for the association between e.g. ethnicity and HRT, rather than p-values for the individual levels of the variable.<br><br>Box 2 – is this needed given these conditions are all listed elsewhere e.g. in table 6?<br><br>There is a very large odds ratio for HRT for those with ADHD which stands out amongst other conditions, can this be explained? I think the row percentages would be more helpful here – to show for example that almost 50% of women with ADHD have a HRT prescription.<br><br>Figure 1 – the x axis needs more detail in the numeric labelling.

VERSION 1 – AUTHOR RESPONSE

**Reviewer: 1**<br>**Prof. Martha Hickey, University of Melbourne**<br>**Comments to the Author**	
Thanks for asking me to review this interesting manuscript. This is a large, GP records based study (not a cohort study as such) of HRT prescribing in the UK, broken down by age, ethnicity and social deprivation, based on postcode.	Thank you for your review.
Based on the title and abstract, I was confused about what this study is about. The title talks about inequalities in prescribing of HRT but the abstract talks about inequalities in uptake. These are not the same thing and the authors should clarify the focus of their study.	Thank you for spotting this.<br><br>We have removed the word "uptake" and have revised the objectives in the abstract, with now reads: "Objectives: To quantify hormone replacement therapy (HRT) prescribing in women aged 40-60 by types of HRT and duration of use and identify sociodemographic factors associated with receiving an HRT prescription."
Why was the age range 40-60 used? The average age at onset of the menopause transition is 47 years, so prior to this most women will be premenopausal (using STRAW criteria). Age at menopause may be younger in	The lower age of the cohort at 40 meets the NICE definition of menopause and early menopause and our PPI group felt that it was important to capture younger ages as some Black and Asian populations may experience

UK minority groups might be younger, but this is not yet known.	menopause earlier than white women. Women who are 40 at start of follow-up will be 50 by the end of the study, when they may have considered HRT.<br><br>The upper age was selected as 60 years as women can experience symptoms for many years after menopause. We recognise that some of our cohort will be 70 at the end of follow-up.
The observation that socially advantaged white women are more likely to take HRT is not new and is consistent with large observational studies such as NHS. However, I think this information is new (but not unexpected) for the UK.<br><br>The conclusion that HRT prescribing represents inequalities is a bit presumptuous. What they have observed is a difference in reporting rates by economic deprivation and minority status. They don't know the reasons for this. These groups may have different menopausal symptoms and/or cultural or personal reasons why they don't wish to take HRT. Based on published observations from LMIC, this might well be the case. Similarly, the evidence shows that white women are more likely to have vasomotor symptoms (the primary indication for HRT) and to ask for treatment. Language is important here. The authors imply that greater prescribing of HRT to these groups would be 1) wanted and 2) beneficial.<br>So, why the findings may suggest "health inequities" there are other interpretations which should be acknowleged<br>As recognised by NICE 2024, there is a substantial gap in understanding about the risks and benefits of HRT in minority women. There is also a gap in understanding about the needs of this group for menopause care. This should be highlighted.	Thank you for these comments. We have revised the conclusion in the abstract and this now reads:<br><br>“This work has identified important differences in HRT prescribing in England based on ethnicity, socioeconomic status, and geographic location. White women and those in more affluent neighbourhoods were more likely to receive HRT than non-white women and those in more deprived areas. These findings suggest potential inequities which require further exploration.”
Specific issues<br>In the introduction the authors quote the 2002 MacLennan Cochrane review to support that "HRT treats symptoms of the menopause". As indicated in the Cochrane review title, HRT is effective for vasomotor symptoms. Suggesting efficacy for the vague "symptoms of the menopause" is potentially misleading, given the	We have revised this to specify vasomotor symptoms only in the introduction.

multiple "symptoms" this might mean. Please revise.<br><br>Note that NICE guidelines on menopause are now updated<br><br>Evidence from SWAN about racial differences in vasomotor symptoms has been extremely valuable. However, it is not appropriate to generalise from these AA women to minority groups in the UK - at least not without evidence	We refer to the first NICE menopause guidance in 2015 and explain that:<br><br>"The NICE menopause guidance was updated in 2024(8) and included a discussion aid on HRT and the likelihood of some medical conditions to support GPs.(9)"<br><br>We have clarified the point about ethnic groups in the UK. The revised text now reads:<br><br>"The demographic makeup of different ethnicities in the UK compared with the USA means that generalisability is not possible, but it is extremely likely that differences by ethnicity will persist."
Regarding the type of HRT, NICE recommend that transdermal preparations are used in those with risk factors for potential risks of HRT such as VTE and stroke. If these risk factors are more prevalent in disadvantaged groups then use of TD preparations should be promoted.<br><br>Qualitative studies with prescribers may help to determine why this does not happen.	We have already included the following text in the introduction.<br><br>As the burden of cardiovascular disease is higher in deprived areas compared with affluent (23) and transdermal preparations are recommended for women with a higher risk of thromboembolism or stroke,(8)<br><br>We have added the suggested sentence about future qualitative studies to the conclusions:<br><br>"This pattern, which has been previously observed in an analysis of UK prescribing data,(21) is unexpected as those in areas of high social deprivation have higher rates of cardiovascular disease(23) and transdermal HRT preparations are recommended for those with higher cardiovascular risk.(37, 38) Qualitative studies with prescribers may help to determine why this does not happen."
I found the comment about "not wanting additional pills" a bit unconvincing (no ref). Many combined TD products require twice weekly changing which may be more annoying than a daily pill!<br><br>statements about HRT inequalities should not quote evidence from the all party parliamentary group. This group was funded by a	We have removed the sentence about additional pills

pharmaceutical group who manufacture HRT. Suggest sticking with peer reviewed pubs.<br><br>Specifically, I was not convinced by the suggestion that UK women with the most severe vasomotor symptoms are not getting access to HRT. This seems speculative.<br><br>I am not qualified to discuss the statistical methods	We have removed the reference to the all party parliamentary report and text summarising it from the manuscript.<br><br>We have changed the wording here to say “It remains uncertain whether the women experiencing the most severe symptoms are those who are prescribed HRT and whether there may be unmet need in some communities, specifically amongst women from more disadvantaged areas or women from minority ethnic backgrounds who may have less access to treatment.”<br><br>Additionally, we have added:<br><br>“Women’s experiences of menopause and accessing care as well as how perceptions and approaches may be shaped by cultural norms, could be explored using in-depth semi-structured interviews and focus groups with women experiencing menopause and the health care professionals caring for them.”
Results<br>I was interested to see that the mean age at HRT prescription was 49 years. Since the mean age at menopause in the UK is 51 years, this suggests that some women are starting HRT in the menopause transition. The authors should discuss that the safety and efficacy of HRT in this population is very poorly understood, since almost all clinical trials are restricted to postmenopausal women.<br><br>The study contains interesting (and novel) information about discontinuation of HRT by ethnicity. They report >2 fold % of minority women who had only one prescription of HRT vs white women. Why might this be? This seems very important for the message. Is the issue that these women want/need HRT and are not offered it, are they offered it then discontinue after one prescription or do they decline it? The	We have added the following to the discussion to highlight this:<br><br>“We note that the average age of HRT prescription is 49 years. As the average age of menopause in the UK is 51(52) this may suggest that more women are being prescribed HRT in the menopause transition or perimenopause. This is an interesting finding given the limited trial evidence on HRT efficacy in this group. Alternatively, it is possible that women with earlier age at menopause are more likely to be prescribed HRT.”<br><br>We have added this point to the discussion as follows:<br><br>“The proportion of women who only received a single prescription relative to those who went on to receive a second prescription was higher in those from minority ethnic groups compared with

important data in this study need this information because they can be used to inform practice.	white populations. We do not know the reasons for this without further investigation, but these may be women who changed their mind about taking HRT, or wanted to accept the prescription to please their doctor, or who tried it and did not find it helpful enough."
**Reviewer: 2**<br>**Dr. Florence Martin, University of Bristol**<br>**Comments to the Author**	
Thank you to the authors for this interesting work. It is an important study in an underrepresented field, describing HRT utilisation in England using QResearch primary care data (EMIS). Authors have descriptively explored ethnic and socioeconomic differences, as well as prescribing among different comorbidities and characteristics. I have included some suggestions and questions below. I have numbered the pages as reported on the manuscript, not of the proof in its entirety.	Thank you for reviewing our manuscript and for your helpful comments
Abstract	
Page 2 line 27: not sure what the breakdown of 8.3% and 5.0% is of those receiving HRT? But then what makes up the other 87%. If proportion of the full cohort, what is the other 5.9% if not oral or transdermal. I comment on this later on, but I think when breaking down the types of HRT, the proportions should be out of those taking HRT (as opposed to the full cohort as the denominator).	We have now changed this to the proportion of those who received HRT, so these are 43.3% receiving oral and 26.3% receiving transdermal. For the remainder it was unclear, but we do not have word count in the abstract to explain this. However, we have explained this in the main text.
Page 2 line 44: seems like HRT prescribing is systematically lower than I would have expected, which is an important finding too, alongside the inequalities!	HRT prescribing was taken from 2013-2023 over this period there has been a large rise in HRT prescriptions as shown until 2021 and as stated it has increased further still. However, as the rate was so low initially this would have affected the overall percentage. Ref Alsugeir 2022, BJGP **DOI:** https://doi.org/10.3399/BJGPO.2022.0126
**Introduction**<br>Throughout: be consistent with capitalising 'Black' when referring to ethnicity	Thank you, we have corrected this
Are there any other studies that report the prevalence of HRT use in the UK? This might be a nice addition to the introduction (e.g., Burkard et al, 2019?) to situate your subsequent findings of HRT use among 40-60 year olds in QResearch, before exploration of relevant	We have added this text and references to the introduction:

subgroups.<br>Something about the risk landscape (DVT, ischaemic stroke, cancer) would be useful here I think, given that you discuss that inequalities in certain comorbidities might drive differences in HRT prescribing among certain groups.	"Over the years, HRT prescribing rates have varied, with rates dropping(3, 4) following the Women's Health Initiative publication in 2002 reporting adverse health outcomes,(5) and more recently, HRT prescribing has increased hugely.(6, 7)"<br><br>3. Iversen L, Delaney EK, Hannaford PC, Black C. Menopause-related workload in general practice 1996-2005: a retrospective study in the UK. Fam Pract. 2010;27(5):499-506.<br><br>4. Burkard T, Moser M, Rauch M, Jick SS, Meier CR. Utilization pattern of hormone therapy in UK general practice between 1996 and 2015: a descriptive study. Menopause. 2019;26(7):741-9.<br><br>6. NHS. NHSBSA Statistics and Data Science - Hormone Replacement Therapy - England - April 2015 to June 2023 2023 [Available from: https://www.nhsbsa.nhs.uk/statistical-collections/hormone-replacement-therapy-england/hormone-replacement-therapy-england-april-2015-june-2023.<br><br>7. Alsugeir D, Wei L, Adesuyan M, Cook S, Panay N, Brauer R. Hormone replacement therapy prescribing in menopausal women in the UK: a descriptive study. BJGP Open. 2022.
Methods	
Page 5 line 12: if space, I would benefit from a little bit more information about QResearch (namely, where the data is coming from – EMIS) so readers can place the research within their existing understanding of other primary care data repositories like CPRD, rather than having to follow the link, like I did.	Thank you for this suggestion, we have added "using Optum (formerly EMIS Health)" to the end of the sentence.
Page 5 line 29: is Townsend deprivation score individual-level or practice-level? Either way, it is a relatively blunt tool to capture SEP. Of course it's all we have in English EHRs, but important to comment on as I have raised in the discussion.	I have added "based on individual level postcode" to the methods.<br><br>The limitations of the Townsend score have been added to the discussion (detailed below in our response)

Page 5 line 38: given that Mirena is a brand name, it might be more appropriate to use the generic “intrauterine progesterone-only device” or words to that effect.	Thank you we have changed the word Mirena to “intrauterine progesterone-only device (52mg Levonorgestrel IUD)”
How were conflicting ethnicity records handled? Presumably there were people in the cohort who have ethnicity recorded more than and in multiple sources (EMIS and HES). In my experience these don’t always agree – was this dealt with at all?	Ethnicity data are self-reported and pulled from GP records (85% of the records). Where there were missing data, these were supplemented with data recorded on HES records (around 10% of the records).<br><br>I have changed the wording to reflect this as follows: “as recorded on GP records using hospital records to supplement missing data”
As I comment on in the results, I’m unsure of the benefit of having both logistic and Cox, particularly where time is important (those with longer follow-up have more chance to be prescribed HRT multiple times and thus be considered to have experienced the outcome under study).	We have changed the primary analysis to Cox regression as suggested and moved the logistic regression to the supplementary materials as this was a pre-specified analysis.<br><br>We have also changed the analysis of prescriptions from 2016, interaction tests and regression of comorbidity data to a Cox regression.<br><br>Other exploratory analyses have been left as logistic regression including the comparison of oral and transdermal formulations.
Results	
Page 6 line 56-57: when you say “…received two or more HRT prescriptions at any time…” that is presumably referring to during follow-up right? From the point at which they became eligible (aged ≥40 at the start of 2013 or from the point they turned 40 after the start of the study)? Might be worth clarifying either here or in the methods.	Because some women were 60 years at the start of the study follow-up, we used HRT prescriptions at any time, including prior to study follow-up which was specified in the second paragraph of the methods “HRT uptake was defined throughout as those who had at least two prescriptions of the same type of HRT at any time prior to study entry or during follow-up”<br><br>We have also only looked at prescriptions after 2015 in comparison.
Page 6 line 56: very similar to the sentence starting line 58 – consider consolidating?	Thank you for pointing this out. We have now combined these two sentences.

Page 7 line 3: I'm struggling to follow these proportions in terms of what their denominator is. Is the denominator the full cohort or those who received ≥2 HRT prescriptions? My vote is for the latter (discussed below). Also, if the groups aren't mutually exclusive, consider informing the reader of this so it is easy to follow.	We have reworded this section to make the denominator in the percentages those who had ≥2 HRT prescriptions and make it clear that they are not mutually exclusive:<br><br>"Of those who were prescribed HRT, 237,205 (62.4%) received oestrogen and progestogens in a single prescription, with 164,553 (43.3%) of the women prescribed HRT receiving oral formulations and 99,852 (26.3%) receiving transdermal oestrogen-progestogen combined formulations. Some women (8.0%) received prescriptions for both oral and transdermal formulations. 192,484 (50.7%) women received a prescription for an oestrogen-only formulation. Of these, a higher proportion of women received transdermal oestrogens (39.9% of all women on HRT) compared with oral oestrogen (15.1% of all women on HRT). Some women (4.8%) received prescriptions for both oral and transdermal formulations."
Page 7 line 11: the latter part of this paragraph feels like a comparison between 2013-2023 and 2016-2023. To further aid readability, it might be useful to mirror these parts of the paragraph so that the numbers are easier to compare.	As suggested, we have mirrored the wording for this section and changed the denominator in all cases to those with an HRT prescription.<br><br>This section now reads:<br><br>"When we restricted to prescriptions from 2016 (n=205,909), 125,014 (60.7%) received a combined oestrogen and progestogen in a single prescription, with 31% of those with an HRT prescription receiving an oral combined formulation and 40% a transdermal combined oestrogen-progestogen combined formulation. Some women (6.9%) received prescriptions for both oral and transdermal formulations. In the cohort, 121,285 (58.9%) women received a prescription for an oestrogen-only formulation after 2016 and a higher proportion of women received transdermal oestrogens (56.4% of all women on HRT) compared with oral oestrogen (6.7% of all women on HRT). Some women (2.0%) received prescriptions for both oral and transdermal formulations."
Page 8 line 7: interesting that prevalence of prescribing among Caribbean women is less than half but their duration of prescribing is significantly higher? I think more discussion	We have added the following to the discussion:<br><br>"Our work has established that Black women are less likely to receive HRT compared with

around why this might be useful as I think this finding is interesting.	white women but mean duration of oestrogen formulations in Caribbean women was higher than other ethnic groups, which suggests long symptom duration in this UK population may be consistent with US data in African Americans.”
Page 8 lines 13-: the very low ORs attenuate in the Cox models so are they strongly driven by differential follow-up? Proportional hazards probably violated as the “risk” of starting HRT changes over time (most notably post 2015 guidance), which could do with a note in the limitations (similarly commenting on the lack of consideration of length of follow-up in the logistic regression models).<br><br>Are there changes in proportions of the exposure groups (ethnicity/deprivation etc.) over time which could be contributing to this? Similarly is length of follow-up stratified by exposure groups reported in the supplement? I might have missed it but if not there, that could be helpful in interpreting this.	The Cox proportional hazards model has now been used as the main analysis as suggested to allow for different lengths of follow up in the cohort. We have checked the proportional hazards using log-minus-log plots and found no evidence of having violated proportional hazards assumptions.<br><br>We have added length of follow-up to Table 1 and included the mean (SD) follow-up time in years by ethnicity, quintile of deprivation and region below:<br>White, 6.7 (3.5)<br>Indian, 6.2 (3.5)<br>Pakistani, 6.4 (3.5)<br>Bangladeshi, 6.5 (3.6)<br>Other Asian, 5.6 (3.5)<br>Caribbean, 7.0 (3.4)<br>Black African, 5.6 (3.5)<br>Chinese, 5.3 (3.6)<br>Other, 5.5 (3.5)<br>Not recorded, 6.0 (3.6)<br><br>Quintile of Townsend deprivation N (column %),<br>1 – most affluent, 6.9 (3.4)<br>2, 6.6 (3.5)<br>3, 6.4 (3.5)<br>4, 6.2 (3.5)<br>5 – most deprived, 6.1 (3.6)<br>Not recorded, 4.6 (3.5)

	Region N (column %),<br>East Midlands, 7.4 (3.5)<br>East of England, 6.7 (3.4)<br>London, 5.8 (3.6)<br>North East, 8.1 (3.3)<br>North West, 7.0 (3.4)<br>South Central, 6.7 (3.4)<br>South East, 5.7 (3.4)<br>South West, 6.8 (3.5)<br>West Midlands, 6.7 (3.4)<br>Yorkshire & Humber, 7.6 (3.4)
As I raised in the methods, I'm not sure what the benefit of having both logistic regression and Cox is. I would be inclined to say that Cox is more favourable here because it accounts for time in a way that logistic regression doesn't, and you get the same conclusion so seems redundant to have both. If you do really want logistic regression, I would switch them and have Cox in the main manuscript.	We have changed our main analysis to a Cox proportional hazards model. The logistic regression findings are now in the supplementary data
Page 8 line 47-: what could be driving differences in formulation across subgroups? Weird that the reasons for having a transdermal patch (comorbidities etc.) are higher in the more deprived group but transdermal patch use is lower… But attenuated when restricting to post-2015 guidance. Why are these data not included?<br>Do you have hypotheses regarding differential prescribing? Prescriber sex, education, engagement with women's health? I think the discussion could benefit from a bit more about this.	We have now included the data for prescriptions from 2016 in the table (now supplementary Table 10 a and b).<br><br>The odds ratios still indicate there is a trend for lower prescribing of transdermal preparations with increasing deprivation but this is no longer statistically significant which may be due to small numbers.<br><br>We have added the following text:<br><br>"Restricting these analyses to prescriptions after 1st Jan 2016 showed that the trends to lower prescribing in the most deprived quintiles remained but were no longer significant for either oestrogen or combination formulations (Supplementary Table 10)."

Page 9 line 23-: could engagement with primary care services driving some of the differing proportions of comorbidities among those prescribed HRT compared with those who were not? Some of the findings are a useful sense check but don't necessarily need flagging in the text (here or in the discussion at length); for example, it's expected that those with breast cancer aren't being treated with HRT – that's a clear contraindication for someone with active breast cancer or who is in remission, as you've highlighted in the discussion. More interesting are the ADHD/autism findings (engagement with primary care as I've raised?) and dementia/learning difficulties/Down's syndrome (reduced capability to share that menopause may have started?).	This analysis cannot determine the reasons for the higher HRT prescribing in those with ADHD.<br><br>We have added the following to the discussion:<br><br>"It is unclear why those with ADHD had higher prescribing rates, but it is possible that menopause exacerbates ADHD symptoms,(46) or may be that these groups already have an active engagement with healthcare."
Discussion	
Page 10 line 28: I don't think this is the first study to describe prescribing rates of HRT in England at an individual level – Burkard et al. have done it in 2019 using CPRD data. Not to say that what has been done here isn't an important contribution to a very small field, just might be worth referencing these colleagues.	We have re-worded this sentence and it now reads: "This is the first study to quantify ethnic differences in HRT prescribing at an individual level across nine ethnic groups in English primary care data."
Page 11 line 19: the Health Survey for England finding where differences attenuated following adjustment for socioeconomic factors – I think this is important in your study also given the striking ethnic inequalities across the most deprived Townsend group. Do you think that a Townsend quintile is an adequate capture of SEP? If not (which is what I think – these quintiles are not sufficient to believe SEP is fully accounted for following adjustment), this needs mentioning in the limitations.	The Townsend Deprivation Index is a measure of material deprivation incorporating unemployment, car ownership, home ownership and household overcrowding across neighbourhoods. The Townsend score can be calculated from individual postcode and has the advantage over Index of Multiple Deprivation (IMDS) in that it does not include a measure of health which would confound the analyses. It is also evaluated at output area which is more granular than the IMDS and hence potentially more accurate for analyses.<br><br>It has the limitation that car ownership may not be the best proxy for including in a measure of material deprivation, particularly in urban areas and this has been added to the limitations:<br><br>"The Townsend Deprivation Index is an area measure of material deprivation based on four variables, including car ownership. Car ownership may not be the best measure of deprivation, particularly in urban areas and the Index may not have accurately reflected actual

	individual level deprivation, which is a limitation of our analyses.”
Page 12 line 3: agree regarding the point about the unexpected social trend of transdermal patch prescribing against CV risk. A potentially useful sensitivity analysis to support this claim would be to show indications for transdermal patches (like CVD) by Townsend quintile. This would cement our belief that something else is driving this unrelated to contraindication burden that differs by SEP.	We have done this sensitivity analysis and have included the results in Table 11 alongside the analysis for any HRT adjusting for smoking, BMI and cardiovascular comorbidities.<br><br>We found that when we restrict to those who had prescriptions of transdermal preparations only without considering any other HRT formulation, regardless of whether we adjust for other covariates such as smoking status, BMI and cardiovascular comorbidities, the hazard ratio for those in the most deprived quintile compared to the most affluent quintile was 1.07 (1.07, 1.08). Because we have ignored all other HRT prescriptions in this analysis, and it was not pre-specified, we have not added this to the manuscript, but can include it if the reviewer and editor think it is important.
Page 13 line 22: be specific about the type of qualitative work that would be useful in this space. Understanding why different women are and are not taking HRT would provide a lot of useful context to the findings of this descriptive study.	Understanding women’s preferences, and what interventions are required to address inequities to menopause care, is complex and outside the scope of this project but requires further exploration using qualitative methodologies. Women’s experiences of menopause and accessing care as well as how perceptions and approaches may be shaped by cultural norms, could be explored using in-depth semi-structured interviews and focus groups with women experiencing menopause and the health care professionals caring for them.
Box 1<br>Intrauterine device not inter-uterine; define acronym as used below in Table 1.	This has been changed and acronym defined
Box 2<br>How were the comorbidities identified/chosen for investigation?	These were selected using a combination of expert opinion and data availability.
Table 1<br>I think a footnote could be useful explaining that the HRT prescription rows are not mutually exclusive from one another.<br>I don’t think row %’s are the most useful here – as the reader, I want to see whether the distribution of categories among HRT/no HRT differs. It’s obvious that the no HRT	We have added a footnote to Table 1 to indicate that the rows are not mutually exclusive and changed it to report column % as the reviewer suggests

characteristics will make up the bulk because it's the largest group. Like in Table 3 where column %'s are reported – it's much more useful in aiding comparison between groups.	
Table 2<br>I'm wondering if the proportions of stratification on type of HRT would be more interesting using total N of those with an HRT prescription as the denominator rather than out of the full cohort. This would help me get a better feel of the distribution of type within the exposed group (we're not interested in the unexposed here because they didn't use HRT so shouldn't make up most of the denominator). Same then goes for the body of text where these results are reported.	Table 3 & Table 4 are now in the Supplementary data, supplementary Table 2 and 4 respectively<br><br>The denominator for individual HRT formulations has now been changed to those with HRT prescriptions Table 1 (Previously Table 2) and in the associated text.
Table 6<br>Cardiovascular comorbidities have a lower likelihood of prescribing makes sense from a stroke/DVT risk perspective. Interesting though opposite for myocarditis that could be worth discussing in the main text? With the caveat that the numbers are small.<br>Serious mental illness makes sense from a psychosis perspective but interesting.<br>ADHD and autism findings are interesting.<br>Learning difficulties/Down's syndrome/dementia – communicating that menopause has started?	We have added some more text to the discussion:<br><br>"It is unclear why those with ADHD had higher prescribing rates, but it is possible that menopause exacerbates ADHD symptoms,(49) or may be that these groups already an active engagement with healthcare."
Table S2<br>What is the 1 and 2 referring to? Number of prescriptions like S3? Edit to 2+ like below.	Table S2 (now table S3) has been edited as suggested
Table S4<br>Something weird has happened with the hyphens in the IQR.	The hyphens have been re-added to Table S4 (now table S6)
Table S5<br>Need to clarify that you're not adjusting for the exposure in each of the analyses, i.e., in the multivariable Cox model of age on HRT prescription, you're not adjusting for age (or chouldn't be as this is the exposure). Same applies to other tables where adjustments for other "exposures" have been performed.	Tables have been reconfigured and age is not included in any of the Cox models or tables to avoid confusion.
Table S7<br>Really strong social patterning for transdermal oestrogen that is not observed in oral. The opposite being true for regional differences. Interesting as you have flagged in the main text.	Now Table S9<br><br>We agree, which we explore further in Table S10 (formerly S8)

Table S8<br><br>Not exactly sure what the exposure/outcomes are here?	This is now Table S10.<br><br>Outcome is receiving transdermal preparation compared to oral.<br><br>Exposures are ethnicity, deprivation score and region<br><br>We have added clearer labels to the table that this is transdermal compared to oral preparations.
**Reviewer: 3**<br>**Prof. Katie Harron**	
Comments to the Author<br>This is an interesting study and the authors present comprehensive results on the association between HRT prescribing and ethnicity and deprivation. Whilst I appreciate that lots of different analyses have been undertaken, I think that the focus on looking at different formulations could be moved to the appendix as I'm not sure this fits well with the main message of the paper which seem to be around inequalities in any prescribing.	Thank you for your helpful comments.<br><br>We have moved the two tables (formerly tables 3 and 4,) reporting the different HRT formulations to the appendix (now tables S2 and S4 respectively)
I think a more valuable analysis would be to look at how much of the association between deprivation (or ethnicity) and prescriptions is explained by or remains after adjusting for comorbidities, smoking and BMI. I believe these data are available within the dataset.	We cannot adjust for every comorbidity, but instead have adjusted for cardiovascular comorbidities, either prior to first HRT prescription in those with HRT or at baseline for those without a prescription.<br><br>After adjusting for existing CVD, most recent smoking status (current or not current) and BMI category (underweight, normal, overweight and obese), Cox regression still revealed differences by ethnicity and deprivation. Hazard ratio for those in the most deprived quintile of deprivation was 0.92 (0.91-0.93) compared to those in the most affluent quintile.<br><br>We have added this as a sensitivity analysis and included the following text: "Supplementary Table 11 shows Cox regression for HRT prescribing after adjusting for BMI, smoking status and existing cardiovascular disease. Adjusting for these factors does not explain the differences in prescribing with hazard ratios for Chinese and Other Asian ethnic groups of 0.80

	(0.79, 0.82) and 0.82 (0.81, 0.83) respectively and hazard ratio of 0.92 (0.91, 0.92) for the most deprived quintile compared to the most affluent.”
What this study adds section: “These disparities persist even after adjusting for sociodemographic factors, suggesting potential inequities access and different levels of HRT uptake that warrant exploration.” There is a word missing here. The previous paragraph was referring to deprivation, so by “sociodemographic factors”, does this mean ethnicity? I think this point could be reworded though – even if the disparities were explained by ethnicity, wouldn’t those differences still warrant more in depth exploration?	We have reworded this section to make it clearer. It now reads:<br><br>“These disparities, which persist even after adjusting for other sociodemographic factors suggest potential inequities in access and different levels of HRT uptake that warrant exploration.”
The introduction states that The NICE menopause guidance is currently being updated and is scheduled for publication in November 2024. Can this be updated?	We have amended this with the latest guidance and the associated discussion aid. This now reads:<br><br>“The NICE menopause guidance was updated in 2024(8) and published a discussion aid on HRT and the likelihood of some medical conditions, to support GPs.(9)”
Please justify why you specifically selected women aged 40-60.	The lower age of the cohort at 40 meets the NICE definition of menopause and early menopause.(1) We have decided not to include a population younger than 40 as this would meet the definition of primary ovarian insufficiency which is a different population and research question.(1) The upper age was selected as 60 years as women can experience menopausal symptoms for many years. This is supported by the reports that vasomotor symptoms can continue for up to 10 years after experiencing menopause.(2 3) Over 10 years of follow-up, the population will be up to age 70 years at the end of the study. There was therefore no justification for increasing the upper age as initiation of HRT would be less likely.<br><br>(1) NICE. HRT and the likelihood of some medical conditions A discussion aid for healthcare professionals and patients2024 7 Jan 2025. Available from: https://www.nice.org.uk/guidance/ng23/resources/incidence-of-medical-

	conditions-with-and-without-hrt-a-discussion-aid-pdf-13553199901.<br>(2) Chung HF, Pandeya N, Dobson AJ, et al. The role of sleep difficulties in the vasomotor menopausal symptoms and depressed mood relationships: an international pooled analysis of eight studies in the InterLACE consortium. *Psychol Med* 2018;48(15):2550-61. doi: 10.1017/s0033291718000168 [published Online First: 2018/02/13]<br>(3) Hickey M, Szabo RA, Hunter MS. Non-hormonal treatments for menopausal symptoms. *BMJ (Clinical research ed)* 2017;359:j5101.<br><br>We have included the following in the methods to explain this along with the references:<br><br>“..... as this is the age range that they are most likely to experience menopause and its symptoms.(8, 25)”
Justification should be given early on for why you focused on those with two or more HRT prescriptions.	We have added this to the methods defining HRT uptake as two or more prescriptions: “to allow for those not collecting or taking their medication appropriately.”
“being of lower socioeconomic bracket” odd wording here. Living in a more deprived neighbourhood? Suggest reword.	This has been changed as suggested.
The second paragraph of results states twice that 379,911 (19.2%) of women were receiving two or more prescriptions. Is this an error, is there supposed to be a different number in each of these sentences?	Thank you for noticing this, we have removed the second sentence with duplication
“When we restricted to prescriptions from 2016” – please state the N included from 2016 onwards.	We have added the numbers who were prescribed HRT since publication of the 2015 NICE guidelines (n=205,909)
“the trend looked less significant” – this isn’t clear, please reword (something is either statistically significant according to whatever definition you are using, or it isn’t).	We have reworded this and it now reads: “..but the trend was not statistically significant in some non-white ethnic groups, in particular Bangladeshi and Caribbean groups.”
Treatment of missing data needs to be described in the methods section.	We have added a sentence to the methods: “A missing indicator variable “Not reported” was generated for those with missing data for ethnicity and deprivation and used for descriptive and statistical analyses.”
Table 1 and 2 could be combined.	We have combined Tables 1 and 2, which we agree results in less repetition. In combining the

	two tables and following comments from reviewer 2, we have changed the percentage to column percent throughout.
Please provide overall p-values for the association between e.g. ethnicity and HRT, rather than p-values for the individual levels of the variable.	We have simplified the p-values, to only report one per group
Box 2 – is this needed given these conditions are all listed elsewhere e.g. in table 6?	We have removed Box 2 listing each of the comorbidities.
There is a very large odds ratio for HRT for those with ADHD which stands out amongst other conditions, can this be explained? I think the row percentages would be more helpful here – to show for example that almost 50% of women with ADHD have a HRT prescription.	This analysis cannot determine the reasons for the higher HRT prescribing in those with ADHD, but could be a combination of factors from this groups potentially having more severe symptoms, or may be that there is already an active engagement with healthcare.<br><br>We have added the following to the discussion:<br><br>"It is unclear why those with ADHD had higher prescribing rates, but it is possible that menopause exacerbates ADHD symptoms,(46) or may be that these groups already an active engagement with healthcare."
Figure 1 – the x axis needs more detail in the numeric labelling.	As well as replacing this plot with the equivalent for the Cox regression, we have updated Figure 1 to give more labels on the x-axis

VERSION 2 – REVIEW

**REVIEWER NAME**	Martin, Florence
**REVIEWER AFFILIATION**	University of Bristol, MRC Integrative Epidemiology Unit
**REVIEWER CONFLICT OF INTEREST**	None
**AI DISCLOSURE**	No
**DATE REVIEW RETURNED**	05-Jul-2025

**GENERAL COMMENTS**	Thank you to the authors for their thoughtful responses and amendments to their important paper. Only minor suggestions to help with word count, undefined acronyms noted, and a query regarding table 1.<br><br>Abstract<br><br>Standard deviation acronym isn't defined – maybe it's not needed at all and mean is sufficient for the purpose of the abstract?

	Confidence interval acronym isn't defined.<br><br>Results<br><br>To save words, I wonder if you could acronymise hazard ratio to HR (I know it's similar to HRT but I know you're short on word count!).<br><br>I also don't think p-values are necessary, particularly as you want to get some words back. Fine in the abstract when you're not reporting confidence intervals, but where we can see confidence intervals not crossing one, p-values are redundant.<br><br>On page 59, standard deviation is not defined at first use in the main text.<br><br>On page 62 of the proof, none of the new comorbidity-related acronyms (TIA, CVD, VTE etc.) have been spelt out as far as I can see (sorry not helpful for word count!).<br><br>On page 62 of the proof, odds ratio and confidence interval acronym isn't defined at first use.<br><br>Consistency with how confidence intervals are reported in the text (x, x) compared to (x-x) is important (as there's a mix on page 61 and 62 for example); choosing the latter format would help save words.<br><br>Table 1<br><br>In the age section, what is the top number (49.8) and corresponding percentage (5.4%) referring to?

**REVIEWER NAME**	Harron, Katie
**REVIEWER AFFILIATION**	
**REVIEWER CONFLICT OF INTEREST**	None
**AI DISCLOSURE**	No
**DATE REVIEW RETURNED**	21-May-2025

**GENERAL COMMENTS**	The authors have responded to my previous comments and I have nothing further to add.

VERSION 2 – AUTHOR RESPONSE

Reviewer: 3
Prof. Katie Harron
Comments to the Author
The authors have responded to my previous comments and I have nothing further to add.
Thank you

Reviewer: 2
Dr. Florence Martin, University of Bristol
Comments to the Author

Thank you to the authors for their thoughtful responses and amendments to their important paper. Only minor suggestions to help with word count, undefined acronyms noted, and a query regarding table 1.

Abstract

Standard deviation acronym isn't defined – maybe it's not needed at all and mean is sufficient for the purpose of the abstract?
We have removed SD from the abstract
Confidence interval acronym isn't defined.
We have defined 95%CI
Results

To save words, I wonder if you could acronymise hazard ratio to HR (I know it's similar to HRT but I know you're short on word count!).
We have changed hazard ratio to HR in the results and defined this in the methods
I also don't think p-values are necessary, particularly as you want to get some words back. Fine in the abstract when you're not reporting confidence intervals, but where we can see confidence intervals not crossing one, p-values are redundant.
p-values have been removed from the main text
On page 59, standard deviation is not defined at first use in the main text.
We have moved definition of SD from the results to the first use in the methods
On page 62 of the proof, none of the new comorbidity-related acronyms (TIA, CVD, VTE etc.) have been spelt out as far as I can see (sorry not helpful for word count!).
We have defined all the acronyms in the main text now
On page 62 of the proof, odds ratio and confidence interval acronym isn't defined at first use. We have defined odds ratio, 95% CI was defined earlier in the manuscript
Consistency with how confidence intervals are reported in the text (x, x) compared to (x-x) is important (as there's a mix on page 61 and 62 for example); choosing the latter format would help save words.
This has now been changed to report all as (xx-xx)
Table 1

In the age section, what is the top number (49.8) and corresponding percentage (5.4%) referring to?
This is mean(SD) age and has been clarified in the table
Editor(s)' Comments to Author (if any):

Clinical Editor Comments to the Author:

Thank you for your revisions. When reviewing the manuscript again some further queries emerged, which we feel are important to address.

1. HRT Uptake
We have some further clarifications to request regarding the definition used in the manuscript for HRT uptake. Although the discussion and conclusions don't focus on the overall estimate of HRT uptake, it is part of the stated aim of the study and is likely to be quoted by other papers and perhaps more widely.
HRT uptake is defined as "those who had at least two prescriptions (as an indicator of likely use) of the same type of HRT from age 40 onwards (or date of registration with practice if later) at any time prior to study entry or during follow-up."
The denominator for the uptake estimate is the whole cohort - females aged 40-60 at any point between 1st January 2013 and 13th July 2023. I suspect there are many within that group for whom HRT would not be recommended - eg someone who entered the cohort by turning 40 on 12th July

2023, or women who didn't reach a level of symptom severity that would lead to them wanting help to control those symptoms (although you could argue that these should still be in the denominator).
The numerator for the HRT uptake calculation could also be questioned, defined as being 2 or more prescriptions. For example. this excludes those who tried HRT but decided not to continue (they may not have continued taking it but did they not 'take up' HRT?). Also, is getting 2 prescriptions any more meaningful a course of HRT than just 1 prescription, given the average duration of symptoms, according to the authors, is 7 years?
-Some discussion of how "HRT uptake" is defined in the wider literature would be helpful, and how these findings are (or aren't) comparable. eg within the limitations section and/or comparison with wider literature (eg HRT prescription in the last 180 days, as in the Eclipse report, which seems a much more intuitive way of looking at it). -Would you consider adding a post-hoc analysis to include the overall data for 1, 2, 3 etc prescriptions? This may not be necessary but it may reduce the risk of a single (arbitrary) number getting picked up and misused. The data appears to be available since the relative proportion who receive a second prescription compared to the # who received a single prescription according to ethnicity is reported and discussed - but the absolute (and total) numbers aren't given, as far as I can see.
- Review the the conclusion and summary box to indicate more clearly that your findings relate to a measure of HRT uptake ie being prescribed HRT two or more times, which could be understood subtly differently from simply "receiving HRT" (as stated in the abstract conclusion) which could be understood to mean being prescribed it (and taking it) at least once.
Thank you for raising this.
We carried out a sensitivity analysis reporting findings for those for whom we had a minimum of 5 years of follow-up and determined that the prescription rate in this group was 22.5%, when we restrict to those aged 45 and older at cohort entry with a minimum of 5 years follow-up the prescription rate was 23.1%. We have added the following text to the methods: “We also separately report characteristics and HRT prescriptions in those with a minimum of 5 years follow-up and in those who were aged 45 or older at cohort entry for comparison with the full cohort.”
We have included descriptive tables of these populations stratified by HRT use in the supplementary data
Regarding the minimum number of prescriptions, we have included a reference citing a previous paper that defined HRT use as receiving two or more prescriptions.
Cold S, Cold F, Jensen MB, Cronin-Fenton D, Christiansen P, Ejlertsen B. Systemic or Vaginal Hormone Therapy After Early Breast Cancer: A Danish Observational Cohort Study. J Natl Cancer Inst. 2022;114(10):1347-54.

In addition, we have reported the proportion who received at least one HRT prescription (23.2%) and the proportion who received three or more HRT prescriptions (17.2%) and re-run regression analyses for one+ and three+ prescriptions and included these sensitivity analyses in the supplementary tables. Hazard ratios and patterns are very similar to the main analysis.

2. Findings since 2016
"The aim of our study was to determine the overall uptake of HRT in women of menopausal age, describe uptake of different types of HRT prescribed and duration of use and identify factors associated with receiving an HRT prescription prior to and during the 8 years since implementation of the first NICE guidance on the menopause in 2015(8)."
Very little space is given to your findings after 2016 (supp table 8) despite this being a prominent part of your research question and the fact that the ORs for ethnicity, deprivation and region increase in this time period and are much closer to 1. Can you discuss further? Is this a positive sign?
We have added the following sentence in the discussion :
This indicates that differences in HRT prescribing by ethnicity, region of England and levels of deprivation are improving over time, but this has not been formally tested.

Other comments
Can you include your protocol (and protocol deviation log) and/or stats plan as a supplementary file? We don't currently require that observational studies include a protocol with submissions, but it seems particularly relevant to do so for this paper, to demonstrate impartial reporting of your findings according to a pre-specified plan. We have attached the study protocol as a supplementary file
Summary box:
"To date, there..." - please make this point more concise and clearer (repetition of understand)
This has been revised and it now reads:
“No previous study has reported the extent of the differences in individual HRT prescribing rates by both deprivation and ethnicity which are needed in order to address them.”

Please revise summary box according to BMJ Medicine format (3 headings - see above).
Done
Please note that for the summary box we always urge caution in making recommendations that go beyond what can be said from the evidence, particularly in observational studies (similarly, for the recommendations suggested in the discussion). Given the call for further research to understand your findings and work out causal relationships, is the conclusion in the summary box about "the need for targeted interventions to ensure equitable HRT provision" valid? We have removed the text about targeted interventions
Intro p5, 27: " suggests that there is a high clinical indicator for HRT in some ethnic groups" - review wording to consider being more specific eg that HRT may be clinically indicated in a higher proportion of Black women in the US?
We have changed the wording in the introduction as suggested
line 28-29 - perhaps simplify to simply say that this can't be generalised to other countries - readers may challenge you by saying that it is unlikely to only be demographic factors that make it difficult to generalise.
We have changed this as suggested. The sentence now begins: “Although this cannot be generalised to other countries, ”
p6, line 5 - consider revising "factors associated with" - which is a term associated with p-hacking and other methodological issues - to specify the specific factors that you've discussed as being relevant in the intro and your protocol (deprivation, ethnicity, geography)
We have revised and this now reads: “...determine whether ethnicity, deprivation and geographic region are associated with receiving an HRT prescription...”
p6 - final sentence/research question:
"The aim of our study was to determine the overall uptake of HRT in women of menopausal age, describe uptake of different types of HRT prescribed and duration of use and identify factors associated with receiving an HRT prescription prior to and during the 8 years since implementation of the first NICE guidance on the menopause in 2015(8)."-consider adding "in the UK" p9, 53 - "Higher prescribing rates in different parts of the UK were no longer statistically significant in the East Midlands, East of England, West Midlands and Yorkshire and Humber (Supplementary Table 8)." - after 2016?
We have added “in the UK” to the introduction as suggested and clarified in the results that the differences in prescribing rates in some regions are no longer significant after 2016
p11 - discussion. Please include the setting (UK) in the opening sentence of your discussion.
We have added this
Further discussion of your results in comparison with the NHS/Eclipse 2024 data (ref 36) seems warranted, given the similarities (HRT use according to different ethnicity, deprivation etc)
We were asked by a previous reviewer, Professor Martha Hickey, to not discuss our work in the context of this report for the following reasons:
“statements about HRT inequalities should not quote evidence from the all party parliamentary group. This group was funded by a pharmaceutical group who manufacture HRT. Suggest sticking with peer reviewed pubs.”

We have therefore chosen not to add this back.

p14 - discussion of regional variation: "and having a slightly lower mean age". Should this data be adjusted for age, given how much age will confound this association, and the ease with which this association could be picked up by the press, policy makers and others to suggest that it's due to other factors (eg GP services). You also mention here that it may be related to London being more ethnically diverse, however in table 2 the HR is adjusted for ethnicity We confirm that all analyses are already adjusted for age.
The text has been revised to read: “This effect remained even after adjusting for age and ethnicity in our analysis to account for the population living in London being more ethnically diverse compared to other regions of England and having a slightly lower mean age.”
p14/15 - unanswered questions. The questions suggested here don't seem to stem from this paper - despite your findings raising lots of questions for further research. Discussion of the specific risks of HRT according to ethnic group and comorbidity and decision aids seem quite removed from the aims of the paper.
The first part of this section regarding the need for more information about risks and a decision aid have been removed.
Table 1 & 2 - caption "whether they received a prescription for HRT" - this has a different meaning to 2 or more prescriptions. Please clarify. The headings have been revised to clarify that these are for 2 or more prescriptions
table 1 - 5 rows of data are given for age but only 4 rows on the first column to describe them
This has now been amended so it is clear what the first row is

Table 3 - please explain more clearly what column b refers to.
The heading has been revised to make it clear what column a and b represent
Table 3 - only 0.1% of your cohort has ADHD and >50% have taken up HRT - is this correct? if so, does it need mentioning?
We have added these numbers to the discussion to emphasise this as suggested.
Sup tables - "HRT prescriptions, N" - is this 2 or more? please check through the document that where you are measuring 2 or more prescriptions, this is clearly stated. eg ...HRT update (defined as 2 or more HRT prescriptions)
The titles of the supplementary tables have been revised to make it clear how many prescriptions they represent
Sup table 6 - would some averages for any HRT type and all ethnicities help readers make more sense of this data?
Duration of use of any formulation has now been included in the table (now Supplementary Table 7)
Sup table 8 - the description refers to HRs, but the table gives ORs. Are the ethnicity ORs adjusted for ethnicity, as the description suggests?
This should be hazard ratio and had been corrected
Fig 1 - as above, please confirm "any HRT prescription" rather than uptake of HRT (2 or more HRT prescriptions)

This is two or more prescriptions and has been corrected
Fig 2 - if all HRs are compared with the white population in quintile 1 (as is stated at the bottom) is it correct that white Q2-5 all have a HR of 1? The plots look as though you compare eg Indian Q2 with white Q2, Indian Q3 with white Q3 and so on.

This has been revised and now reads: “All hazard ratios are compared with white populations in corresponding quintile”
Please provide the typed out data for fig 1 and fig 2 either as a separate word or excel file, or below each forest plot in a table. The typed data for Figure 1 is in Table 2 and for Figure 2 is in Supplementary Table 9
